# Preparative and Kinetic Analysis of β‐1,4‐ and β‐1,3‐Glucan Phosphorylases Informs Access to Human Milk Oligosaccharide Fragments and Analogues Thereof

**DOI:** 10.1002/cbic.201900440

**Published:** 2019-12-30

**Authors:** Ravindra Pal Singh, Giulia Pergolizzi, Sergey A. Nepogodiev, Peterson de Andrade, Sakonwan Kuhaudomlarp, Robert A. Field

**Affiliations:** ^1^ Department of Biological Chemistry John Innes Centre Norwich Research Park Norwich NR4 7UH UK; ^2^ Present address: Food and Nutritional Biotechnology Division National Agri-Food Biotechnology Institute (NABI) Main Campus, Sector 81 Sahibzada Ajit Singh Nagar Punjab 140306 India; ^3^ Present address: Department of Chemistry and Manchester Institute of Biotechnology The University of Manchester 131 Princess Street Manchester M1 7DN UK

**Keywords:** enzymatic synthesis, glycans, oligosaccharides, phosphorylases

## Abstract

The enzymatic synthesis of oligosaccharides depends on the availability of suitable enzymes, which remains a limitation. Without recourse to enzyme engineering or evolution approaches, herein we demonstrate the ability of wild‐type cellodextrin phosphorylase (CDP: β‐1,4‐glucan linkage‐dependent) and laminaridextrin phosphorylase (Pro_7066: β‐1,3‐glucan linkage‐dependent) to tolerate a number of sugar‐1‐ phosphate substrates, albeit with reduced kinetic efficiency. In spite of catalytic efficiencies of <1 % of the natural reactions, we demonstrate the utility of given phosphorylase–sugar phosphate pairs to access new‐to‐nature fragments of human milk oligosaccharides, or analogues thereof, in multi‐milligram quantities.

## Graphical abstract

**Milking phosphorylases**: We demonstrate the ability of wild‐type cellodextrin phosphorylase (CDP: β‐1,4‐glucan linkage‐dependent) and laminaridextrin phosphorylase (Pro_7066: β‐1,3‐glucan linkage‐dependent) to tolerate a number of sugar‐1‐phosphate substrates, thereby enabling the construction of new‐to‐nature analogues of human milk oligosaccharides.

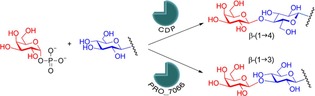

## Introduction

**Figure 1:**
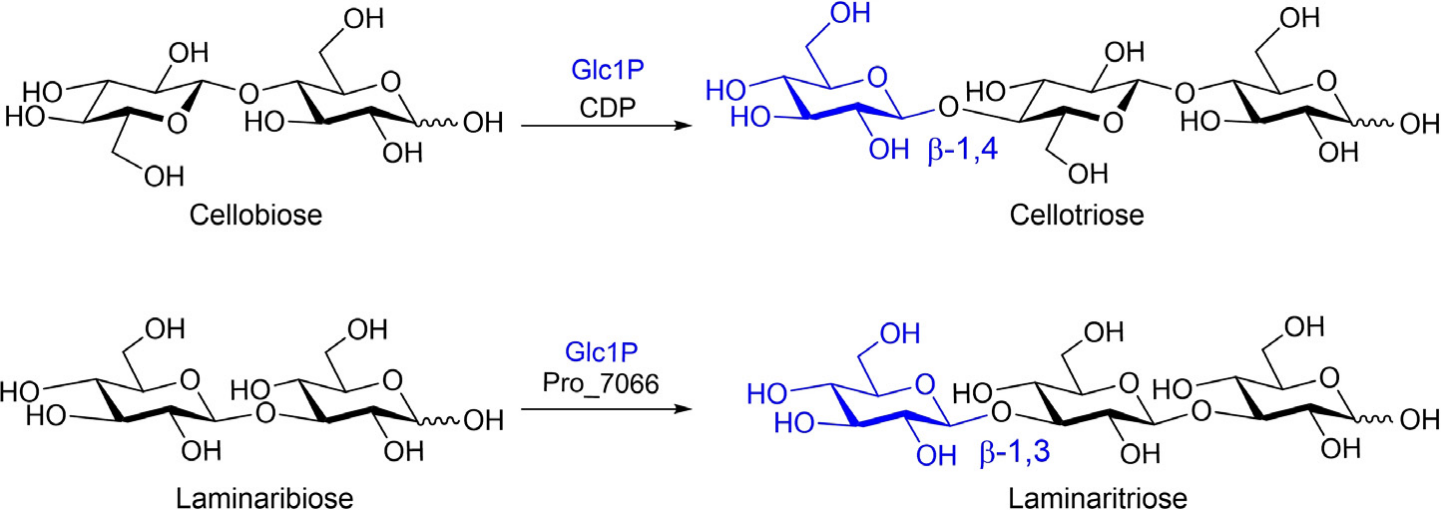
Representative reactions catalysed by CDP and Pro_7066 with glucose‐1‐phosphate (Glc1P) as donor and either cellobiose or laminaribiose as acceptor.

The synthesis of oligosaccharides and glycoconjugates remains a major challenge, although it is beginning to yield to enzymatic methods.^1^ Nonetheless, progress is hampered by the limited availability of pertinent enzymes. This is compounded by the one linkage/one enzyme notion that emanates from the early days of glycobiology, and which leads to the expectation that many more enzymes are needed to complete a catalyst arsenal.^2^ For many classes of carbohydrate‐active enzymes, low but usable catalytic efficiencies can be found for wild‐type enzymes with unnatural substrates. For instance, in earlier work, we exploited the residual catalytic activity of a sugar kinase^3^ and a *trans*‐glucosidase^4^ with unnatural substrates to produce a range of sugar nucleotide analogues and deoxyfluoromaltoses, respectively, even though the catalytic efficiencies of the processes were very low indeed—on occasion, approaching the use of milligrams of enzyme to turnover milligrams of sugar. This prompted us to challenge the catalytic capabilities of two wild‐type glycoside phosphorylases,1d which have yet to find widespread use in the synthesis of smaller glycans,^5^ as both enzymes are innate polymerases. Specifically, we investigated the action of β‐1,4‐glucan linkage‐dependent cellodextrin phosphorylase (CDP, GH94)^6^ and β‐1,3‐glucan linkage‐dependent laminaridextrin phosphorylase (Pro_7066, GH149;^7^ natural reactions shown in Figure 1) in reactions with a range of both natural and unnatural sugar‐1‐phosphate donors and glucan acceptor substrates.

Although phosphorylases are generally thought to be involved physiologically in the degradation of di‐ or oligosaccharides, by feeding elevated levels of sugar‐1‐phosphate or removing inorganic phosphate by‐products, preparatively useful reactions can be accomplished. This is particularly well exemplified by the commercial, kilogram‐scale production of the cosmetic humectant α‐glucosyl glycerol by using sucrose phosphorylase.^8^ α‐1,4‐Glucan phosphorylase has been used with variants of its natural donor substrate to synthesise unnatural oligo‐ and polysaccharides;^9^ α‐1,4‐glucan polymer‐modified nanomaterials can also be accessed.^10^ In a similar way, CDP‐catalysed reactions provide access to β‐1,4‐glucan‐linked, cellulose‐like materials.^11^, ^12^ In terms of single sugar addition, as opposed to oligo/polymerisation reactions with its natural substrate Glc1P, CDP has been shown to be capable of using the anomeric phosphates of xylose (synthesis of a library of β‐(1,4) hetero‐oligosaccharides),^13^ galactose (biocatalytic production of novel glycolipids)^14^ and glucosamine (microscale reaction; preparative utility not demonstrated).^6^ We recently identified algal and bacterial β‐1,3‐glucan phosphorylases (e.g., Pro_7066) that are capable of producing β‐1,3‐glucan oligosaccharides; no assessment of their donor substrate specificity has been reported to date.^7^, ^15^

In selecting sugar phosphates to assess with CDP and Pro_7066, we considered the synthesis of fragments of human milk oligosaccharides (HMOs), which have been the focus of recent enzymatic syntheses.^16^ The synthesis of HMO‐like molecules requires the ability to incorporate galactose, which is a prerequisite for later fucosylation or sialylation. In addition, we wanted to install sugar units that could support rapid library synthesis or have an impact on branching^17^ (glucosamine; enabling protecting group‐free N‐derivatisation), or that might modify the rate of microbiome‐mediated digestion^18^ (β‐mannose, which is less common in oligosaccharides). Hence, we arrived at Glc1P, α‐d‐galactose‐1‐phosphate (Gal1P), α‐d‐glucosamine‐1‐phosphate (GlcN1P) and α‐d‐mannose‐1‐phosphate (Man1P) as prospective donor substrates. As the latter two compounds are not commercially available at reasonable cost, we report the synthesis of these compounds in the Supporting Information. We have recently reported on structural rationalisation of the promiscuity of laminaribiose phosphorylase, which produces disaccharides Glc‐β‐1,3‐Glc and Man‐β‐1,3‐Glc when fed glucose and Glc1P or Man1P, respectively.^19^

## Results and Discussion

### Evaluation of the kinetic efficiency of CDP and Pro_7066 with a panel of sugar phosphates

In initial kinetic evaluations, both CDP and Pro_7066 were effective at transferring Glc from Glc1P onto either β‐1,3‐linked laminaribiose (**4**) or β‐1,4‐linked cellobiose (**1**) to generate new β‐1,4‐ and β‐1,3‐linkages, respectively. Turnover numbers were of the order of a few per second (Table 1 and Figure S7 in the Supporting Information). With noncognate sugar phosphate donor substrates, however, greater distinction was seen between the enzymes. With Gal1P, *k*_cat_ dropped ≈25‐fold compared to Glc1P for both enzymes, while *K*_m_ increased approximately threefold for CDP and about 10‐ to 45‐fold for Pro_7066, depending on the acceptor. With GlcN1P, *k*_cat_ dropped more dramatically, at ≈125‐fold lower than Glc1P for both enzymes. However, whereas *K*_m_ increased only slightly for CDP, for Pro_7066 it decreased ≈70‐fold, indicating much stronger recognition of GlcN1P by Pro_7066 than by CDP. In turn, this is reflected in the much greater kinetic efficiency of Pro_7066 than CDP when using GlcN1P as a donor substrate. Tolerance of C2 modification of donor substrate by Pro_7066 is also borne out with C2 axial OH Man1P, which has a respectably low‐millimolar *K*_m_ for Pro_7066 and *k*_cat_ ≈200‐fold down on Glc1P, whereas CDP does not show turnover with the *manno*‐configured sugar‐1‐phosphate.

**Table 1:** Kinetic parameters of cellodextrin phosphorylase (CDP) and laminarin phosphorylase (Pro_7066)

Enzyme	Donor	Acceptor	Kappm	kappcat	*k*_cat_/*K*_m_	Relative
			[mm]	[s^−1^]	[s^−1^ mm^−1^]	*k*_cat_/*K*_m_ [%]
CDP	Glc1P	**4**	3.0±0.3	15.9±0.4	5.3	100
		**1**	3.0±0.6	16.4±0.7	5.5	100
	Gal1P	**4**	10.7±0.7	0.60±0.01	0.06	1.1
		**1**	9.3±1.1	0.60±0.02	0.06	1.1
	GlcN1P	**4**	5.1±1.2	0.14±0.013	0.03	0.6
		**1**	1.6±0.2	0.08±0.003	0.05	0.9
	Man1P	**4**	n.a.	n.a.	n.a.	n.a.
		**1**	n.a.	n.a.	n.a.	n.a.
Pro_7066	Glc1P	**4**	3.1±0.7	9.3±0.5	3.0	100
		**1**	2.5±0.7	10.6±0.6	4.2	100
	Gal1P	**4**	31.6±2.4	0.46±0.02	0.015	0.5
		**1**	136.4±23.6	0.77±0.10	0.006	0.1
	GlcN1P	**4**	0.05±0.01	0.09±0.002	1.8	60
		**1**	0.04±0.01	0.04±0.001	1.0	24
	Man1P	**4**	2.5±0.2	0.15±0.006	0.060	2
		**1**	1.7±0.7	0.03±0.004	0.018	0.4

n.a.: not applicable. All the reactions were performed in triplicate.

**Figure 2:**
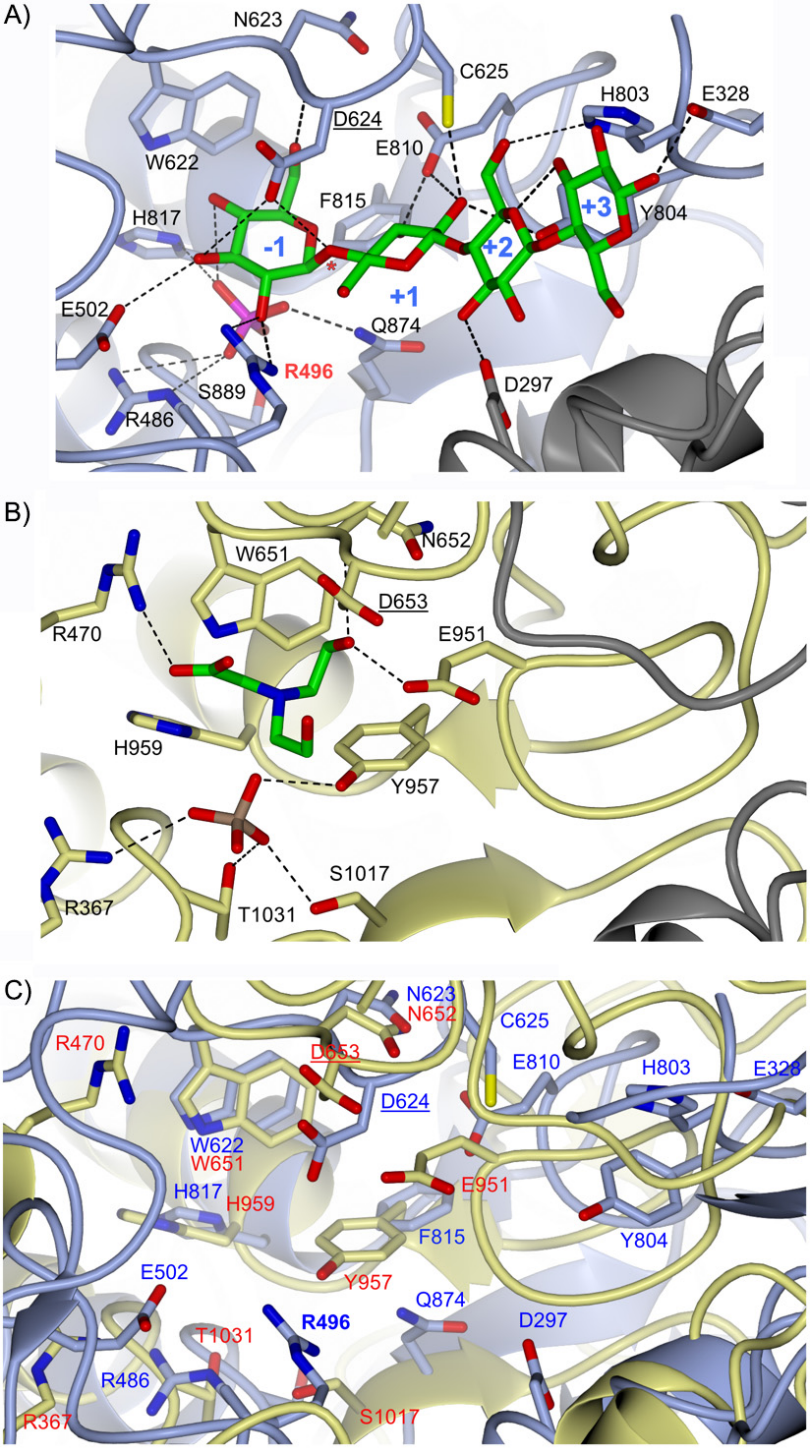
Comparison between the active sites of A) CDP (PDB ID: 5NZ8) and B) Pro_7066 (PDB ID: 6HQ6). C) Superposition of CDP (blue backbone) and Pro_7066 (beige backbone). Both proteins are homodimers; one subunit is shown in blue (for CDP) or beige (in Pro_7066) and the adjacent subunits in grey. Protein backbones are shown in cartoon representation, and ligands and protein side chains in cylinder form. Phosphate is coloured in pink, sulfate in brown, oxygen in red and carbon on cellotetraose and BICINE in green. Catalytic residues are underlined, and the cleavage site in CDP is indicated by asterisk in (A). The binding subsites are indicated by numbers. The amino acids in (C) are labelled in red for Pro_7066 and blue for CDP.

In order to rationalise the difference in the tolerance towards C2 modification of sugar 1‐phosphates by CDP and Pro_7066, we compared the reported X‐ray crystal structures of the two proteins (PDB IDs: 5NZ8 and 6HQ6, respectively),^6^, ^20^ although structures of complexes with donor substrate are not yet available. In the structure of CDP in complex with cellotetraose and phosphate, the hydroxy group at C2 of the nonreducing terminal Glc residue occupying the sugar 1‐phosphate binding site (−1 subsite) makes two hydrogen bond contacts with R496 (Figure 2 A, red). The presence of the charged R496 side chain could impact on the NH_2_ of GlcN1P, thereby explaining the dramatic drop in *k*_cat_/*K*_M_ of CDP for GlcN1P. Superposition of the CDP and Pro_7066 structures, with the latter containing BICINE and sulfate molecules, showed that they are dissimilar (RMSD>3 Å; Figure 2 C); this is not surprising considering the fact that there is no significant sequence similarity between the two proteins (*E* value=0.5, query coverage=23 %). However, active‐site alignments based on conserved catalytic aspartate and tryptophan residues, and phosphate/sulfate residues sitting in the pockets thought to be occupied by phosphate in sugar 1‐phosphates, leads to the conclusion that there is no obvious equivalent to the CDP R496 residue in the Pro_7066 active site (Figure 2 B). When Man1P is used as a sugar donor, the hydrogen bond contacts between C2‐OH and R496 are likely to be broken because of the change in orientation of the OH from equatorial to axial. The lack of hydrogen bond would be expected to have a severe impact on the overall interaction between Man1P and the CDP active site, resulting in the observed lack of turnover when Man1P was used as a substrate. Again, active‐site alignment of CDP and Pro_7066 shows no obvious hydrogen‐bonding interaction or steric impediment to Glc1P/Man1P binding to the latter; this might account for the ability of Pro_7066 to use Man1P as a donor substrate.

### Evaluation of the chemical competence of CDP and Pro_7066 with noncognate sugar phosphates and a range of acceptors

In order to maximise synthetic utility, the pH and temperature optima for CDP and Pro_7066 reactions were established (Figure S1). All further experiments were therefore conducted at 37 °C and pH 5.0 (CDP) or pH 7.0 (Pro_7066). We initially investigated Gal1P as a donor and natural disaccharide acceptors cellobiose **1** and laminaribiose **4** for CDP and Pro_7066, respectively. Given the axial orientation of OH‐4 in Gal, once Gal is transferred onto an acceptor, further extension of oligosaccharide stops for CDP (Figure S2) due to the strict requirement for an equatorial OH‐4 in the acceptor. The Pro_7066 galactosylation reaction products, however, were more complex, with a series of oligomeric products being obtained (see section below).

In general terms, CDP (Table 2) showed a preference for shorter acceptor substrates over longer ones (compare acceptors **1** and **4** vs. **10**–**13** vs. **14**–**16**), regardless of the acceptor intersugar linkages, whereas Pro_7066 (Table 3) showed a less consistent preference for acceptor size but was more sensitive to the linkages present in the acceptor. In particular, whereas Pro_7066 will tolerate a nonreducing terminal Glc‐β‐1,4‐Glc arrangement in an acceptor (compare acceptors **1** and **4**), it is less effective if the β‐1,4‐linkage is located at an internal position of the acceptor (compare acceptors **2** and **18**).

**Table 2:** Cellodextrin phosphorylase (CDP)‐catalysed syntheses of oligosaccharides 9–17 using acceptors 1–8 and Gal1P and GlcN1P as glycosyl donors.

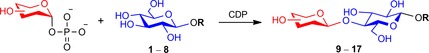				
Donor	Acceptor	R	Product	Yield [%]
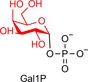	**1**	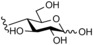	**9**	95
	**2**	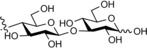	**10**	70
	**3**	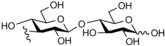	**11**	60
	**4**	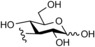	**12**	81
	**5**	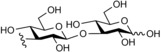	**13**	60
	**6**	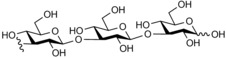	**14**	31
	**7**	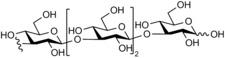	**15**	19
	**8**	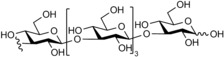	**16**	16
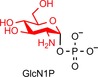	**4**	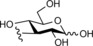	**17**	88

The structures of all enzymatically synthesised oligosaccharides were confirmed by HRMS (ESI) and ^1^H and ^13^C NMR spectroscopy. Only two of these compounds have previously been described in the literature: tri‐ and tetrasaccharides **9** and **11** were synthesised by using the loose acceptor specificity of β‐1,4‐galactosyltransferase.^24^

**Table 3:** Pro_7066‐catalysed syntheses of oligosaccharides **19**–**25** with acceptors **1**–**4** and **18** and Gal1P, GlcN1P and Man1P as glycosyl donors.

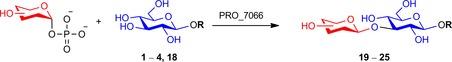				
Donor	Acceptor	R	Product	Yield [%]
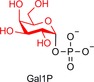	**4**	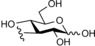	**19** ^[a]^	38^[a]^
	**3**	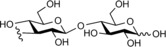	**20**	22^[a]^
	**1**	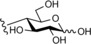	**21**	77
	**18**	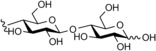	**22**	47
	**2**	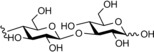	**23**	80
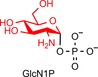	**1**	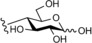	**24**	74
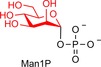	**1**	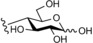	**25**	94

[a] Low yield due to acceptor phosphorolysis. Disaccharide **19** was previously reported as a product of *Acetobacter* exopolysaccharide fragmentation.^23^

### Disproportionation of glucan acceptors

**Figure 3:**
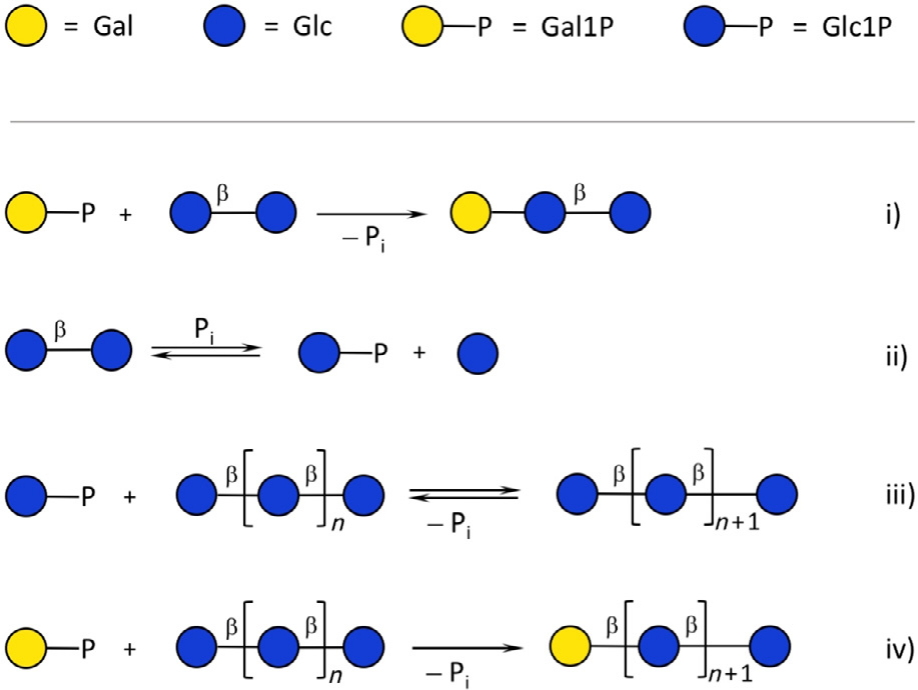
Phosphorylase‐catalysed galactosylation of β‐linked glucose disaccharide substrates by Pro_7066 results in the formation of a series of extended glucans capped with a β‐Gal residue. i) Trisaccharide formation accompanied with release of inorganic phosphate P_i_; ii) generation of Glc1P; iii) disproportionation of β‐linked glucose disaccharide, priming the formation of glucose‐based oligosaccharides; iv) galactosylation of extended glucose‐based oligosaccharides with Gal1P.

After careful analysis of glycan products by thin‐layer chromatography (TLC) and MALDI‐TOF MS, it became evident that Pro_7066 stops after the addition of a single galactose residue onto laminaribiose (Figure 3 i), thus indicating that a configurational change at C‐4 is sufficient to block efficient glycosylation of the adjacent OH‐3 group. However, phosphorylase‐catalysed reactions with a kinetically inefficient donor Gal1P and a natural acceptor are complicated by the phosphate‐mediated disproportionation of the oligosaccharide acceptor. Inorganic phosphate, liberated as a result of glycosylation with Gal1P, can participate in the degradative phosphorolysis of β‐linked glucose disaccharide acceptors leading to in situ production of Glc1P (Figure 3 ii). Given the greater kinetic efficiency with which natural donor Glc1P can be used by CDP and Pro_7066 (Table 1),^6^, ^7^ a series of extended glucose‐based oligosaccharides, together with glucose, might be formed in these reactions (Figure 3 iii). Some of these oligosaccharides could become capped with galactose, thus leading to a mixture of products (Figure 3 iv). Similar disproportionation reactions have been observed with mammalian muscle α‐1,4‐glucan phosphorylases,^21^ and similarly when GlcN‐1‐P was employed.^22^

This situation proved to be much less problematic in reactions with CDP than with Pro_7066 (Figures 3, S2 and S3), which in our hands had an undetectable propensity for disproportionation of cellobiose, but could still synthesise this disaccharide. Nonetheless, these results highlight the need for careful consideration of acceptor substrate linkages when using kinetically inefficient donor substrates: compare the isolated product yields of **19** (38 %) and **21** (77 %) for Pro_7066 reactions with acceptors possessing linkages that either match or do not match the enzyme specificity, respectively.

### Reactions with matched linkage acceptors

β‐1,4‐Linked di‐ and trisaccharides **1** and **2** can be considered “matched” acceptors for β‐1,4‐linkage‐specific CDP, whereas β‐1,3‐linked di‐ and trisaccharides **4** and **3** are “matched” for β‐1,3‐linkage‐specific Pro_7066. Under optimal conditions, CDP‐catalysed reactions of Gal1P with acceptors **1** and **2** led to β‐1,4‐linked products **9** and **10** in 95 and 70 % yield, respectively (Table 2). As was evident from TLC and MALDI‐TOF MS analyses (Figure S3), the action of Pro_7066 on Gal1P and acceptors **4** and **3** was quite different, resulting in a range of β‐1,3‐glucosyl‐ and β‐1,3‐galactosyl‐terminated oligosaccharides, depending on the acceptor and the ratio of acceptor and Gal1P donor used. In this case, in order to facilitate the purification of target trisaccharide **19** and tetrasaccharide **20** (from acceptors **4** and **3**, respectively), the reaction mixtures were first treated with β‐1,3‐glucanase and then subjected to gel‐permeation chromatography. The yields of **19** (38 %) and **20** (22 %; Table 3) were moderate compared to the corresponding yields of isomeric compounds **9** and **10** from CDP‐catalysed galactosylation (Table 2).

### Reactions with unmatched linkage acceptors

Learning from the results of phosphorylases′ action on Gal1P and matched acceptors, we anticipated that having an unmatched linkage at the nonreducing terminus of the acceptor (i.e., a β‐1,3‐linked acceptor for β‐1,4‐specific CDP or a β‐1,4‐linked acceptor for β‐1,3‐specific Pro_7066) would yield single products, as reactions would not be complicated by the phosphorolysis of acceptors. Indeed, incubating reaction mixtures over three days with periodic product analysis by TLC and MALDI‐TOF MS, we were able to synthesise several further β‐galactosylated compounds **11**–**17** and **19**–**23** in moderate to high yield (16–81 %, Tables 2 and 3). In the case of β‐1,4‐galactosylation of a series of β‐1,3‐linked oligosaccharides by CDP, increasing acceptor chain length led to a reduction in yield from 81 to 16 % on going from disaccharide **4** through to pentasaccharide **8** (Table 2 and Figure S6).

The efficiencies of phosphorylase‐catalysed reactions with acceptors having both β‐1,4 and β‐1,3 linkages between glucose residues were variable. Yields of CDP‐catalysed β‐1,4‐galactosylation were not influenced by a mixed‐linked acceptor: reaction with both Glc‐β‐1,3‐Glc‐β‐1,4‐Glc (**3**) and Glc‐β‐1,3‐Glc‐β‐1,3‐Glc (**5**) resulted in 70 and 60 % yields of products **10** and **13**, respectively. In contrast, β‐1,3‐galactosylation of mixed‐linked trisaccharides Glc‐β‐1,4‐Glc‐β‐1,3‐Glc (**2**), Glc‐β‐1,3‐Glc‐β‐1,4‐Glc (**3**) and Glc‐β‐1,4‐Glc‐β‐1,4‐Glc (**18**) by Pro_7066 gave 80, 22 and 47 % yields, respectively, thus indicating that Pro_7066 is more sensitive to acceptor oligosaccharide linkages than is CDP.

### Other sugar phosphate donors

Unnatural donor substrate glucosamine 1‐phosphate (GlcN1P), which has been used with α‐1,4‐glucan phosphorylase to synthesis α‐linked chitosan analogues,22b was also investigated in reactions with CDP and Pro_7066. It appeared to be an excellent donor for glycosylation with both enzymes, producing trisaccharide **17** in CDP‐catalysed reaction with **4** and trisaccharide **24** in Pro_7066‐catalysed reaction with **1** in 88 and 74 % yields, respectively. In CDP reactions, very low, but detectable, quantities of glycan arising from the incorporation of two consecutive GlcN residues was observed, but these reactions were not optimised.

Changing GlcN1P to galactosamine‐1‐phosphate (GalN1P) had a profound negative effect on glycosylation efficiency: the latter compound was not accepted as a substrate by Pro_7066, and CDP showed only very low activity with this prospective donor substrate. Having the amino group acetylated, as in *N*‐acetyl‐glucosamine‐1‐P (GlcNAc1P), was tolerated to some degree by CDP and not at all by Pro_7066, as was evident from TLC and MALDI‐TOF MS analyses (Figures S4 and S5). We recently showed that a *Paenibacillus* laminaribiose phosphorylase has a relaxed substrate specificity and can accommodate mannose 1‐phosphate (Man1P) as a glycosyl donor.^19^ We performed similar assessment of both CDP and Pro_7066 and found that only Pro_7066 can tolerate a change of the donor C‐2 configuration: the reaction of Man1P with cellobiose led to β‐1,3‐mannoside **22** in near quantitative yield. This trisaccharide was previously identified as a component of the exopolysaccharide of cellulose‐producing bacterium *Acetobacter xylinum*.^23^

### NMR characterisation of enzymatically synthesised oligosaccharides

The confirmation of the stereochemical and regiochemical outcomes of CDP and Pro_7066 reactions were confirmed by NMR spectroscopy, although considerable signal overlap in both the ^1^H and ^13^C NMR spectra of galactosylated oligosaccharides prevented detailed assignment of all resonances. However, it was possible to pinpoint characteristic signals of nonreducing terminal residues present in **9**–**17** and **19**–**25** (see the Supporting Information) but absent in the spectra of the corresponding oligosaccharide precursors **1**–**8**. Mostly with the aid of 2D HSQC spectra, the β‐galactopyranosyl residue could be identified by the presence of resonances at *δ*=102–103 for C‐1β and *δ*=68.2–68.6 for C‐4 in ^13^C NMR spectra. In addition, a characteristic doublet of H‐4 with ^3^ *J*<2 Hz at *δ*=3.7–3.9 and an anomeric signal at *δ*=4.3–4.5 supported the presence of β‐Gal residues in structures **9**–**16** and **19**–**23**. The 1,4‐galactosylation of glucan acceptors was confirmed through observation of a characteristic signal at about *δ*=78.0 ppm, which appeared 2–3 ppm downfield from the often‐crowded region of pyranose ring carbons and belonged to C‐4 of the nonreducing termini of the corresponding acceptors. The regiochemistry of Prozo_7066‐catalysed 1,3‐galactosylation, leading to oligosaccharides **19**–**23**, followed from the appearance of signals at *δ*=84–85 ppm, which are diagnostic of C‐3 of glucopyranose bearing a glycosyl residue at that position. In a similar manner, the above characteristic chemical shifts of C‐4 and C‐3 were used to confirm the presence of β‐GlcN‐1,4‐Glc and β‐GlcN‐1,3‐Glc linkages in trisaccharides **17** and **24**, respectively, as wells as the β‐Man‐1,3‐Glc linkage in trisaccharide **25**. The size of the oligosaccharide was confirmed by HRMS in all instances.

## Conclusion

In conclusion, we have assessed the kinetic efficiency of wild‐type β‐1,4‐glucan (cellodextrin) phosphorylase CDP and β‐1,3‐glucan (laminaridextrin) phosphorylase Pro_7066 with a range of sugar‐1‐phoshates substrates; the outcomes of the reactions are tentatively explained on the basis of protein X‐ray crystal structures for both enzymes. These kinetic studies highlight the potential of the native enzymes to use sugar‐1‐phosphates other than Glc1P, and we have gone on to demonstrate the multi‐milligram‐scale production of novel oligosaccharides capped with either Gal, GlcN or Man in a series of examples that provide building blocks for the generation of analogues of human milk oligosaccharides. Although the kinetic efficiencies of these processes are often <1 % of that of the natural reaction—a feature that allows single turnover reactions even though these enzymes are naturally polymerases—the enzymes are easily produced in tens‐of‐milligram quantities from one litre cultures of transformed *Escherichia coli*. These studies highlight that the notion of one enzyme/one linkage does not hold for the phosphorylases, which, although stereo‐ and regiospecific, are not limited to use with only one donor substrate, if you push them hard enough. In turn, this restricts the range of enzymes that are required for the in vitro synthesis of β‐1,4 (CDP) and β‐1,3 (Pro_7066) linkages in the nonreducing terminal position of some classes of oligosaccharide.

## Conflict of interest

*The authors declare no conflict of interest*.

## Supporting information

As a service to our authors and readers, this journal provides supporting information supplied by the authors. Such materials are peer reviewed and may be re‐organized for online delivery, but are not copy‐edited or typeset. Technical support issues arising from supporting information (other than missing files) should be addressed to the authors.

Supplementary
