# Supplementary material for: Preparative and Kinetic Analysis of β‐1,4‐ and β‐1,3‐Glucan Phosphorylases Informs Access to Human Milk Oligosaccharide Fragments and Analogues Thereof
Source: Chembiochem. 2019 Dec 30;21(7):1043–9. doi: 10.1002/cbic.201900440 (PMC7187349; doi:10.1002/cbic.201900440)
Supplement: Supplementary file 1 — Supplementary [file CBIC-21-1043-s001.pdf]

CHEMBIOCHEM

Supporting Information

Preparative and Kinetic Analysis of β-1,4- and β-1,3-Glucan Phosphorylases Informs Access to Human Milk Oligosaccharide Fragments and Analogues Thereof

Ravindra Pal Singh+,[a, b] Giulia Pergolizzi+,[a] Sergey A. Nepogodiev,[a] Peterson de Andrade,[a] Sakonwan Kuhaudomlarp,[a] and Robert A. Field*[a, c]

cbic_201900440_sm_miscellaneous_information.pdf

Supplementary Information for

Preparative and kinetic assessment of β-1,4- and β-1,3-glucan phosphorylases informs access to human milk oligosaccharide fragments and analogues thereof

Ravindra Pal Singh, Giulia Pergolizzi, Sergey A. Nepogodiev, Peterson de Andrade,
Sakonwan Kuhaudomlarp, Robert A. Field*

*Corresponding author e-mail: rob.field@jic.ac.uk; robert.field@manchester.ac.uk

Table of Contents

1. General methods 3
2. Recombinant protein production 4
3. Phosphorylase activity assay 4
4. Optimization of enzymatic reactions for the synthesis of terminal galactoside oligosaccharides 4
5. CDP-catalysed reactions of Gal1P with cognate acceptors **1** and **2** 5
6. Pro_7066-catalysed reactions of Gal1P with cognate acceptors **3** and **4** 6
7. CDP and Pro_7066 reactions with non-cognate acceptors and donors 9
8. Influence of acceptor chain length on the enzymatic galactosylation of glucans 10
9. Kinetic analysis of donor specificities in reactions catalysed by CDP and Pro_7066 11
10. Chemical synthesis of sugar 1-phosphates 15
    α-D-mannopyranose-1-phosphate bis(triethylammonium salt) (Man1P) 15
    2-Amino-2-deoxy-α-D-glucopyranose-1-phosphate disodium salt (GlcN1P) 16
11. Charactrisaction of oligosaccharides synthesised by enzymatic glycosylation with CDP and Pro_7066 .. 17
    β-D-Gal^{iii}-(1→4)-β-D-Glc^{ii}-(1→4)-D-Glc^{i} (**9**) 17
    β-D-Gal^{iv}-(1→4)-β-D-Glc^{iii}-(1→4)-β-D-Glc^{ii}-(1→3)-D-Glc^{i} (**10**) 17
    β-D-Gal^{iv}-(1→4)-β-D-Glc^{iii}-(1→3)-β-D-Glc^{ii}-(1→4)-D-Glc^{i} (**11**) 17
    β-D-Gal^{iii}-(1→4)-β-D-Glc^{ii}-(1→3)-D-Glc^{i} (**12**) 17
    β-D-Gal^{iv}-(1→4)-β-D-Glc^{iii}-(1→3)-β-D-Glc^{ii}-(1→3)-D-Glc^{i} (**13**) 18
    β-D-Gal^{v}-(1→4)-β-D-Glc^{iv}-(1→3)-β-D-Glc^{iii}-(1→3)-β-D-Glc^{ii}-(1→3)-D-Glc^{i} (**14**) 18
    β-D-Gal^{vi}-(1→4)-β-D-Glc^{v}-(1→3)-β-D-Glc^{iv}-(1→3)-β-D-Glc^{iii}-(1→3)-β-D-Glc^{ii}-(1→3)-D-Glc^{i} (**15**) 18

β-D-Gal^{vii}-(1→4)-β-D-Glc^{vi}-(1→3)-β-D-Glc^{v}-(1→3)-β-D-Glc^{iv}-(1→3)-β-D-Glc^{iii}-(1→3)-β-D-Glc^{ii}-(1→3)-D-Glc^{i} (**16**) 18
β-D-GlcN^{iii}-(1→4)-β-D-Glc^{ii}-(1→3)-D-Glc^{i} (**17**) 19
β-D-Gal^{iii}-(1→3)-β-D-Glc^{ii}-(1→3)-D-Glc^{i} (**19**) 19
β-D-Gal^{iv}-(1→3)-β-D-Glc^{iii}-(1→3)-β-D-Glc^{ii}-(1→4)-D-Glc^{i} (**20**) 19
β-D-Gal^{iii}-(1→3)-β-D-Glc^{ii}-(1→4)-D-Glc^{i} (**21**) 19
β-D-Gal^{iv}-(1→3)-β-D-Glc^{iii}-(1→4)-β-D-Glc^{ii}-(1→4)-D-Glc^{i} (**22**) 20
β-D-Gal^{iv}-(1→3)-β-D-Glc^{iii}-(1→4)-β-D-Glc^{ii}-(1→3)-D-Glc^{i} (**23**) 20
β-D-GlcN^{iii}-(1→3)-β-D-Glc^{ii}-(1→4)-D-Glc^{i} (**24**) 20
β-D-Man^{iii}-(1→3)-β-D-Glc^{ii}-(1→4)-D-Glc^{i} (**25**) 20
12. References 21
13. NMR spectra of sugar phosphates 22
α-D-Mannopyranose-1-phosphate bis(triethylammonium salt) 22
Amino-2-deoxy-α-D-glucopyranose-1-phosphate disodium salt 24
14. NMR characterization of oligosaccharides synthesised by enzymatic glycosylation with CDP and Pro_7066 26
β-D-Gal^{iii}-(1→4)-β-D-Glc^{ii}-(1→4)-D-Glc^{i} (**9**) 26
β-D-Gal^{iv}-(1→4)-β-D-Glc^{iii}-(1→4)-β-D-Glc^{ii}-(1→3)-D-Glc^{i} (**10**) 28
β-D-Gal^{iv}-(1→4)-β-D-Glc^{iii}-(1→3)-β-D-Glc^{ii}-(1→4)-D-Glc^{i} (**11**) 30
β-D-Gal^{iii}-(1→4)-β-D-Glc^{ii}-(1→3)-D-Glc^{i} (**12**) 32
β-D-Gal^{iv}-(1→4)-β-D-Glc^{iii}-(1→3)-β-D-Glc^{ii}-(1→3)-D-Glc^{i} (**13**) 34
β-D-Gal^{v}-(1→4)-β-D-Glc^{iv}-(1→3)-β-D-Glc^{iii}-(1→3)-β-D-Glc^{ii}-(1→3)-D-Glc^{i} (**14**) 36
β-D-Gal^{vi}-(1→4)-β-D-Glc^{v}-(1→3)-β-D-Glc^{iv}-(1→3)-β-D-Glc^{iii}-(1→3)-β-D-Glc^{ii}-(1→3)-D-Glci (**15**) 38
β-D-Gal^{vii}-(1→4)-β-D-Glc^{vi}-(1→3)-β-D-Glc^{v}-(1→3)-β-D-Glc^{iv}-(1→3)-β-D-Glc^{iii}-(1→3)-β-D-Glc^{ii}-(1→3)-D-Glc^{i} (**16**) 40
β-D-GlcN^{iii}-(1→4)-β-D-Glc^{ii}-(1→3)-D-Glc^{i} (**17**) 42
β-D-Gal^{iii}-(1→3)-β-D-Glc^{ii}-(1→3)-D-Glc^{i} (**19**) 44
β-D-Gal^{iv}-(1→3)-β-D-Glc^{iii}-(1→3)-β-D-Glc^{ii}-(1→4)-D-Glc^{i} (**20**) 46
β-D-Gal^{iii}-(1→3)-β-D-Glc^{ii}-(1→4)-D-Glc^{i} (**21**) 48
β-D-Gal^{iv}-(1→3)-β-D-Glc^{iii}-(1→4)-β-D-Glc^{ii}-(1→4)-D-Glc^{i} (**22**) 50
β-D-Gal^{iv}-(1→3)-β-D-Glc^{iii}-(1→4)-β-D-Glc^{ii}-(1→3)-D-Glc^{i} (**23**) 52
β-D-GlcN^{iii}-(1→3)-β-D-Glc^{ii}-(1→4)-D-Glc^{i} (**24**) 54
β-D-Man^{iii}-(1→3)-β-D-Glc^{ii}-(1→4)-β-D-Glc^{i} (**25**) 56

1. General methods

All chemicals including sugar-1-phosphates were purchased from Sigma-Aldrich (UK) and used as received, unless otherwise stated. Oligosaccharide substrates for the enzymatic reactions were purchased from Carbosynth Limited (Berkshire UK) or Megazyme (Wicklow, Ireland), unless otherwise noted. Milli-Q H_2O was used for the preparation of aqueous buffers. All reagents and solvents used for analytical applications were of analytical quality. TLC was performed on Silica Gel 60 F254 (Merck). Compounds were visualised by spraying TLC with orcinol solution (20 mg/ml orcinol monohydrate in EtOH-H_2SO_4-H_2O 75:10:5, v/v/v), followed by heating.

Product characterisation. Reaction products were characterised by TLC, MALDI-ToF MS on a Bruker Autoflex Speed spectrometer using flexControl - autoflex TOF/TOF and flexAnalysis software or by low resolution ESI MS on an Advion Expression Compact Mass Spectrometer (CMS). High-resolution ESI MS were obtained on a Waters Synapt G2-Si mass spectrometer. ^{1}H, ^{13}C-DEPT, 2D-COSY and 2D-HSQC NMR spectra were recorded on Bruker Avance III 400 MHz spectrometer equipped with a broadband BBFO probe or on Bruker Neo 600 MHz spectrometer equipped with a TCI cryoprobe. Chemical shifts (δ) are reported in parts per million (ppm) using residual solvent signal for referencing. Colorimetric assays were performed in NUNC 96 plates on a BMG Labtech FLUOStar Omega microplate reader equipped with suitable absorbance filters.

Sample preparation for analysis of products of enzymatic reactions by MALDI ToF. Reaction mixture (5 µl) was diluted with Milli-Q H_2O (5 µl), TMD-8 (Sigma) mixed bed resin was added, followed by incubation at room temp. for 5 min. Samples were typically mixed with equal volume of2,5-dihydroxybenzoic acid (DHB) matrix (10 mg/ml in 30% acetonitrile + 0.1% TFA in H_2O) and spotted on a target plate (Bruker MTP 384 Polished Steel TF Target), then analysed in positive mode.

HRMS analysis procedure. Samples were diluted into 50% methanol-0.1% formic acid and infused into the mass spectrometer at 5–10 µl/min using a Harvard Apparatus syringe pump. The mass spectrometer was controlled by Masslynx 4.1 software (Waters). It was operated in high-resolution positive ion mode and calibrated using sodium formate. The sample was analysed for 2 min with 1 s MS scan time over the range of 50–1200 m/z (or as appropriate) with 3.5 kV capillary voltage, 40 V cone voltage, 100 °C cone temperature. Leu-enkephalin peptide (1 ng/ml, Waters) was infused at 10 µl/min as a lock mass (m/z 556.2766) and measured every 10 s. Spectra were generated in Masslynx 4.1 by combining several scans, and peaks were centred using automatic peak detection with lock mass correction.

Purification techniques. Ion exchange chromatography was performed using Bioscale™ Mini Macro-Prep cartridges (Bio-Rad, UK), High Q for neutral oligosaccharides and High S for glucosamine-containing compounds respectively, and a step-gradient from 0 to 1 M ammonium bicarbonate buffer (pH 9.4). Product containing fractions were combined and reduced to dryness. The residue was co-evaporated repeatedly with methanol to remove residual ammonium bicarbonate. Gel Filtration Chromatography was performed with a Perkin Elmer series 200 HPLC, equipped with a refractive index detector and a fraction collector, using as stationary phase a Toyopearl TSK-HW40S column (100 cm × 2.2 cm) at 40 °C. Products were eluted with Milli-Q H_2O at a flow-rate of 0.5 ml/min and collected in 2 mL fractions. If necessary, further purification was carried out by solid phase extraction using C-18 Sep-Pak cartridges (Waters) with a gradient elution of acetonitrile against Milli-Q H_2O. Other purification methods are specified in appropriate sections below.

2. Recombinant protein production

A recombinant plasmid (pET15b) containing the CDP gene from *Ruminiclostridium thermocellum* YM4 strain (GenBank accession number BAB71818) was transformed into *E. coli* BL21 (DE3) cells and the transformant grown according to the literature.[1] Briefly, transformant in 1 L of LB medium containing appropriate antibiotic (carbenicillin, 100 µg/ml) was incubated at 37 °C with gentle shaking until an OD_{600} of about 0.6. Heterologous protein expression was induced by adding isopropyl β-D-1-thiogalactopyranoside (IPTG) at 1 mM final concentration, followed by incubation at 30 °C for 4 hours at 180 rpm. Afterwards, cells were harvested by centrifugation (4000 × g, 4 °C, 20 min), and then re-suspended in lysis buffer (50 mM HEPES, pH 7.5, 100 mM NaCl, 1 × Complete™ EDTA-free Protease Inhibitor Cocktail Tablet (Roche), 0.02 mg/ml DNaseI). The cells were lysed by sonication (Vibra-Cell™ Ultrasonic liquid processor) and the cell debris removed by centrifugation (20 000 × g, 4 °C, 30 min). Proteins in the supernatant were purified at 4 °C using an ÄKTA pure FPLC system (GE Healthcare). The supernatant was passed through a HisTrap™ HP column (5 ml, GE healthcare), pre-equilibrated with buffer A (50 mM Tris-HCl (pH 8.0), 500 mM NaCl, 20 mM imidazole). Unbound proteins were washed with five column volumes of buffer A, followed by elution with buffer B (50 mM Tris-HCl (pH 8), 500 mM NaCl, 500 mM imidazole). Further purification was achieved by gel filtration chromatography using a Superdex S200 16/600 column (GE Healthcare) eluted with 50 mM HEPES (pH 7.5) and 150 mM NaCl, at the flow rate of 1 ml/min. CDP comprising fractions were pulled together and concentrated using Amicon Ultra-15 Centrifugal Filter Units (30,000 MWCO). A recombinant plasmid (pET28a) containing laminarin phosphorylase (Pro_7066) was kindly supplied by Prozomix limited, which was transformed into *E. coli* BL21 (DE3) cells, and the transformant grown according to the literature.[2] Briefly, 1 L of transformant in LB medium containing appropriate antibiotic (kanamycin, 50 µg/ml) was incubated at 22 °C overnight at 180 rpm. Heterologous protein expression was induced by adding IPTG to a final concentration of 0.2 mM, and further incubated at 18 °C overnight at 180 rpm. Harvesting of cells and purifications were achieved as previously described for CDP. Concentration of both proteins was determined by NanoDrop™ spectrophotometer (Thermo Fisher Scientific, UK). Concentrated enzymes were divided into aliquots and stored at - 80°C until required.

3. Phosphorylase activity assay

Optimization of enzymatic reaction conditions and kinetic measurements were performed measuring the inorganic phosphate (Pi) released from the sugar-1-phosphate in the phosphorylase synthetic reaction. Released Pi was measured with a colorimetric assay modified by De Groeve *et al*.[3] Briefly, 25 µl of reaction mixture containing 200 mM sodium molybdate ($Na_2MoO_4 \cdot 2H_2O$) were added to 75 µl of a colour solution (0.24% [w/v] sodium ascorbate dissolved in 0.1 N HCl solution) and the reaction incubated at room temp. until colour developed. The colour reaction was stopped by adding 75 µl of stop solution (2% [w/v] sodium citrate tribasic dihydrate in 2% [v/v] acetic acid). Absorbance at 620 nm (A_{620}) was measured using NUNC 96 well plates on a BMG Labtech FLUOStar Omega microplate reader.

4. Optimization of enzymatic reactions for the synthesis of terminal galactoside oligosaccharides

Optimum pH and temperature for the synthetic enzymatic reactions of CDP and Pro_7066 with galactose-1-phosphate (Gal1P) were established carrying out the colorimetric assay measuring the inorganic phosphate (Pi) released in the reaction (see section above). The enzymatic reactions were set up in 100 µl of reaction

cocktail containing acceptor (8 mM, cellobiose (**1**) for Pro_7066 and laminaribiose (**4**) for CDP, respectively), donor (25 mM), enzymes (0.1 mg/ml) and buffer (50 mM) (all concentrations are final concentrations). Sodium citrate (pH 3 to 6), HEPES (pH 7 and 8) and Tris-HCl (pH 9) at 50 mM were screened for determining optimum pH of both phosphorylases. The enzymatic reactions were tested at three different temperatures (22, 37 and 55 °C) to establish the optimum temperature. All reactions were incubated over 24 h and released Pi was periodically measured by colorimetric assay (see Section 3). A phosphate standard curve was made using Na_2HPO_4 ranging from 0.5 to 2.5 mM for calculating released Pi from above reactions.

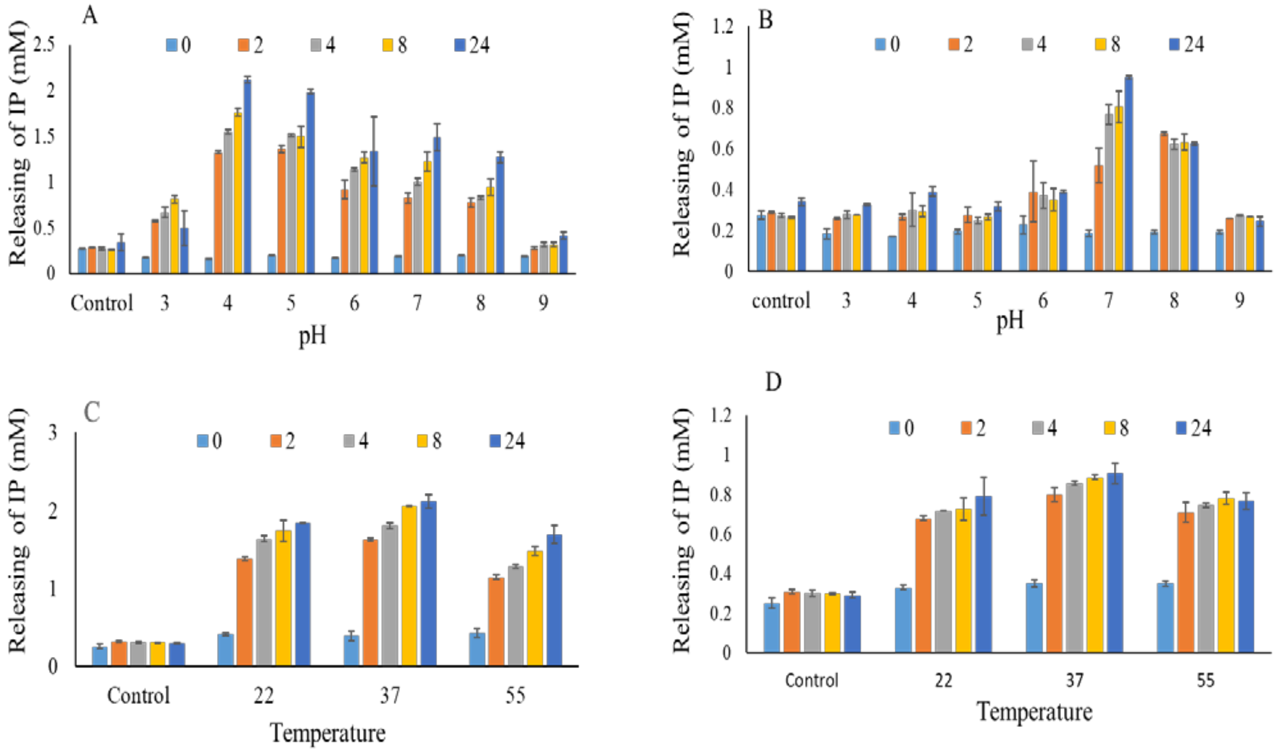

Figure S1. Effect of different pH and temperature on enzymes activities. (A) Effect of different pH on CDP, and (B) Pro_7066 activities. (C) Effect of different temperature on CDP, and (D) Pro_7066 activities. Reactions were performed in 100 µl of reaction cocktail containing acceptor (8 mM, cellobiose (**1**) for Pro_7066 and laminaribiose (**4**) for CDP, respectively), Gal1P (25 mM), enzymes (0.16 mg/ml) and buffer (50 mM). Sodium citrate (pH 3 to 6), HEPES (pH 7 and 8) and Tris-HCl (pH 9) at 50 mM were used for determining optimum pH (all concentrations are final concentrations). In control reactions, enzymes were heat killed prior addition into the reaction. Reactions were incubated up to 24 hours. IP = inorganic phosphate.

5. CDP-catalysed reactions of Gal1P with cognate acceptors 1 and 2

Reaction mixtures containing Gal1P (50 mM) as donor, cellobiose (**1**) or β-Glc-(1-4)-β-Glc-(1-3)-Glc (**2**) (12.5 mM) as acceptors and with CDP (0.125 mg/ml) in 25 mM sodium acetate buffer (pH 5) were incubated at 37 °C overnight with shaking at 300 rpm. The reactions were stopped by heating at 95 °C for 5 min. The products were purified by ion exchange chromatography to remove residual Gal1P, followed by gel filtration chromatography as described in Section 1.

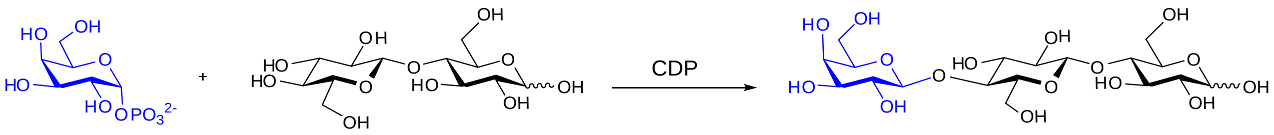

1 **9**

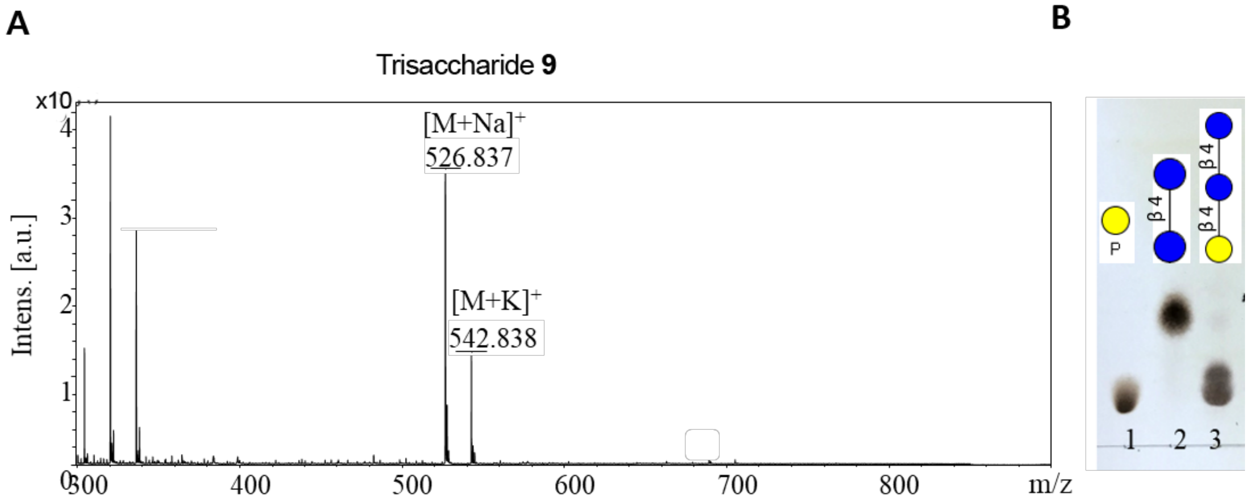

Figure S2 AB. (**A**) MALDI TOF MS of CDP-catalised reaction of cellobiose (**1**) with Gal1P after overnight incubation at 37 °C. (**B**) TLC analysis of of CDP-catalised reaction of **1** with Gal1P:lane 1 – Gal1P, lane 2 – cellobiose (**1**), lane 3 – reaction products. TLC mobile phase: isopropanol - NH_4OH - H_2O (6:3:1, v/v/v).

6. Pro_7066-catalysed reactions of Gal1P with cognate acceptors 3 and 4

The reactions were performed using Gal1P as donor and Glc-β-1-3-Glc-β-1-4-Glc (**3**) or laminaribiose (**4**) as acceptors in a ratio ranging from 16:1 to 1:2, with Pro_7066 at 0.125 mg/ml in 25 mM HEPES buffer (pH 7) (all concentrations are final concentrations). Reaction mixtures were incubated at 37 °C overnight with shaking at 300 rpm leading to the formation of a number of oligosaccharide products as judged by TLC analyses. To decrease a proportion of high-molecular weight oligosaccharides formed both reactions were performed using a 1:1 donor to acceptor ratio, incubated at 37 °C overnight and stopped by heating at 95 °C for 5 min. Reaction mixtures were initially purified by ion exchange chromatography for removal of residual Gal1P/Glc1P, and then freeze dried. The crude mixture (25 mg) of products was then incubated with β-1-3-glucanase (25 U/ml, Megazyme) in 50 mM sodium acetate buffer (1 ml, pH 5). Progress of these reactions was monitored by TLC and MALDI, as shown in Fig. S3. Finally, the crude mixtures were purified by gel filtration chromatography as described in Section 1.

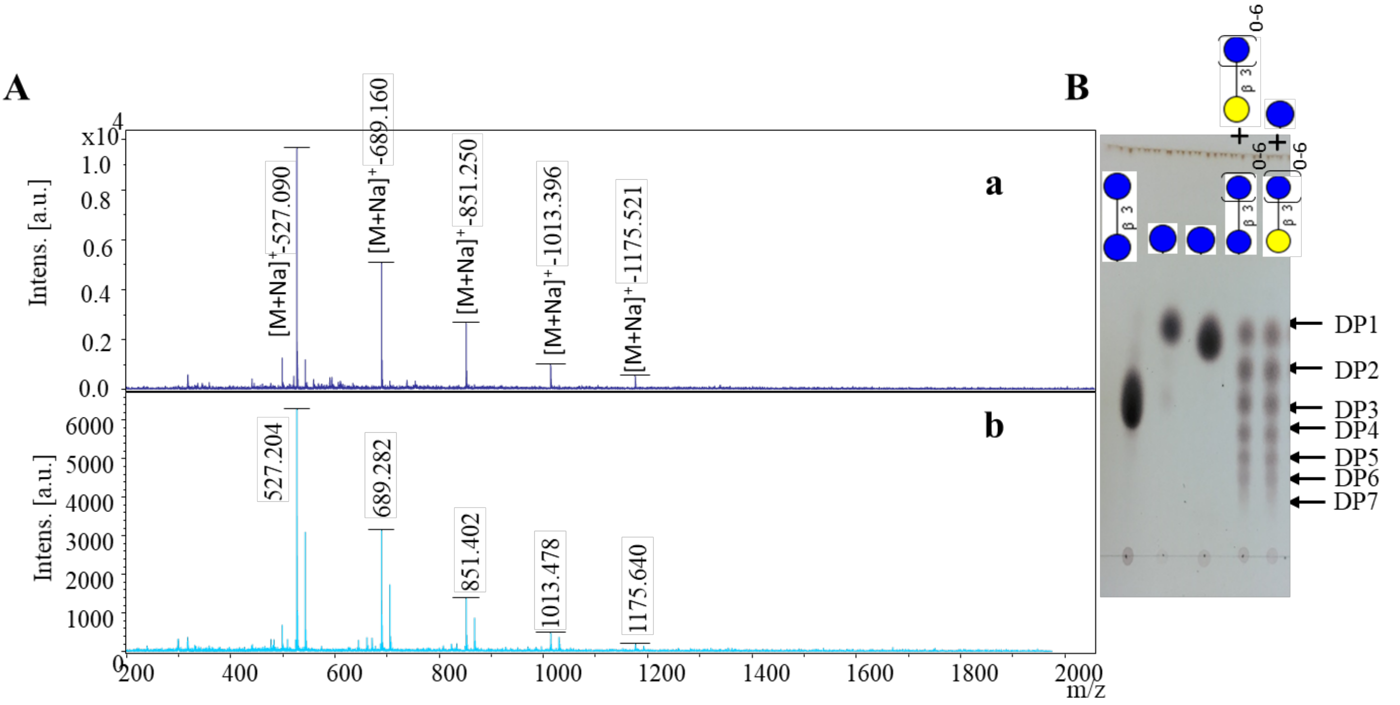

Figure S3 AB. (**A**) MALDI TOF MS of Pro_7066-catalised reaction of laminaribiose (**4**) (cognate acceptor) and Gal1P as donor. (a) Products after overnight incubation with Pro_7066 and (b) reaction mixture after additional incubation with exo-β-1-3-glucanase. **(B)** TLC analysis of Pro_7066-catalised reaction of laminaribiose (**4**) and Gal1P. Lane 1 – laminaribiose (**4**); lane 2 – glucose; lane 3 – overnight incubation of laminaribiose with exo-β-1-3-glucanase; lane 4 – reaction mixture after removal of residual Gal1P; lane 5 – reaction mixture after additional overnight incubation of with exo-beta-1-3-glucanase.

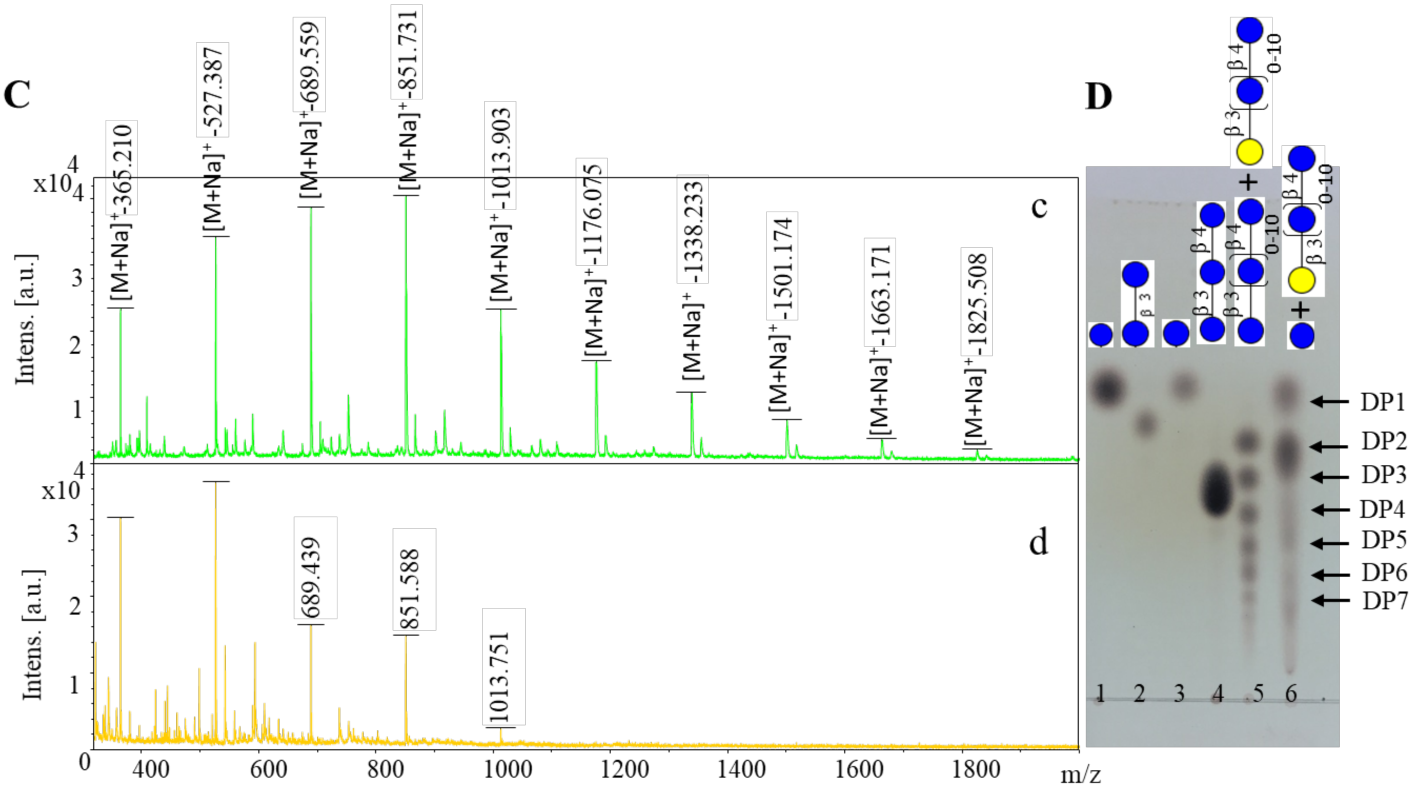

Figure S3 CD. (**C**) MALDI TOF MS of Pro_7066-catalised reaction of β-Glc-(1-3)-β-Glc-(1-4)-Glc (**3**) (cognate acceptor) and Gal1P as donor. (c) Products of overnight reaction with Pro_7066 and (d) reaction mixture after additional incubation with exo-β-1-3-glucanase. (**D**) TLC analysis of Pro_7066-catalised reaction of β-Glc-(1-3)-β-Glc-(1-4)-Glc (**3**) and Gal1P: Lane 1 – glucose, lane 2 – laminaribiose (**4**); lane 3 – overnight incubation of laminaribiose with exo-β-1-3-glucanase; lane 4 – trisaccharide **3**; lane 5 – reaction mixture after removal of residual Gal1P; lane 6 – reaction mixture after additional overnight incubation of with exo-beta-1-3-glucanase.

7. CDP and Pro_7066 reactions with non-cognate acceptors and donors

This general protocol describes reactions between Gal1P, ClcN1P and Man1P and a number of oligosaccharide acceptors as shown in Table 1 (main text). All reactions were performed using sugar 1-phosphate (25 mM), an acceptor (8 mM) and CDP (0.1 mg/ml) in 50 mM sodium citrate buffer (pH 5-6) or Pro_7066 (0.1 mg/ml) in 50 mM HEPES buffer (pH 7). Reactions were incubated at 37 °C with shaking at 300 rpm for 3 days keeping pH constant and adding extra amount of enzyme (10 µg) after 24 and 48 h. Reactions were stopped upon completion by heating at 95 °C for 5 min. Then, the reaction mixtures were first purified by ion exchange chromatography to remove residual sugar 1-phosphates, followed by gel filtration chromatography to isolate the final products as described in Section 1.

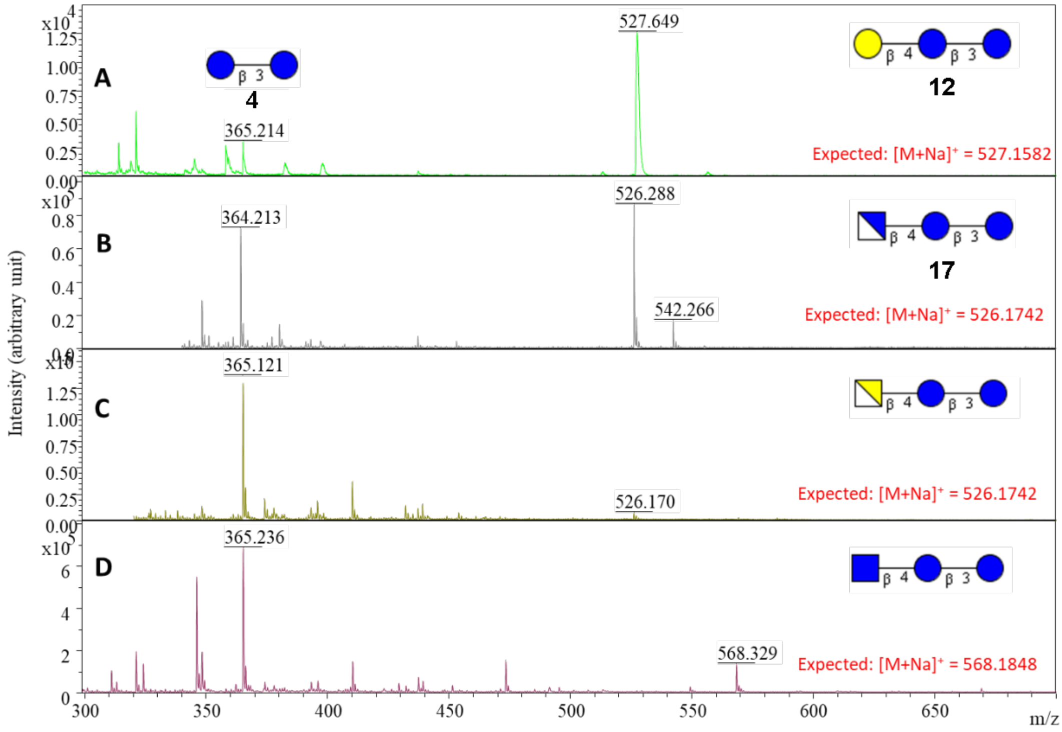

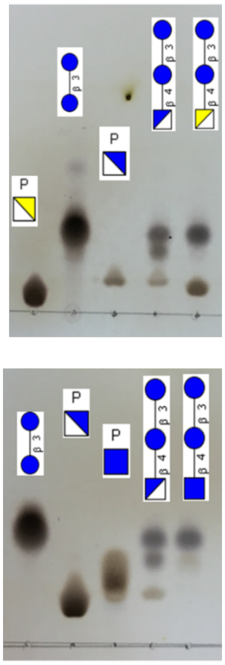

Figure S4. MALDI TOF MS and TLC for CDP reactions with laminaribiose (**4**) as non-cognate acceptor and Gal1P (A), GlcN1P (B), GalN1P (C), and GlcNAc-1-P (D) as donors. Typically, reactions were incubated for 3 days, and their progression was analysed by MALDI and TLC. Only reactions with Gal1P (A) and GlcN1P (B) showed good conversion, which allowed isolation of the final product. TLC mobile phase: isopropanol: NH_4OH:H_2O (6:3:1, v/v/v).

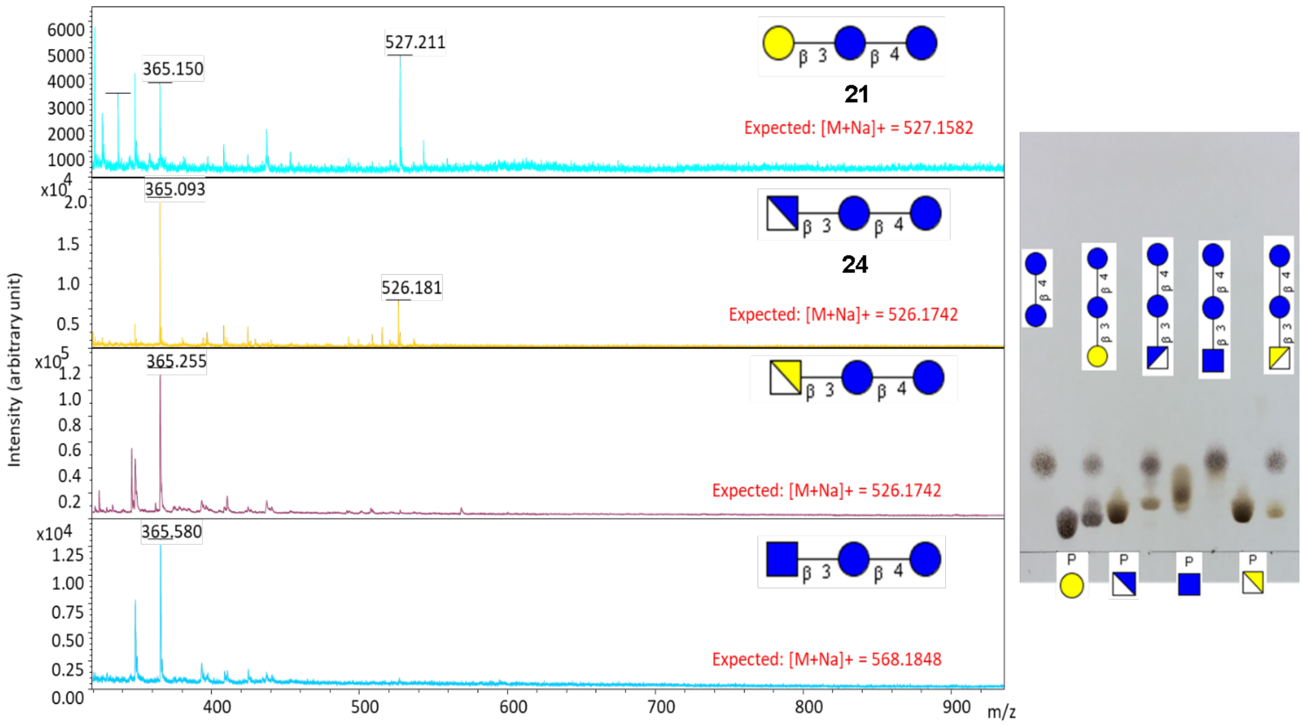

Figure S5. MALDI TOF MS and thin layer chromatography for Pro_7066 reactions with cellobiose (**1**) as non-cognate acceptor and Gal1P (A), GlcN1P (B), GalN1P (C), and GlcNAc-1-P (D) as donors. Typically, reactions were incubated for 3 days, and their progression was analysed by MALDI and TLC. Only reactions with Gal1P (A) and GlcN1P (B) showed good conversion, which allowed isolation of the final product. TLC mobile phase: isopropanol: NH_4OH:H_2O (6:3:1, v/v/v).

8. Influence of acceptor chain length on the enzymatic galactosylation of glucans

The impact of increasing degree of polymerization on efficiency of enzymatic galactosylation of glucooligosaccharide acceptors was evaluated in two series of experiments which aimed at measuring the released inorganic phosphate. In the first series, Gal1P (25 mM) and laminarioligosaccharide acceptors **4** - **6** (8 mM) were incubated with CDP (0.1 mg/ml) in 50 mM sodium acetate buffer (pH 5). In the second series of experiments Gal1P (25 mM) was incubated with cellooligosaccharides acceptors **1**, **3** and cellotetraose (8 mM) in the presence of Pro_7066 (0.1 mg/ml) in 50 mM HEPES (pH 7). All concentrations are final concentrations for reactions which were all carried out in the same way in a total volume of 100 µl each at 37 °C with shaking at 300 rpm for up to 2 hours concentrations are final concentrations). Concentration of the released phosphate was determined by colorimetric assay (see Section 3).

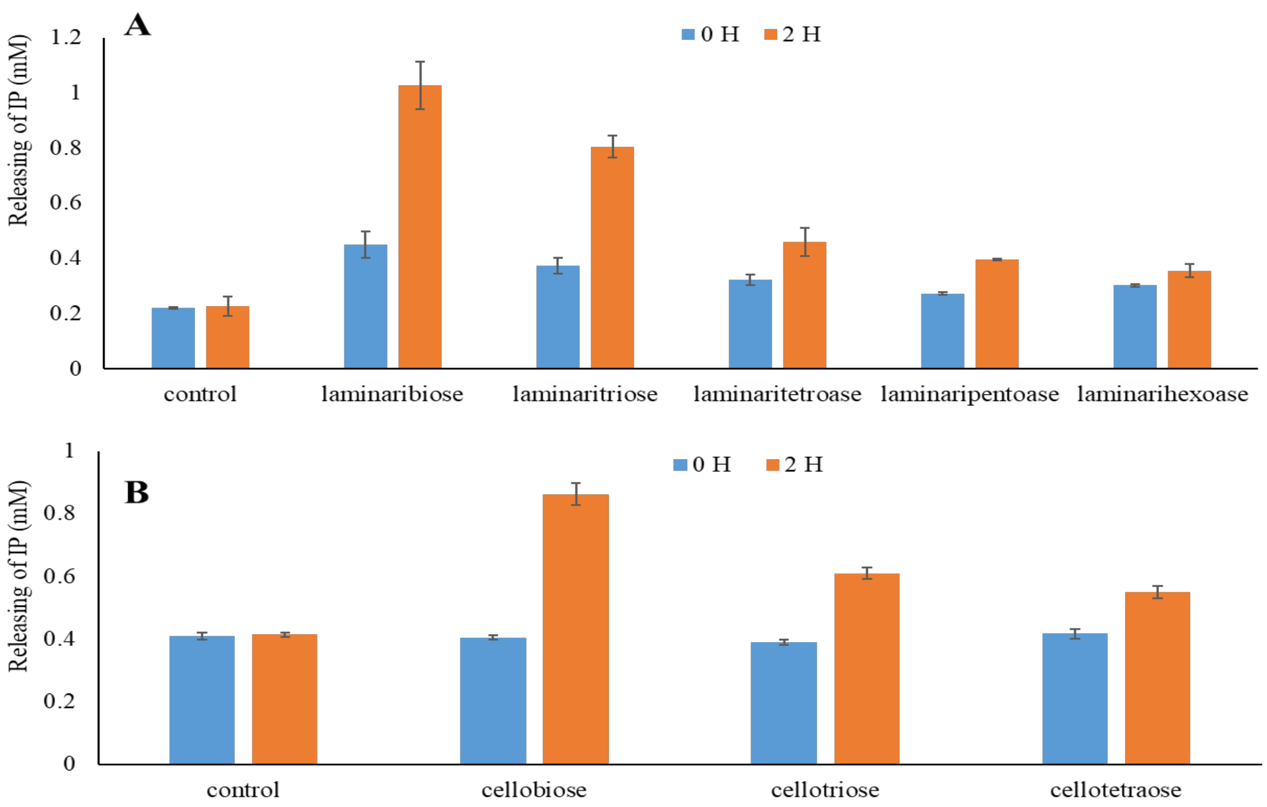

Figure S6. Inorganic phosphate release in galactosylation of glucooligosaccharides with Gal1P. (A) Enzyme - CDP, acceptors – laminarioligosaccharide **5** – **8**, buffer – NaOAc (pH 5); (B) enzyme – Pro_7066, acceptors – cellooligosaccharides **1**, **3** and cellotetraose, buffer - HEPES (pH 7). In all cases concentrations of enzyme - 10 μg/mL, Gal1P – 25 mM, acceptor – 8 mm in a total of 100 μl of 50 mM buffer. In control reactions, enzymes were heat killed prior addition into the reaction. Reactions were incubated up to 2 hours at 37 °C. P_i = inorganic phosphate.

9. Kinetic analysis of donor specificities in reactions catalysed by CDP and Pro_7066

Kinetic analyses were performed for Pro_7066 and CDP with the 4 different donors, natural Glc1P, and unnatural Gal1P, GlcN1P and Man1P, in combination with the corresponding cognate and non-cognate acceptors, laminaribiose and cellobiose, respectively. All kinetic analyses were performed in the synthetic direction, measuring release of inorganic phosphate from sugar-1-P donor. The concentration of released Pi was measured colorimetrically, as described in Section 3.

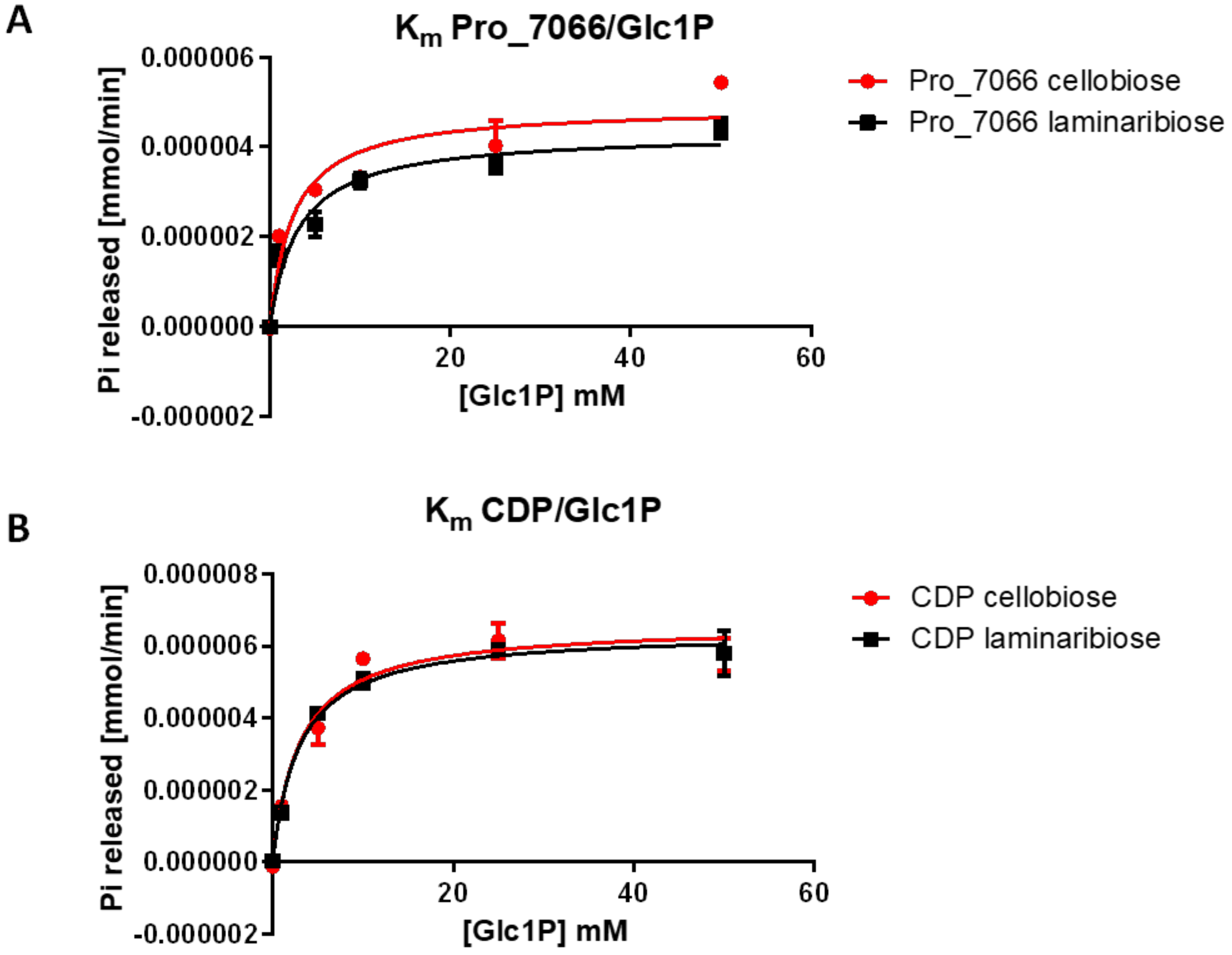

Figure S7. Michaelis-Menten plots for Glc1P as donor, and laminaribiose and cellobiose as acceptors for (A) Pro_7066 and (B) CDP. Glc1P was used at 0, 1, 5, 10, 25 and 50 mM with acceptor (5 mM) and Pro_7066/CDP at 0.01 mg/ml in 100 mM HEPES (pH 7.2) (all concentrations are final concentrations). Reactions were carried out at room temperature and sampled at 15 min. Samples were boiled at 95 °C for 5 min for deactivating enzymes. All the reactions were performed in the triplicates. Data were plotted using GraphPad Prism (v 5.04).

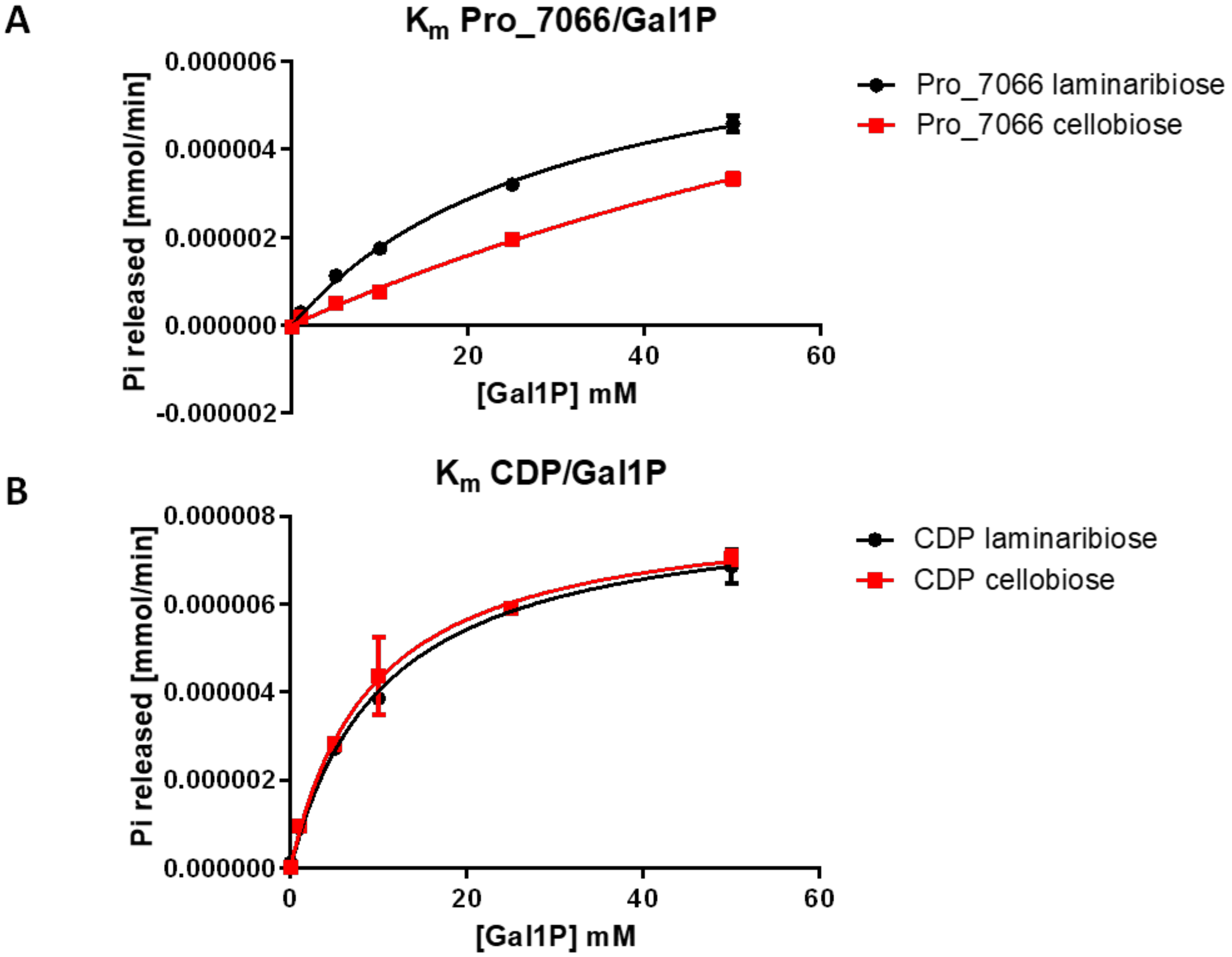

Figure S8. Michaelis-Menten plots for Gal1P as donor, and laminaribiose and cellobiose as acceptors for (A) Pro_7066 and (B) CDP. Gal1P was used at 0, 1, 5, 10, 25 and 50 mM with acceptor (5 mM) and Pro_7066/CDP at 0.35 mg/ml in 100 mM HEPES (pH 7.2) (all concentrations are final concentrations). Reactions were carried out at room temperature and sampled at 25 min. Samples were boiled at 95 °C for 5 min for deactivating enzymes. All the reactions were performed in the triplicates. Data were plotted using GraphPad Prism (v 5.04).

A

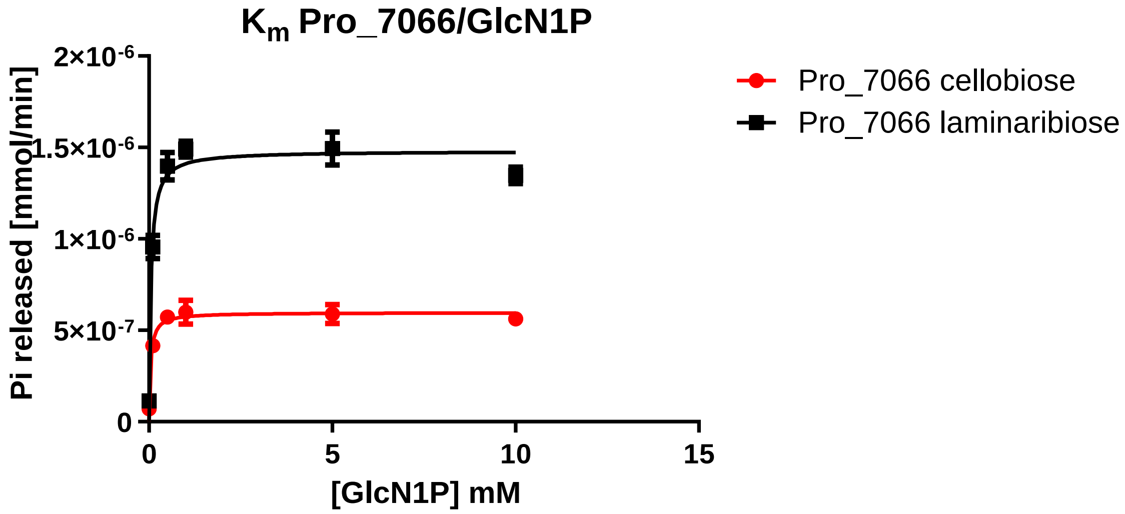

B

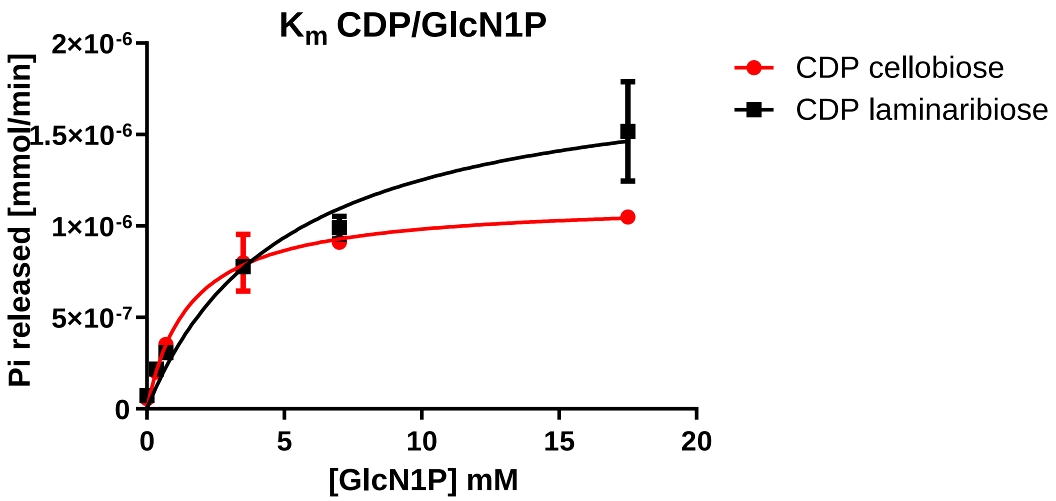

Figure S9. Michaelis-Menten plots for GlcN1P as donor, and laminaribiose and cellobiose as acceptors for (A) Pro_7066 and (B) CDP. (A) GlcN1P was used at 0, 0.1, 0.5, 1, 5 and 10 mM with acceptor (5 mM) and Pro_7066 at 0.35 mg/ml in 100 mM HEPES (pH 7.2) (all concentrations are final concentrations). Reactions were carried out at room temperature and sampled at 30 min. Samples were boiled at 95 °C for 5 min for deactivating enzymes. (B) GlcN1P was used at 0, 0.35, 0.7, 3.5, 7 and 17.5 mM with acceptor (5 mM) and CDP at 0.35 mg/ml in 100 mM HEPES (pH 7.2) (all concentrations are final concentrations). Reactions were carried out at room temperature and sampled at 25 min. Samples were boiled at 95 °C for 5 min for deactivating enzymes. All the reactions were performed in the triplicates. Data were plotted using GraphPad Prism (v 5.04).

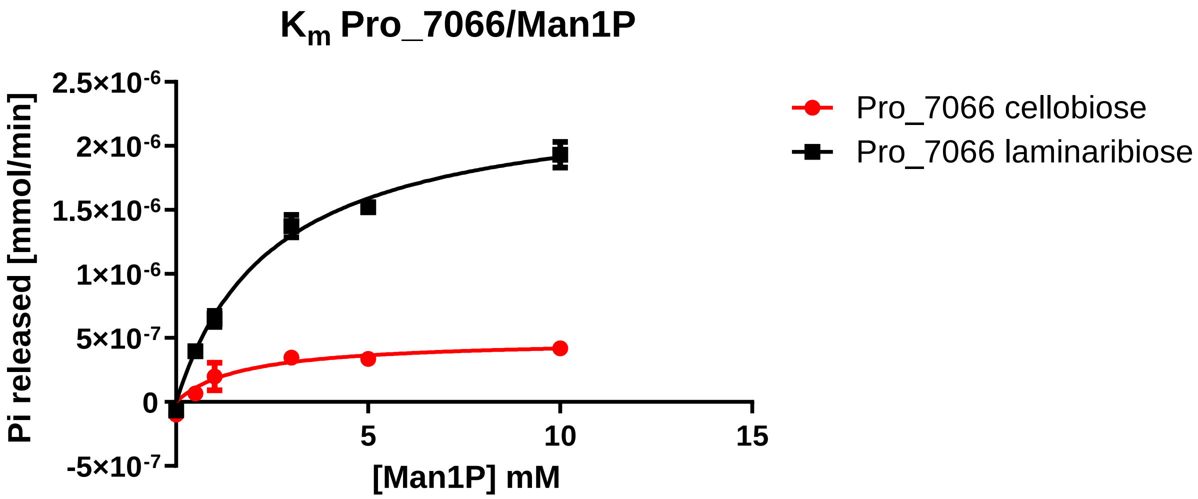

Figure S10. Michaelis-Menten plot for Man1P as donor, and laminaribiose and cellobiose as acceptors for Pro_7066. Man1P was used at 0, 0.5, 1, 3, 5 and 10 mM with acceptor (5 mM) and Pro_7066 at 0.35 mg/ml in 100 mM HEPES (pH 7.2) (all concentrations are final concentrations). Reactions were carried out at room temperature and sampled at 30 min. Samples were boiled at 95 °C for 5 min for deactivating enzymes. All the reactions were performed in the triplicates. Data were plotted using GraphPad Prism (v 5.04).

10. Chemical synthesis of sugar 1-phosphates

α-D-mannopyranose-1-phosphate bis(triethylammonium salt) (Man1P)

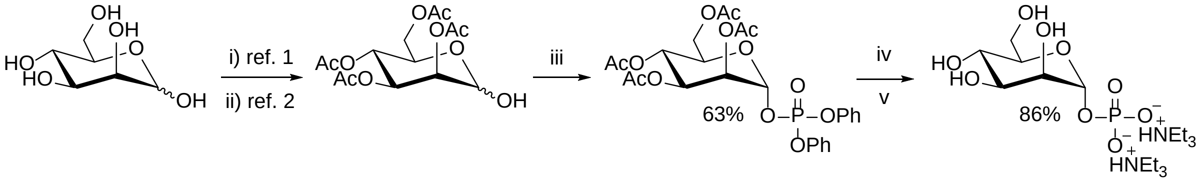

Scheme S1. Reagent and conditions: i) ref. 5; ii) ref. 6; iii) *n*-BuLi, $(PhO)_2POCl$, THF, -78 °C to room temp., overnight; iv) PtO_2, $H_{2(g)}$, EtOH, room temp., 24 h; v) Et_3N:H_2O:MeOH (1:3:7, v/v/v), room temp., 48 h.

n-BuLi (823 µL of 1.6 M in hexane; 1.31 mmol; 1.2 equiv) was added dropwise to the solution of 2,3,4,6-tetra-*O*-acetyl-D-mannose (380 mg; 1.09 mmol; 1 equiv) (synthesized from D-mannose as reported methods)[5,6] in anhydrous THF (15 mL) at -78 °C under N_2 atmosphere and stirred for 10-15 min. Diphenyl phosphoryl chloride (272 µL; 1.31 mmol; 1.2 equiv) was added dropwise to the mixture, which was allowed to warm to room temp. overnight. The reaction was quenched with NH_4Cl saturated solution (10 mL) and partitioned with EtOAc (3 x 15 mL).[7] The organic phase was washed with NaCl saturated solution (2 x 10 mL), dried over $MgSO_4$, filtered, concentrated under vacuum and purified by flash chromatography [cartridge SNAP 25g; solvent: Hexane/EtOAc; gradient: 0-20% and 30-30% (v/v); flow: 40 mL/min] to afford the product diphenyl (2,3,4,6-tetra-*O*-acetyl-α-D-mannopyranosyl) phosphate[8] in 63% yield (400 mg; 0.69 mmol). The sequential deprotection[7] of the synthesised product (80 mg; 138 µmol) was firstly carried out with PtO_2 (6.35 mg; 138 µmol; 0.2 equiv) in absolute ethanol (3 mL) at room temp. under H_2 atmosphere for 24 h. After catalyst removal by filtration, the crude was concentrated under vacuum, dissolved in MeOH (2 mL) followed by addition of

Et_3N (1 mL) and concentrated again. Secondly, the residue was dissolved in $Et_3N:H_2O:MeOH$ (1:3:7, v/v/v) (5 mL), stirred for 48 h at room temp. and concentrated under vacuum to afford α-D-mannopyranosyl phosphate (triethylammonium salt)[9] in 86% yield (60 mg; 119 µmol). ^1H NMR (600 MHz, D_2O) δ 5.24 (dd, $J_{1,P}$ = 8.0 Hz, $J_{1,2}$ = 2.0 Hz, 1H, H-1), 3.81 (dd, $J_{2,3}$ = 3.3 Hz, $J_{1,2}$ = 1.9 Hz,1H, H-2), 3.75 (dd, $J_{3,4}$ = 9.8 Hz, $J_{2,3}$ = 3.4 Hz, 1H, H-3), 3.72 (dd, $J_{6,6'}$ = 12.1 Hz, $J_{5,6}$ = 2.1 Hz, 1H, H-6), 3.68 (ddd, $J_{4,5}$ = 10.0 Hz, $J_{5,6'}$ = 5.7 Hz, $J_{5,6}$ = 2.1 Hz, 1H, H-5), 3.60 (dd, $J_{6,6'}$ = 12.1 Hz, $J_{5,6'}$ = 5.7 Hz, 1H, H-6'), 3.51 (t, $J_{3,4}$ = $J_{4,5}$ = 9.9 Hz, 1H, H-4), 3.04 (q, J = 7.3 Hz, 12H, 2x HN(C$\underline{H}_2$CH$_3$)$_3$), 1.12 (t, J = 7.3 Hz, 18H, 2x HN(CH$_2$C$\underline{H}_3$)$_3$). ^{13}C NMR (151 MHz, D_2O): δ 95.64 (C-1), 73.39 (C-5), 70.62 (C-2), 69.91 (C-3), 66.58 (C-4), 60.89 (C-6), 46.61 (HN($\underline{C}$H$_2$CH$_3$)$_3$), 8.21 (HN(CH$_2\underline{C}$H$_3$)$_3$). ^{31}P NMR (162 MHz, D_2O): δ -1.47. HRMS (ESI-TOF): *m/z* calculated for $C_6H_{12}O_9P$ [M-H]$^-$: 259.0219; found 259.0218.

2-Amino-2-deoxy-α-D-glucopyranose-1-phosphate disodium salt (GlcN1P)

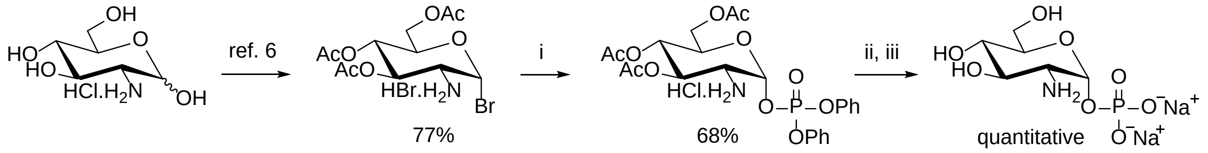

Scheme S2. Reagent and conditions: i) a) Et_3N, $(PhO)_2POOH$, toluene, room temp., 5 h; b) 2N HCl; ii) PtO_2, H_2, MeOH, room temp., overnight; iii) $NaOCH_3$, MeOH, room temp., 24 h.

Et_3N (163 µL; 1.18 mmol; 1 equiv) was added dropwise to a solution of 3,4,6-tri-*O*-acetyl-2-amino-2-deoxy-α-D-glucopyranosyl bromide hydrobromide (530 mg; 1.18 mmol; 1 equiv) (synthesized from D-glucosamine hydrochloride as reported method)[10] in anhydrous toluene (8 mL) at room temp. under N_2 atmosphere and stirred for 20 min. A solution of diphenyl phosphate (295 mg; 1.18 mmol; 1 equiv) in anhydrous toluene (2 mL), also treated with Et_3N (163 µL; 1.18 mmol; 1 equiv) in the same conditions as mentioned for the sugar, was added dropwise to the mixture and stirred at room temp. for 5h. The reaction was filtered and the filtrate was concentrated under vacuum. The crude was cooled with ice bath, acidified with 2N HCl solution (4 mL) under stirring and stored overnight at 4 °C. The precipitate was washed several times with Et_2O to afford the compound diphenyl (3,4,6-tri-*O*-acetyl-2-amino-2-deoxy-α-D-glucopyranosyl) phosphate hydrochloride[11] in 68% yield (460 mg; 0.80 mmol; α/β 20:1), which was used in the next step without further purification. The hydrogenolysis of the synthesised compound (60 mg; 104 µmol; 1 equiv) was performed with PtO_2 (12 mg; 52 µmol; 0.5 equiv) in anhydrous methanol (2 mL) under H_2 atmosphere at room temp. overnight. After catalyst removal by filtration, the filtrate was cooled with ice bath and 0.5 M $NaOCH_3$ solution (600 µL) was added. The reaction was stirred at room temp. for 24 h, quenched with 0.1N HCl solution (pH 7-8) and concentrated under vacuum. The syrup was dissolved in water and freeze dried to afford title compound[12] in quantitative yield (32 mg; 104 µmol). ^1H NMR (600 MHz, D_2O) δ 5.67 (dd, $J_{1,P}$ = 7.3 Hz, $J_{1,2}$ = 3.4 Hz, 1H, H-1), 4.00-3.94 (m, 2H, H-3, H-5), 3.91 (dd, $J_{6,6'}$ = 12.4 Hz, $J_{5,6}$ = 2.3 Hz, 1H, H-6), 3.81 (dd, $J_{6,6'}$ = 12.4 Hz, $J_{5,6'}$ = 4.8 Hz, 1H, H-6'), 3.52 (dd, $J_{4,5}$ = 10.1 Hz, $J_{3,4}$ = 9.2 Hz, 1H, H-4), 3.34 (ddd, $J_{2,3}$ = 10.5 Hz, $J_{1,2}$ = 3.4 Hz, $J_{2,P}$ = 1.6 Hz, 1H, H-2). ^{13}C NMR (151 MHz, D_2O) δ 90.90 (C-1), 72.30 (C-5), 69.83 (C-3), 69.56 (C-4), 60.32 (C-6), 54.67 (C-2). ^{31}P NMR (162 MHz, D_2O) δ –1.77. HRMS (ESI-TOF) *m/z* calculated for $C_6H_{13}NO_8P$ [M-H]$^-$: 258.0379; found 258.0378.

11. Charactrisaction of oligosaccharides synthesised by enzymatic glycosylation with CDP and Pro_7066

β-D-Gal^{iii}-(1→4)-β-D-Glc^{ii}-(1→4)-D-Glc^{i} (9)

Compound **9** was prepared from **Gal1P** and cellobiose **1** according to the procedure described in Section 5. Yield 95%. ^{1}H NMR (600 MHz, D_2O) δ 5.16 (d, *J* = 3.8 Hz, 0.53 H, H-1^{i} α), 4.59 (d, *J* = 7.9 Hz, 0.47 H, H-1^{i} β), 4.47 (d, *J* = 7.9 Hz, 1 H-1^{ii} β), 4.38 (d, *J* = 7.8 Hz, 1 H, H-1^{iii} β), 3.94 – 3.84 (m, 6H), 3.82 – 3.46 (m, 24H), 3.32 – 3.28 (m, 1H), 3.21 (t, *J* = 8.5 Hz, 0.47 H, H-2^{i} β). ^{13}C NMR (151 MHz, D_2O) δ 102.92 (C-1^{iii} β), 102.34 (C-1^{ii} β), 95.76 (C-1^{i} β), 91.82 (C-1^{i} α), **78.63, 78.48, 78.10 (C-4^{i}α,β, C-4^{ii})**, 75.35, 74.82, 74.79, 74.24, 74.13, 73.79 (C-2^{i} β), 72.87 (C-2^{ii} β), 72.59 (C-3^{iii} β), 71.30, 71.22, 70.94 (C-2^{iii} β), 70.11, **68.44 (C-4^{iii} β)**, 61.02, 59.95, 59.88. HRMS (ESI-TOF) *m/z* calculated for $C_{18}H_{32}O_{16}Na$ $[M+Na]^+$: 527.1583, found 527.1771.

β-D-Gal^{iv}-(1→4)-β-D-Glc^{iii}-(1→4)-β-D-Glc^{ii}-(1→3)-D-Glc^{i} (10)

Compound **10** was prepared from **Gal1P** and trisaccharide **2** according to the procedure described in Section 5. Yield 70%. ^{1}H NMR (400 MHz, D_2O) δ 5.10 (d, *J* = 3.8 Hz, 0.6 H, H-1^{i} α), 4.64 (overlaps with HOD, H-1^{ii} β), 4.54 (d, *J* = 8.0 Hz, 0.4 H, H-1^{i} β), 4.40 (d, *J* = 8.0 Hz, 1 H, H-1^{iii} β), 4.32 (d, *J* = 7.8 Hz, 1 H, H-1^{iv} β), 3.89 – 3.46 (m, 29 H), 3.44 – 3.20 (m, 7 H). ^{13}C NMR (101 MHz, D_2O) δ 102.81 (C-1^{iv} β), 102.51 (C-1^{ii} β), 102.27 (C-1^{iii} β), 95.61 (C-1^{i} β), 91.93 (C-1^{i} α), 84.34 (C-3^{i} β), 82.10 (C-3^{i} α), 78.29 (C-4^{ii} β), **77.94 (C-4^{iii} β),** 75.47, 75.27, 74.74 (C-5^{iv} β), 74.01, 73.74, 73.17, 72.75, 72.40 (C-3^{iv} β), 71.10, 70.85 (C-2^{iv} β), 68.24 (C-4^{iv} β), 67.96 (C-4^{i} β), 67.96 (C-4^{i} α), 60.97, 60.60, 60.41, 60.39, 59.76. HRMS (ESI-TOF) *m/z* calculated for $C_{24}H_{42}O_{21}Na$ $[M+Na]^+$: 689.2116 and found 689.2250.

β-D-Gal^{iv}-(1→4)-β-D-Glc^{iii}-(1→3)-β-D-Glc^{ii}-(1→4)-D-Glc^{i} (11)

Compound **11** was prepared from **Gal1P** and trisaccharide **3** according to the procedure described in Section 7. Yield 60%. ^{1}H NMR (400 MHz, D_2O) δ 5.15 (d, *J* = 3.7 Hz, 0.6 H, H-1^{i} α), 4.58 (d, *J* = 8.0 Hz, 0.4 H, H-1^{i} β), 4.46 (d, *J* = 8.0 Hz, 1 H, H-1^{ii} β), 4.37 (d, *J* = 7.8 Hz, 1 H, H-1^{iv} β), 3.94 – 3.82 (m, 6 H), 3.81 – 3.76 (m, 2 H), 3.77 – 3.40 (m, 24 H), 3.34 (dd, *J* = 10.1, 5.9 Hz, 1 H, H-2^{iii} β), 3.20 (dd, *J* = 9.1, 7.9 Hz, 1 H, H-1^{i} β). ^{13}C NMR (101 MHz, D_2O) δ 102.93 (C-1^{iv} β), 102.53 (C-1^{iii} β), 102.29 (C-1^{ii} β), 95.75 (C-1^{i} β), 91.78 (C-1^{i} α), 83.79 (C-3^{ii} β), 78.70, 78.56, **78.27 (C-4^{iii} β)**, 75.57, 75.34, 74.76, 74.27, 74.17 (C-3^{iii} β), 73.87, 73.12, 73.00, 72.49 (C-3^{iv} β), 71.20, 70.92 (C-2^{iv} β), 70.08, 68.55 (C-4^{iv} β), 67.93 (C-4^{ii} β), 61.03, 60.53, 60.02. HRMS (ESI-TOF) *m/z* calculated for $C_{24}H_{42}O_{21}Na$ $[M+Na]^+$: 689.2110 found 689.2208.

β-D-Gal^{iii}-(1→4)-β-D-Glc^{ii}-(1→3)-D-Glc^{i} (12)

Compound **12** was prepared from **Gal1P** and laminaribiose **4** according to the procedure described in Section 7. Yield 81%. ^{1}H NMR (400 MHz, D_2O) δ 5.18 (d, *J* = 3.7 Hz, 0.6 H, H-1^{i} α), 4.71 (d, *J* = 7.7 Hz, 0.4 H, H-1^{ii} β), 4.62 (d, *J* = 8.0 Hz, 0.4 H-1^{i} β), 4.39 (d, *J* = 7.8 Hz, 1 H, H-1^{iii} β), 3.96 – 3.32 (m, 26 H). ^{13}C NMR (101 MHz, D_2O) δ 102.94 (C-1^{iii} β), 102.69 (C-1^{ii} β), 102.60 (C-1^{ii} α), 95.68 (C-1^{i} β), 92.03 (C-1^{i} α), 84.52 (C-3^{i} β), 82.26 (C-3^{i} α), **78.29 (C-4^{ii} β)**, 75.57, 75.36, 74.83 (C-5^{iii} β), 74.21 (C-3^{ii} β), 73.82, 73.20, 73.17 (C-2^{ii} β), 72.51 (C-3^{iii} β), 71.22, 71.04, 70.96 (C-2^{iii} β), 68.57 (C-4^{iii} β), 68.12, 68.08, 61.05, 60.73, 60.57, 60.06. HRMS (ESI-TOF) *m/z* calculated for $C_{18}H_{32}O_{16}Na$ $[M+Na]^+$: 527.1583 found 527.1500.

β-D-Gal^{iv}-(1→4)-β-D-Glc^{iii}-(1→3)-β-D-Glc^{ii}-(1→3)-D-Glc^{i} (13)

Compound **13** was prepared from **Gal1P** and laminaritriose **5** according to the procedure described in Section 7. Yield 60%. ^{1}H NMR (600 MHz, D_2O) δ 5.08 (d, *J* = 3.7 Hz, 0.6 H, H-1^{i} α), 4.51 (d, *J* = 8.0 Hz, 0.4 H, H-1^{i} β), 4.29 (d, *J* = 7.8 Hz, 1 H, H-1^{iv} β), 3.85 – 3.44 (m, 26 H), 3.43 – 3.23 (m, 11 H), 3.22 – 3.18 (m, 0.4 H). ^{13}C NMR (151 MHz, D_2O) δ 102.93 (C-1^{iv} β), 102.63 (C-1^{iii} β), 102.56 (C-1^{ii} β), 102.53 (C-1^{ii} α), 95.68 (C-1^{i} β), 92.01 (C-1^{i} α), 84.47 (C-3^{i} β), 84.10 (C-3^{ii} β), 82.24 (C-3^{i} α), **78.27 (C-4^{iii} β)**, 75.60, 75.57, 75.35, 74.83 (C-5^{iv} β), 74.19 (C-3^{iii} β), 73.82, 73.28, 73.25 73.15 (C-2^{ii} β, C-2^{iii} β), 72.50 (C-3^{iv} β), 71.21, 71.05, 70.93 (C-2^{iv} β), 68.54 (C-4^{iv} β), 68.11, 61.02, 60.69, 60.52, 60.04. HRMS (ESI-TOF) *m/z* calculated for $C_{24}H_{42}O_{21}Na$ [M+Na]$^{+}$: 689.2110 found 689.2208.

β-D-Gal^{v}-(1→4)-β-D-Glc^{iv}-(1→3)-β-D-Glc^{iii}-(1→3)-β-D-Glc^{ii}-(1→3)-D-Glc^{i} (14)

Compound **14** was prepared from **Gal1P** and laminaritetraose **6** according to the procedure described in Section 7. Yield 31%. ^{1}H NMR (600 MHz, D_2O) δ 5.08 (d, *J* = 3.7 Hz, 0.63, H, H-1^{i} α), 4.51 (d, *J* = 8.0 Hz, 0.37 H, H-1^{i} β), 4.29 (d, *J* = 7.8 Hz, 1 H, H-1^{v} β), 3.85 – 3.80 (m, 2H), 3.77 (m, 6 H), 3.73 – 3.45 (m, 21 H), 3.44 – 3.21 (m, 14 H). ^{13}C NMR (151 MHz, D_2O) δ 102.93 (C-1^{v} β,), 102.63 (C-1^{iv} β), 102.53 (C-1^{iii} β, C-1^{ii} β), 95.68 (C-1^{i} β), 92.01 (C-1^{i} α), 84.47 (C-3^{i} β), 84.06 (C-3^{ii} β, C-3^{iii} β), 82.24 (C-3^{i} α), 81.87, **78.27 (C-4^{iv} β)**, 75.61, 75.35, 74.83 (C-5^{v} β), 74.19 (C-3^{iv} β), 73.83, 73.26, 73.15, 72.50 (C-3^{v} β), 71.47, 71.21, 70.93 (C-2^{v} β), 70.07, 68.54 (C-4^{v} β), 68.07 (C-4^{iii} β, C-4^{ii} β , C-4^{i} β), 67.71 (C-4^{i} α), 60.69, 60.52, 60.49, 60.14, 60.04. HRMS (ESI-TOF) *m/z* calculated for $C_{30}H_{52}O_{26}Na$ [M+Na]$^{+}$: 851.2638 found 851.2647.

β-D-Gal^{vi}-(1→4)-β-D-Glc^{v}-(1→3)-β-D-Glc^{iv}-(1→3)-β-D-Glc^{iii}-(1→3)-β-D-Glc^{ii}-(1→3)-D-Glc^{i} (15)

Compound **15** was prepared from **Gal1P** and laminaripentaose **7** according to the procedure described in Section 7. Yield 19%. ^{1}H NMR (600 MHz, D_2O) δ 5.08 (d, *J* = 3.7 Hz, 0.67 H, H-1^{i} α), 4.52 (d, *J* = 8.0 Hz, 0.33 H, H-1^{i} β), 4.29 (d, *J* = 7.8 Hz, 1 H, H-1^{vi} β), 3.83 (dd, *J* = 12.3, 2.2 Hz, 2 H), 3.80 – 3.75 (m, 8 H), 3.67 – 3.55 (m, 22 H), 3.54 – 3.49 (m, 5 H), 3.42 – 3.33 (m, 21 H), 3.29 – 3.23 (m, 2 H). ^{13}C NMR (151 MHz, D_2O) δ 102.93 (C-1^{vi} β), 102.63 (C-1^{v} β), 102.56 (C-1^{iv} β, C-1^{iii} β, C-1^{ii} β), 95.68 (C-1^{i} β), 92.01 (C-1^{i} α), 84.47 (C-3^{i} β), 84.11 (C-3^{iv} β, C-3^{iii} β, C-3^{ii} β), 82.24 (C-3^{i} α), **78.28 (C-4^{v} β)**, 75.61, 75.35, 74.83 (C-5^{vi} β), 74.20 (C-3^{v} β), 73.83, 73.29, 73.15, 72.50 (C-3^{vi} β), 71.21, 71.05, 70.93 (C-2^{vi} β), 68.54 (C-4^{vi} β), 68.07 (C-4^{iv} β, C-4^{iii} β, C-4^{ii} β), 61.02, 60.65, 60.52, 60.04. HRMS (ESI-TOF) *m/z* calculated for $C_{36}H_{62}O_{31}Na$ [M+Na]$^{+}$: 1013.3173 found 1013.3138.

β-D-Gal^{vii}-(1→4)-β-D-Glc^{vi}-(1→3)-β-D-Glc^{v}-(1→3)-β-D-Glc^{iv}-(1→3)-β-D-Glc^{iii}-(1→3)-β-D-Glc^{ii}-(1→3)-D-Glc^{i} (16)

Compound **16** was prepared from **Gal1P** and laminarihexaose **8** according to the procedure described in Section 7. Yield 16%. ^{1}H NMR (600 MHz, D_2O) δ 5.08 (d, *J* = 3.7 Hz, 0.6 H, H-1^{i} α), 4.52 (d, *J* = 8.0 Hz, 0 .4 H, H-1^{i} β), 4.29 (d, *J* = 7.8 Hz, 1 H, H-1^{vii} β), 3.85 – 3.21 (m, 90 H). ^{13}C NMR (151 MHz, D_2O) δ 102.94 (C-1^{vii} β), 102.54 (C-1^{vi} β, C-1^{v} β, C-1^{iv} β, C-1^{iii} β, C-1^{ii} β), 95.73 (C-1^{i} β), 92.04 (C-1^{i} α), 84.50 (C-3^{i} β), 84.08 (C-3^{v} β, C-3^{iv} β. C-3^{iii}, C-3^{ii} β), 82.26 (C-3^{i} α), **78.28 (C-4^{vi} β)**, 75.62, 75.36, 74.84 (C-5^{Vii} β), 74.21 (C-3^{vi} β), 73.86, 73.31 (C-3^{vii} β), 73.17, 72.52 (C-3^{vii} β), 71.22, 71.07, 70.94 (C-2^{vii} β), 68.55 (C-4^{vii} β), 68.08 (C-4^{vi} β, C-4^{v} β, C-4^{iv} β, C-4^{iii} β, C-4^{ii} β, C-4^{i} β), 61.02, 60.67, 60.53, 60.05. HRMS (ESI-TOF) *m/z* calculated for $C_{42}H_{72}O_{36}Na$ [M+Na]$^{+}$: 1175.3701, found 1175.3850.

β-D-GlcN^{iii}-(1→4)-β-D-Glc^{ii}-(1→3)-D-Glc^{i} (17)

Compound **17** was prepared from **GlcN1P** and laminaribiose **4** according to the procedure described in Section 7. Yield 82%. ^{1}H NMR (600 MHz, D_2O) δ 5.07 (d, *J* = 3.7 Hz, 0.5 H, H-1^{i}α), 4.51 (d, *J* = 8.0 Hz, 0.5 H, H-1^{i}β), 4.42 (d, *J* = 8.3 Hz, 1H, H-1^{iii}β), 3.80 – 3.65 (m, 8H), 3.64 – 340 (m, 13H), 3.40 – 3.23 (m, 11H), 2.52 (t, *J* = 8.8 Hz, 2H, H-2^{iii}β). ^{13}C NMR (151 MHz, D_2O) δ 102.65 (C-1^{iii}β), 102.55 (C-1^{ii}β), 95.68 (C-1^{i}β), 92.02 (C-1^{i}α), 84.45 (C-3^{i}β), 82.20 (C-3^{i}α), **77.60 (C-4^{ii} β)**, 76.21, 75.56, 74.63, 73.94, 73.81, 73.31, 71.20, 71.03 (C-2^{i}α), 69.55 (C-3^{iii}β), 68.06, 60.70, 60.51, 60.24, **56.40 (C-2^{iii}β)**. HRMS (ESI-TOF) *m/z* calculated for $C_{18}H_{33}NO_{15}Na$ [M+Na]$^+$: 526.1748, found 526.1832.

β-D-Gal^{iii}-(1→3)-β-D-Glc^{ii}-(1→3)-D-Glc^{i} (19)

Compound **19** was prepared from **Gal1P** and laminaribiose **4** according to the procedure described in Section 6. Yield 38%. ^{1}H NMR (600 MHz, D_2O) δ 5.08 (d, *J* = 3.8 Hz, 0.65 H, H-1^{i}α), 4.52 (d, *J* = 8.0, Hz, 0.35 H, H-1^{i}β), 4.50 (d, *J* = 7.7 Hz, 1 H, H-1^{i}β), 4.48 (d, *J* = 7.8 Hz, 1 H, H-1^{iii}β), 3.79 – 3.72 (m, 9 H), 3.69 – 3.51 (m, 18 H), 3.46 – 3.32 (m, 5 H), 3.28 – 3.25 (dd, *J* = 9.3, 8.0 Hz, 1 H, H-2^{i}β). ^{13}C NMR (151 MHz, D_2O) δ 103.41 (C-1^{iii}β), 103.33 (C-1^{ii}β), 95.67 (C-1^{i}β), 92.02 (C-1^{i}α), **84.74- 84.70 (C-3^{ii}β and C-3^{i}β)**, 82.45 (C-3^{ii}α), 75.58, 75.50, 75.33, 73.71, 73.65, 72.57, 71.23, 71.14, 70.92, 68.58 (C-4^{iii} β), 68.25, 68.21, 61.05, 60.73, 60.55. HRMS (ESI-TOF) *m/z* calculated for $C_{18}H_{33}O_{16}Na$ [M+Na]$^+$: 527.1583 and found 527.1613.

β-D-Gal^{iv}-(1→3)-β-D-Glc^{iii}-(1→3)-β-D-Glc^{ii}-(1→4)-D-Glc^{i} (20)

Compound **20** was prepared from **Gal1P** and trisaccharide **3** according to the procedure described in Section 6. Yield 38%. ^{1}H NMR (600 MHz, D_2O) δ 5.07 (d, *J* = 3.9 Hz, 0.65 H, H-1^{i}α), 4.51 (d, *J* = 7.8 Hz, 1 H, H-1^{iv}β), 4.50 (d, *J* = 8.1 Hz, 0.35 H, H-1^{i}β), 4.39 (d, *J* = 8.0 Hz, 1 H, H-1^{ii}β), 3.83 – 3.23 (m, 33H), 3.11 (dd, *J* = 8.0, 7.3 Hz, 1 H). ^{13}C NMR (151 MHz, D_2O) δ 103.32 (C-1^{iv}β), 102.72 (C-1^{iii}β), 102.46 (C-1^{ii}β), 95.76 (C-1^{i}β), 91.82 (C-1^{i}α), 84.68 (C-3^{ii}β), 84.25 (C-3^{ii}α), **83.77 (C-3^{iii}β)**, 78.72 (C-4^{i}β), 78.57 (C-4^{i}α), 75.86, 75.54, 75.33, 74.78, 74.29, 73.89, 73.16, 73.05, 71.33, 71.23, 70.10, 69.58, 68.58 (C-4^{iv}β), 68.23, 67.95, 61.06, 60.74, 60.55, 60.04, 59.91. HRMS (ESI-TOF) *m/z* calculated for $C_{24}H_{42}O_{21}Na$ [M+Na]$^+$: 689.2116, found 689.2126.

β-D-Gal^{iii}-(1→3)-β-D-Glc^{ii}-(1→4)-D-Glc^{i} (21)

Compound **21** was prepared from **Gal1P** and cellobiose **1** according to the procedure described in Section 7. Yield 77%. ^{1}H NMR (600 MHz, D_2O) δ 5.07 (d, *J* = 3.8 Hz, 0.5 H, H-1^{i}α), 4.52 (d, *J* = 8.0 Hz, 1 H, H-1^{iii}β), 4.51 (d, *J* = 6.2 Hz, 1 H-1^{i}β), 4.39 (d, *J* = 8.0 Hz, 1 H, H-1^{ii}β), 3.82 – 3.73 (m, 6 H), 3.73 – 3.68 (m, 1 H), 3.67 – 3.55 (m, 12 H), 3.54 – 3.34 (m, 15 H), 3.13 (pt, *J* = 8.5 Hz, 1 H, H-2^{i}β). ^{13}C NMR (151 MHz, D_2O) δ 103.27 (C-1^{iii}β), 102.29 (C-1^{ii}β), 95.74 (C-1^{i}β), 91.79 (C-1^{i}α), **83.97 (C-3^{ii}β)**, 78.71 (C-4^{i}α) 78.57 (C-4^{i}β), 75.52, 75.30, 74.77, 74.27, 73.87 (C 2^{i}β), 72.93, 72.55 (C-3^{iii}β), 71.32, 71.20 (C-2^{i}α), 70.09 (C-2^{iii}β), 68.58 (C-4^{iii}β), 68.09 (C-4^{ii}), 61.07 (C-6^{iii}β), 60.56 (C-6^{ii}β), 60.03, 59.90 (C-6^{i}). HRMS (ESI-TOF) *m/z* calculated for $C_{18}H_{32}O_{16}Na$ [M+Na]$^+$: 527.1583, found 527.1569.

β-D-Gal^{iv}-(1→3)-β-D-Glc^{iii}-(1→4)-β-D-Glc^{ii}-(1→4)-D-Glc^{i} (22)

Compound **22** was prepared from **Gal1P** and cellotriose **18** according to the procedure described in Section 7. Yield 47%. ^{1}H NMR (600 MHz, D_2O) δ 5.07 (d, *J* = 3.8 Hz, 0.6 H, H-1^{i} α), 4.51 (d, *J* = 7.1 Hz, 1 H, H-1^{iv} β), 4.50 (d, *J* = 3.8 Hz, 0.4 H, H-1^{i} β), 4.41 – 4.34 (m, 1.5H), 3.85 – 3.30 (m, 24H), 3.29 – 3.10 (m, 6H). ^{13}C NMR (151 MHz, D_2O) δ 103.26 (C-1^{iv} β), 102.76 (C-1^{iii} β), 102.54 (C-1^{ii} β), 102.30 (C-1^{ii} α), 95.74 (C-1^{i} β), 91.80 (C-1^{i} α), **83.93 (C-3^{iii} β)**, 78.61, 78.36, 78.35, 75.97, 75.52, 75.30 (C-5^{iv} β), 74.80, 74.23, 74.04, 73.88, 73.41, 72.93, 72.55 (C-3^{iv} β), 71.28, 71.21 (C-2^{i} α), 70.10, 69.56 (C-2^{iv} β), 68.59 (C-4^{iv} β), 68.09 (C-4^{iii} β), 67.97, 61.07 (C-6^{iv} β), 60.68, 60.56, 59.99, 59.87. HRMS (ESI-TOF) *m/z* calculated for $C_{24}H_{42}O_{21}Na$ $[M+Na]^+$: 689.2116 found 689.2119.

β-D-Gal^{iv}-(1→3)-β-D-Glc^{iii}-(1→4)-β-D-Glc^{ii}-(1→3)-D-Glc^{i} (23)

Compound **23** was prepared from **Gal1P** and trisaccharide **2** according to the procedure described in Section 7. Yield 80%. ^{1}H NMR (600 MHz, D_2O) δ 5.08 (d, *J* = 3.8 Hz, 0.6 H, H-1^{i} α), 4.51 (d, *J* = 8.0 Hz, 0.4 H, H-1^{i} β), 4.39 (d, *J* = 8.0 Hz, 1 H, H-1^{iii} β), 4.35 (d, *J* = 7.9 Hz, 1H, H-1^{ii} β), 3.83 (dd, *J* = 12.4, 2.2 Hz, 2 H), 3.79 – 3.43 (m, 13 H), 3.42 – 3.13 (m, 9 H). ^{13}C NMR (151 MHz, D_2O) δ 102.76 (C-1^{iv} β) 102.54 (C-1^{iii} β), 102.32 (C-1^{ii} β), 95.67 (C-1^{i} β), 92.01 (C-1^{i} α), 84.47 (C-3^{i} β), **83.92 (C-3^{iii} β)**, 82.21 (C-3^{i} -α), 78.51 (C-4^{ii} α), 78.36 (C-4^{ii} β), 75.97, 75.57, 75.52, 74.80 (C-3^{ii} β), 74.06, 74.02, 73.80, 73.41, 73.13 (C-3^{iv} β), 72.91, 71.20 (C-4^{iv} β), 71.02, 69.56, 69.43, 68.09 (C-4^{i} α / β), 67.97 (C-4^{iii} β), 60.69, 60.55, 59.94, 59.87. HRMS (ESI-TOF) *m/z* calculated for $C_{24}H_{42}O_{21}Na$ $[M+Na]^+$: 689.2116, found 689.2126.

β-D-GlcN^{iii}-(1→3)-β-D-Glc^{ii}-(1→4)-D-Glc^{i} (24)

Compound **24** was prepared from **GlcN1P** and cellobiose **1** according to the procedure described in Section 7. Yield 74%. ^{1}H NMR (600 MHz, D_2O) δ 5.16 (d, *J* = 3.8 Hz, 0.6 H, H-1^{i} α), 4.59 (d, *J* = 7.2, Hz, H-1^{iii} β/ H-1^{i} β), 4.48 (d, *J* = 8.0 Hz, 1H, H-1^{ii} β), 3.91 – 3.83 (m, 6H), 3.82 – 3.72 (m, 3H), 3.68 (m, 5H), 3.62 – 3.49 (m, 4H), 3.49 – 3.38 (m, 7H), 3.34 (m, 2H), 3.22 (t, *J* = 8.5 Hz, 1H), 2.63 (t, *J* = 8.5 Hz, 2 H, H-2^{iii} β). ^{13}C NMR (151 MHz, D_2O) δ 103.58 (C-1^{iii} β), 102.36 (C-1^{ii} β), 95.74 (C-1^{i} β), 91.81 (C-1^{i} α), **84.54 (C-3^{ii} β)**, 78.80 (C-4^{i} β), 78.66 (C-4^{i} α), 76.11, 75.56, 74.78 (C-2^{i} α), 74.31, 73.88 (C-2^{i} β), 72.90, 71.35, 71.20, 70.10, 69.67 (C-3^{iii} β), 68.05, 60.74, 60.53, 60.05, 59.92, **56.85 (C-2^{iii} β)**. HRMS (ESI-TOF) *m/z* calculated for $C_{18}H_{33}NO_{15}Na$ $[M+Na]^+$: 526.1748 and found 526.1750.

β-D-Man^{iii}-(1→3)-β-D-Glc^{ii}-(1→4)-D-Glc^{i} (25)

Compound **25** was prepared from **GlcN1P** and cellobiose **1** according to the procedure described in Section 7. Yield 80%. ^{1}H NMR (600 MHz, D_2O) δ 5.07 (d, *J* = 3.7 Hz, 0.55 H, H-1^{i} α), 4.75 (broad s, 1H, H-1^{iii} β), 4.50 (d, *J* = 8.0 Hz, 0.45 H, H-1^{i} β), 4.39 (d, *J* = 8.0 Hz, 1H, H-1^{ii} β), 3.96 (d, *J* = 3.7 Hz, 1H, H-2^{iii} β), 3.82 – 3.23 (m, 31H), 3.13 (dd, *J* = 9.0, 8.0 Hz, 1H, H-2^{i} β). ^{13}C NMR (151 MHz, D_2O) δ 102.32 (C-1^{ii} β), 100.57 (C-1^{iii} β), 95.75 (C-1^{i} β), 91.81 (C-1^{i} α), **84.25 (C-3^{ii} β)**, 78.78 (C-4^{i} β), 78.65 (C-4^{i} α), 76.37, 75.48, 74.77, 74.29, 73.88, 72.86, 71.34, 71.21, 70.56, 70.09, 68.04 (C-4^{iii} β), 66.71 (C-4^{ii} β, C-4^{i}), 61.00 (C-6^{iii} β), 60.56 (C-6^{ii} β), 60.06 (C-6^{i} β), 59.93 (C-6^{i} α). HRMS (ESI-TOF) *m/z* calculated for $C_{18}H_{32}O_{16}Na$ $[M+Na]^+$: 527.1583, found 527.1580.

12. References

1. E. C. O'Neill, G. Pergolizzi, C. E. M. Stevenson, D. M. Lawson, S. A. Nepogodiev and R. A. Field, *Carbohydr. Res.*, 2017, **451**, 118-132.
2. S. Kuhaudomlarp, N. J. Patron, B. Henrissat, M. Rejzek, G. Saalbach and R. A. Field, *J. Biol. Chem.*, 2018, **293**, 2865-2876.
3. M. R. De Groeve, G. H. Tran, A. Van Hoorebeke, J. Stout, T. Desmet, S. N. Savvides and W. Soetaert, *Anal. Biochem.*, 2010, **401**, 162-167.
4. E. Yoon and R. A. Laine, *Glycobiology*, 1992, **2**, 161-168.
5. K.P. R. Kartha, R. A. Field. Iodine: A *Tetrahedron,* 1997, **2**, 11753-11766.
6. T. Ren and D. Liu. *Tetrahedron Lett.*, 1999, **40**, 7621-7625.
7. J.-S. Zhu, N. E. McCormick, S. C. Timmons, D. L. Jakeman. *J. Org. Chem.* 2016, **81**, 8816-8825.
8. T. Li, A. Tikad, W. Pan, Y. Brissonnet, S. P. Vincent. Stereocontrolled β- and α-Phosphorylations of D-Mannose. In: *Carbohydrate Chemistry Proven Synthetic Methods*. Vol. 3, Roy, R. and Vidal, S. (Eds.), CRC Press, 2015, 133-140.
9. S. Sabesan, S. Neira. *Carbohydr. Res.*, 1992, **223**, 169-185.
10. J. F. Billing, U. J. Nilsson. *Tetrahedron* 2005, **61**, 863-874.
11. FrankMaley, Gladys F. Maley and Henry A. Lardy. *J. Am. Chem. Soc.,* 1956, **78**, 5303.
12. D. Lazarevic, J. Thiem. *Carbohydr. Res*. 2006, **341**, 569-576.

13. NMR spectra of sugar phosphates

α-D-Mannopyranose-1-phosphate bis(triethylammonium salt)

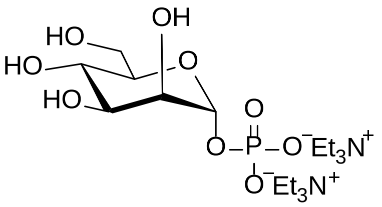

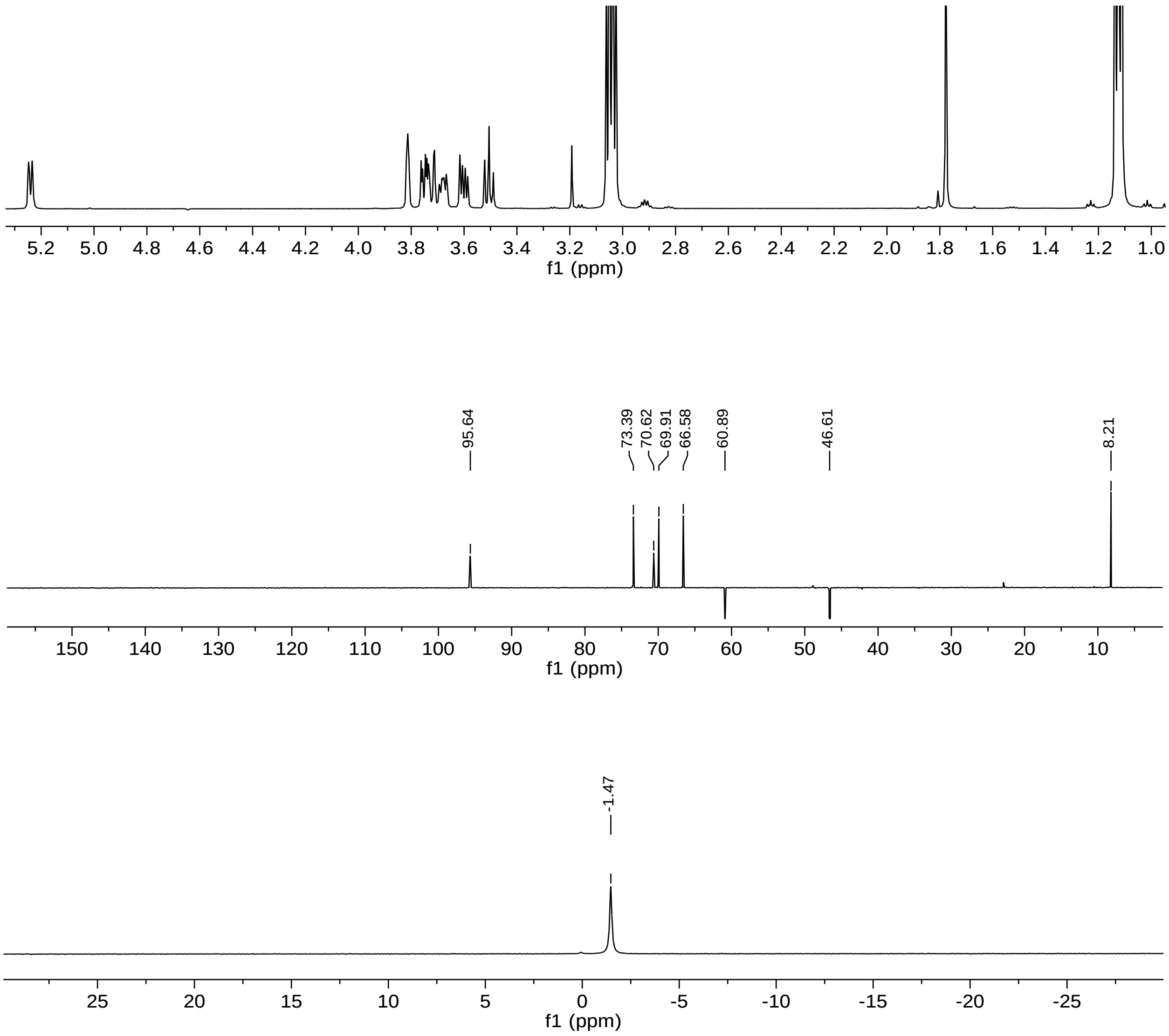

Figure S11. ^{1}H (400 MHz), ^{13}C (100.6 MHz) and ^{31}P (162 MHz) NMR spectra of α-D-mannopyranose-1-phosphate bis(triethylammonium salt).

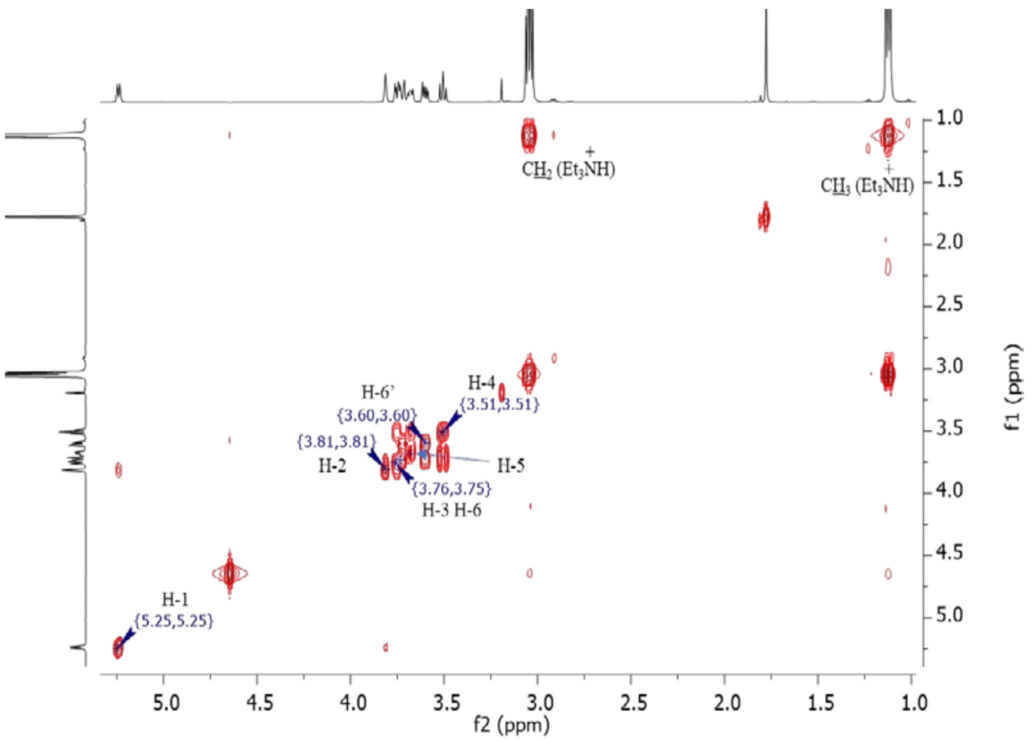

Figure S12. 2D-COSY of α-D-mannopyranose-1-phosphate bis(triethylammonium salt).

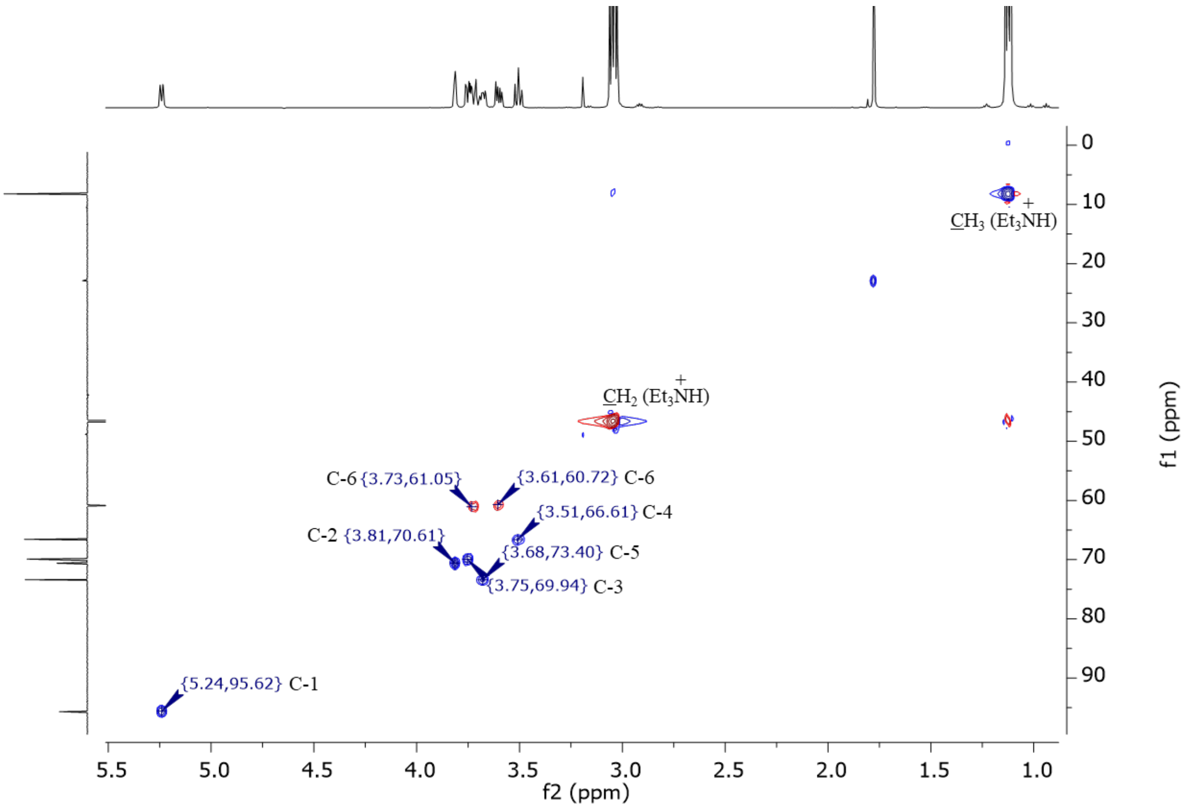

Figure S13. 2D-HSQC of α-D-mannopyranose-1-phosphate bis(triethylammonium salt).

Amino-2-deoxy-α-D-glucopyranose-1-phosphate disodium salt

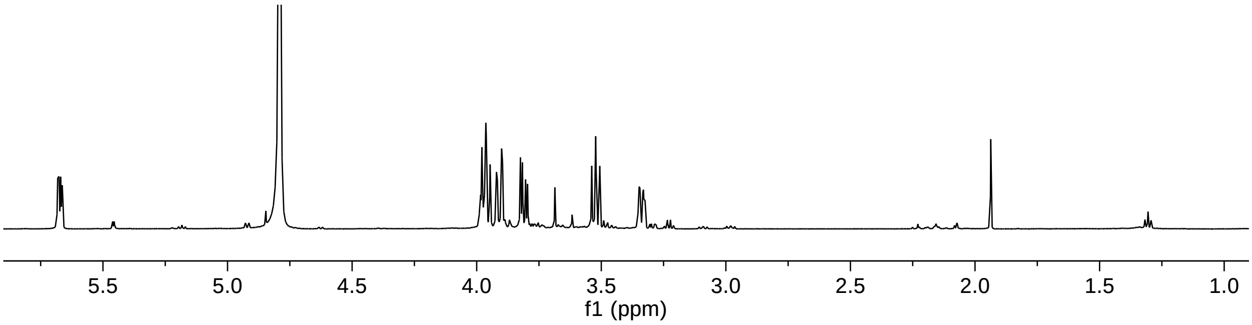

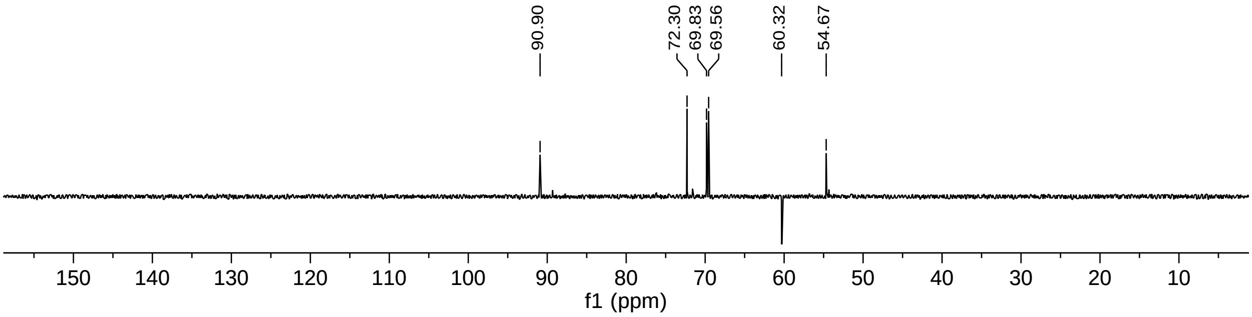

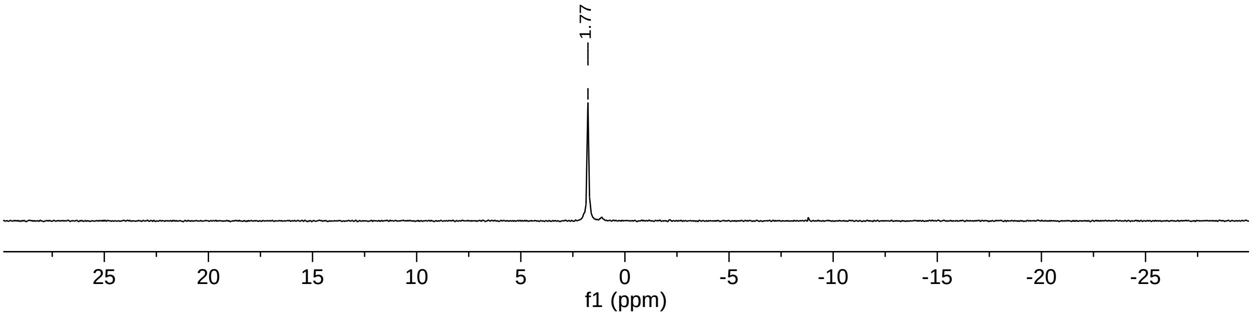

Figure S14. ^{1}H (400 MHz), ^{13}C (100.6 MHz) and ^{31}P (162 MHz) NMR spectra of 2-amino-2-deoxy-α-D-glucopyranose-1-phosphate disodium salt.

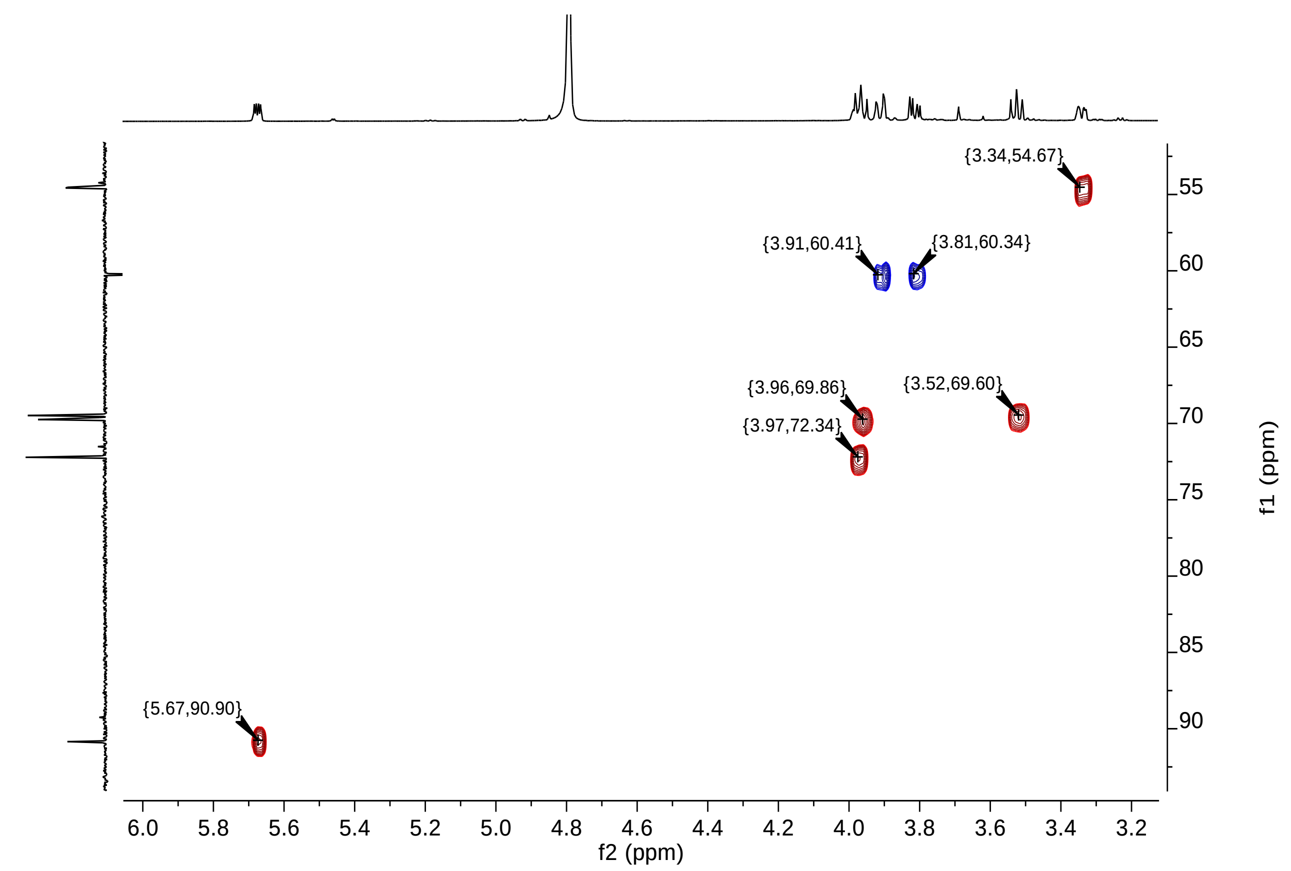

Figure S15. 2D-HSQC of 2-amino-2-deoxy-α-D-gluciopyranose-1-phosphate disodium salt.

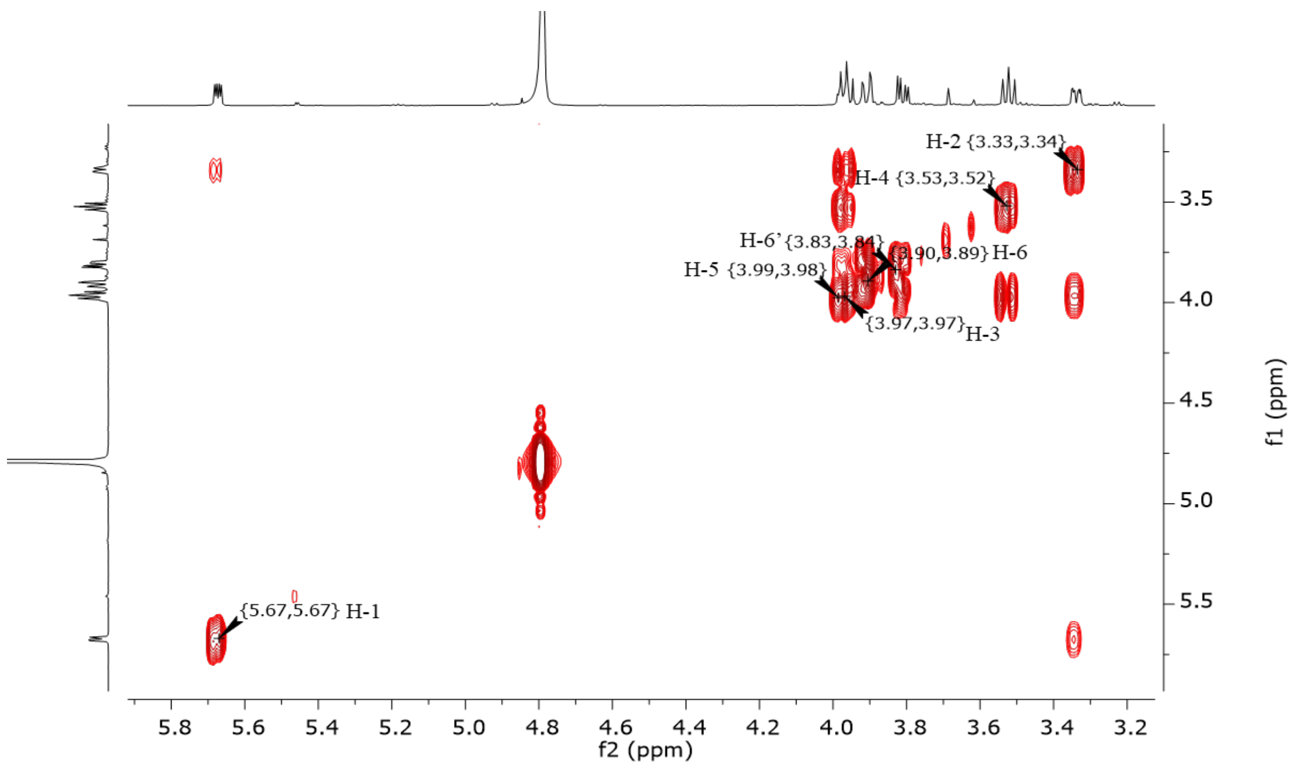

Figure S16. 2D-COSY of 2-amino-2-deoxy-α-D-gluciopyranose-1-phosphate disodium salt.

14. NMR characterization of oligosaccharides synthesised by enzymatic glycosylation with CDP and Pro_7066

β-D-Gal^{iii}-(1→4)-β-D-Glc^{ii}-(1→4)-D-Glc^{i} (9)

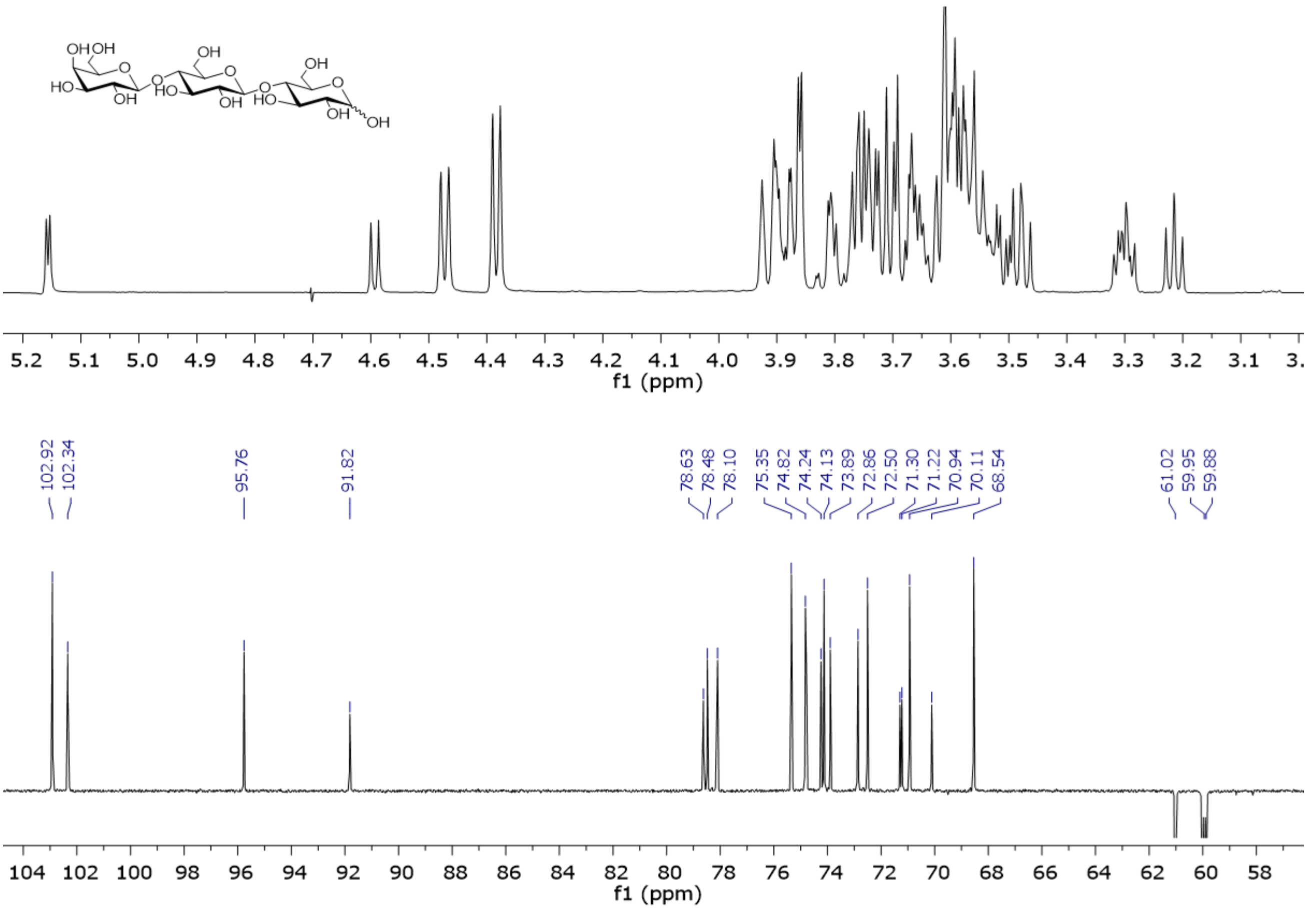

Figure S17. ^{1}H and ^{13}C (DEPT135) NMR spectra (400 MHz, D_2O) of β-D-Gal^{iii}-(1→4)-β-D-Glc^{ii}-(1→4)-D-Glc^{i} (**9**).

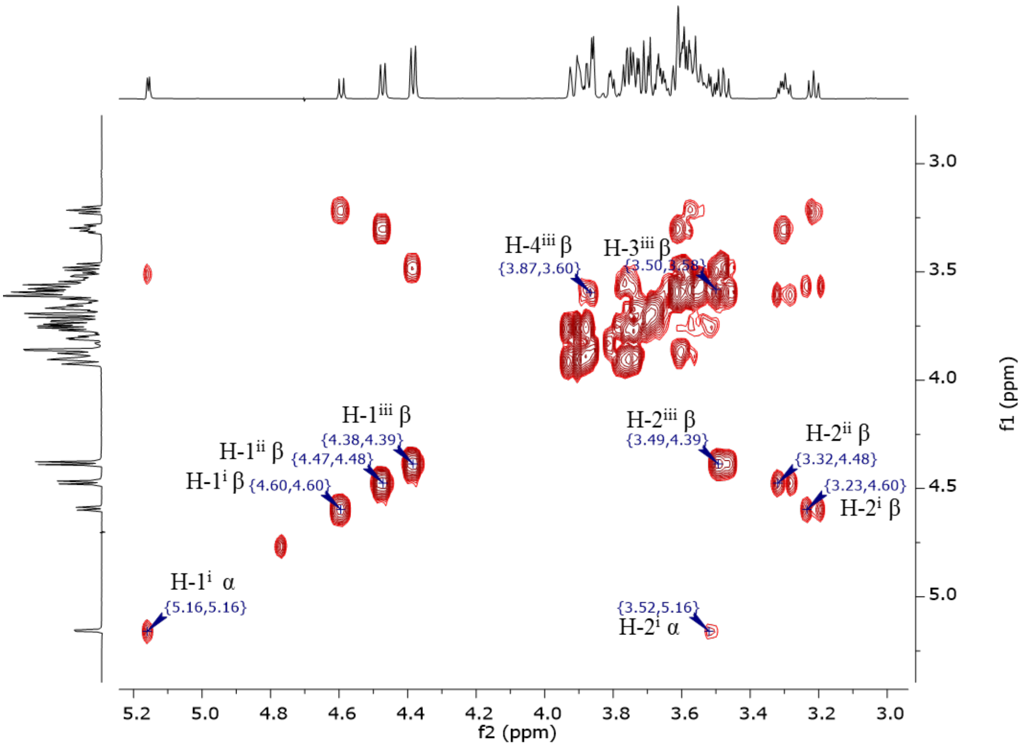

Figure S18. 2D-COSY of β-D-Gal^{iii}-(1→4)-β-D-Glc^{ii}-(1→4)-D-Glc^{i} (**9**).

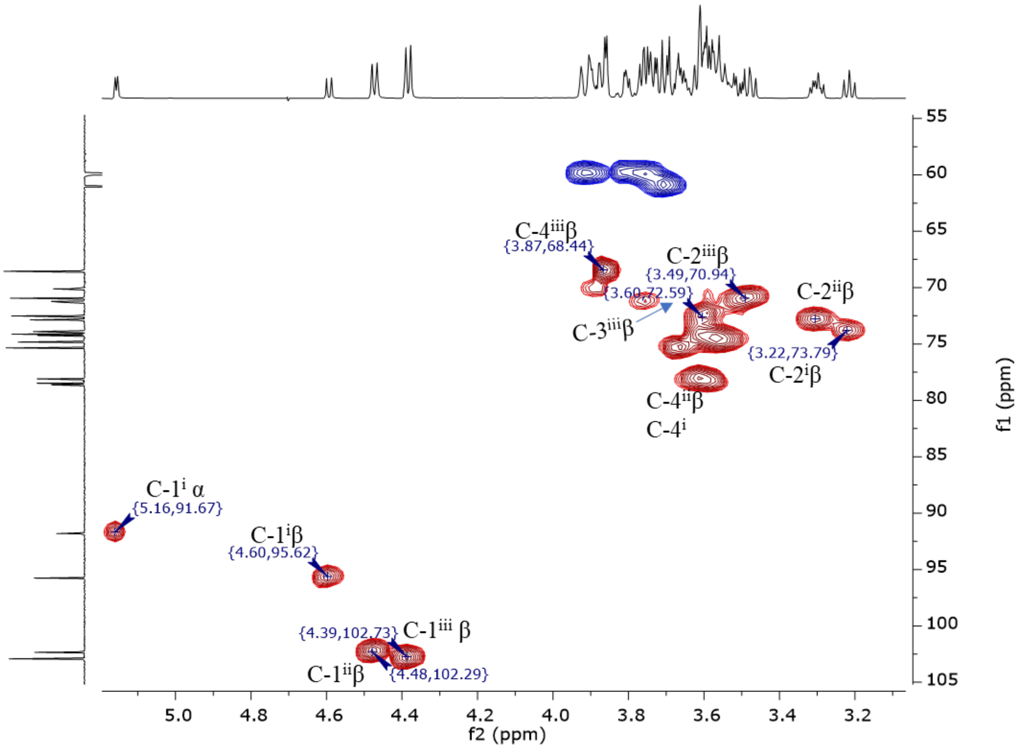

Figure S19. 2D-HSQC of β-D-Gal^{iii}-(1→4)-β-D-Glc^{ii}-(1→4)-D-Glc^{i} (**9**).

β-D-Gal^{iv}-(1→4)-β-D-Glc^{iii}-(1→4)-β-D-Glc^{ii}-(1→3)-D-Glc^{i} (10)

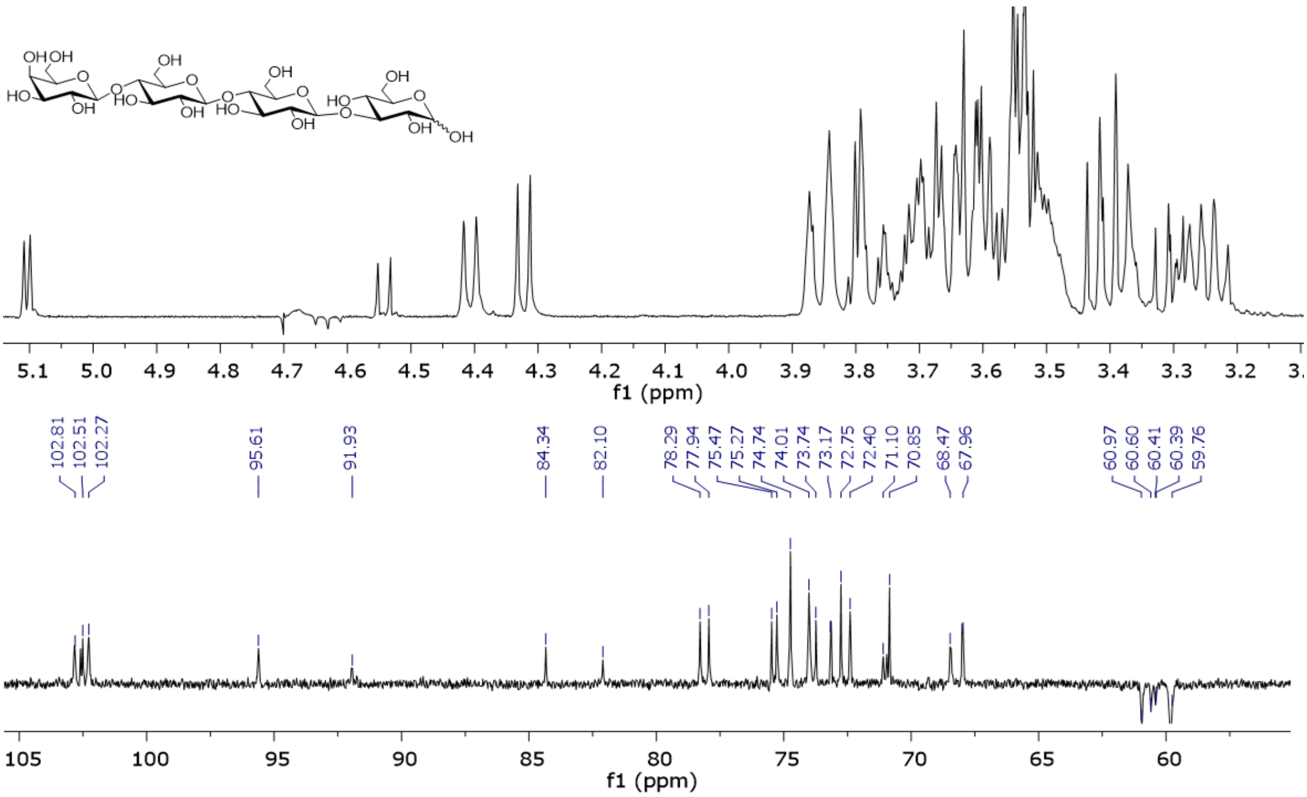

Figure S20. ^{1}H and ^{13}C (DEPT135) NMR (400 MHz, D_2O) spectra of β-D-Gal^{iv}-(1→4)-β-D-Glc^{iii}-(1→4)-β-D-Glc^{ii}-(1→3)-D-Glc^{i} (**10**).

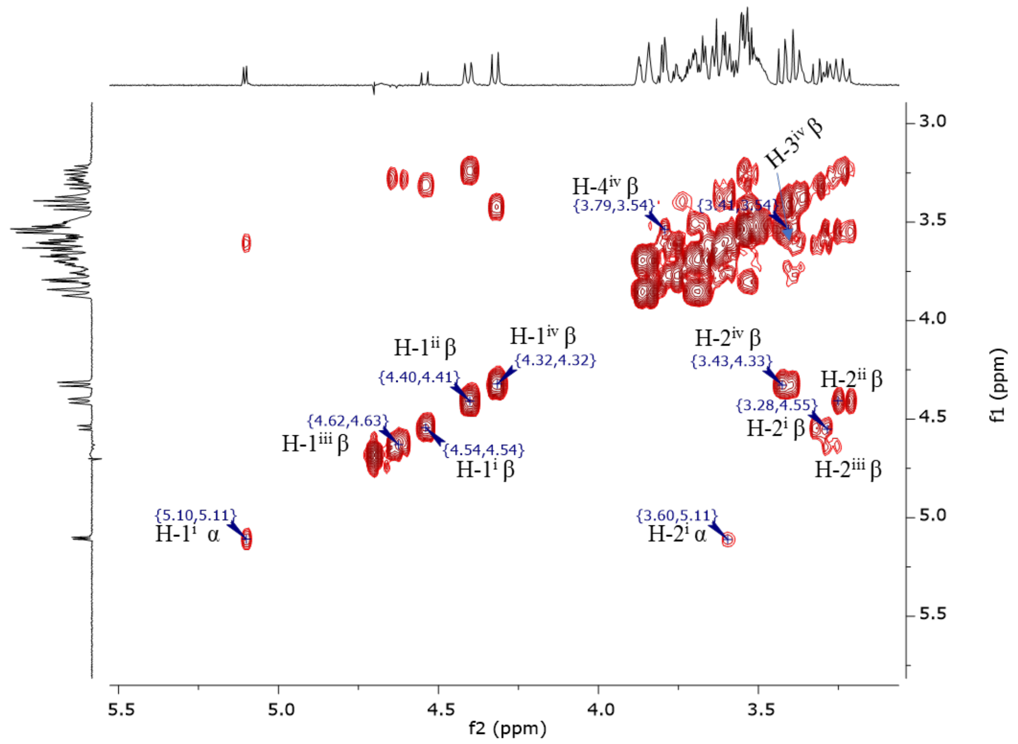

Figure S21. 2D-COSY of β-D-Gal^{iv}-(1→4)-β-D-Glc^{iii}-(1→4)-β-D-Glc^{ii}-(1→3)-D-Glc^{i} (**10**).

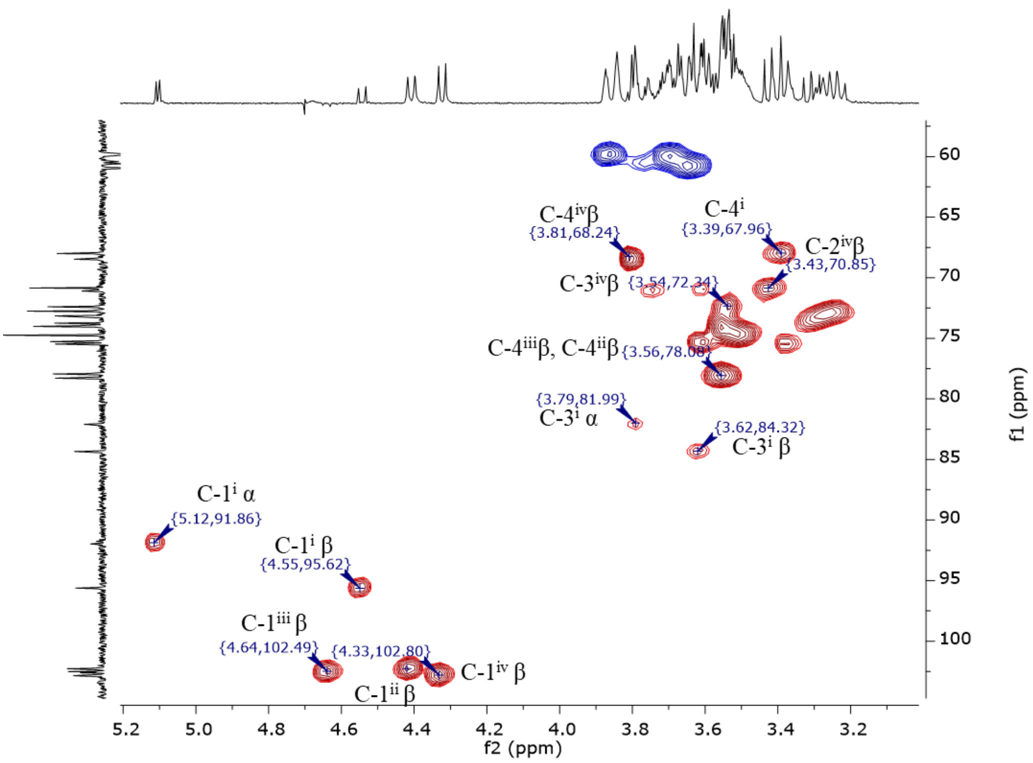

Figure S22. 2D-HSQC of β-D-Gal^{iv}-(1→4)-β-D-Glc^{iii}-(1→4)-β-D-Glc^{ii}-(1→3)-D-Glc^{i} (**10**).

β-D-Gal^{iv}-(1→4)-β-D-Glc^{iii}-(1→3)-β-D-Glc^{ii}-(1→4)-D-Glc^{i} (11)

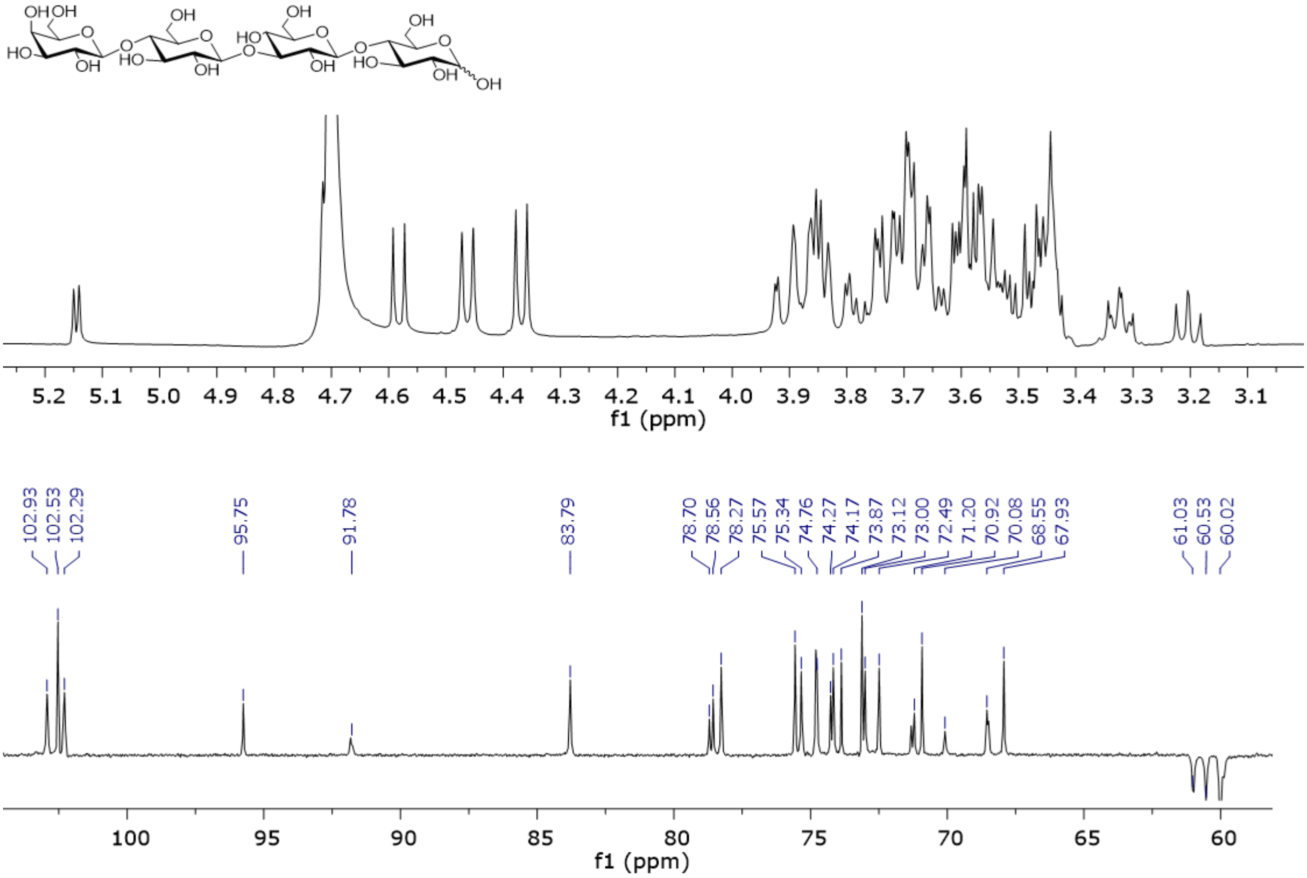

Figure S23. ^{1}H and ^{13}C (DEPT135) NMR (600 MHz, D_2O) of β-D-Gal^{iv}-(1→4)-β-D-Glc^{iii}-(1→3)-β-D-Glc^{ii}-(1→4)-D-Glc^{i} (**11**).

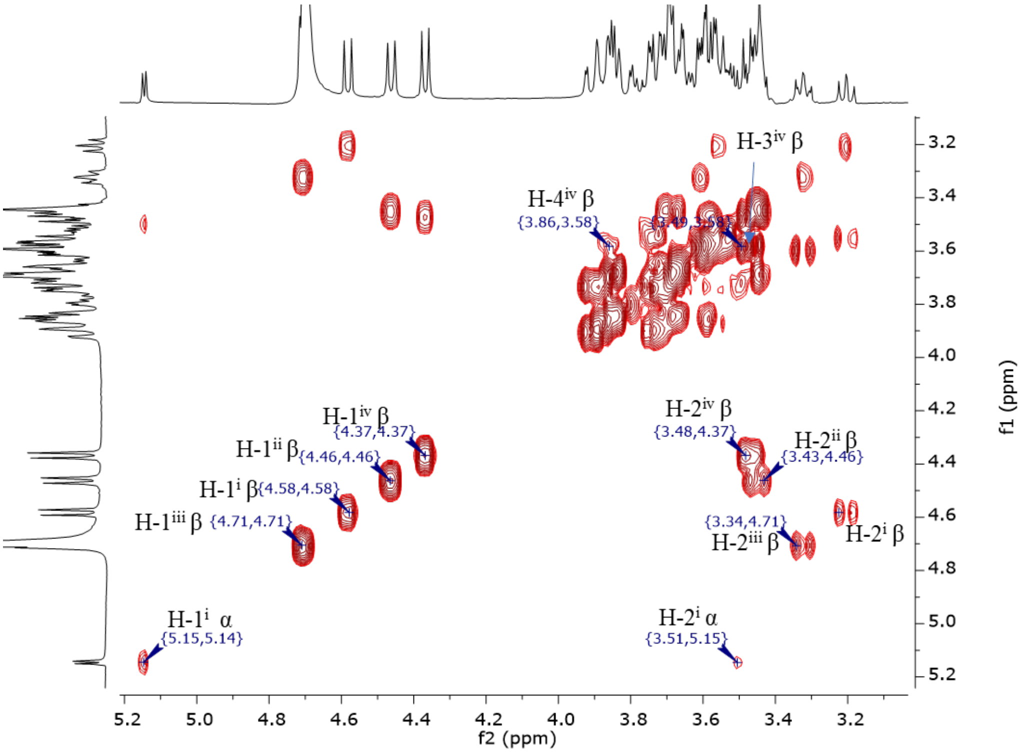

Figure S24. 2D-COSY of β-D-Gal^{iv}-(1→4)-β-D-Glc^{iii}-(1→3)-β-D-Glc^{ii}-(1→4)-D-Glc^{i} (**11**).

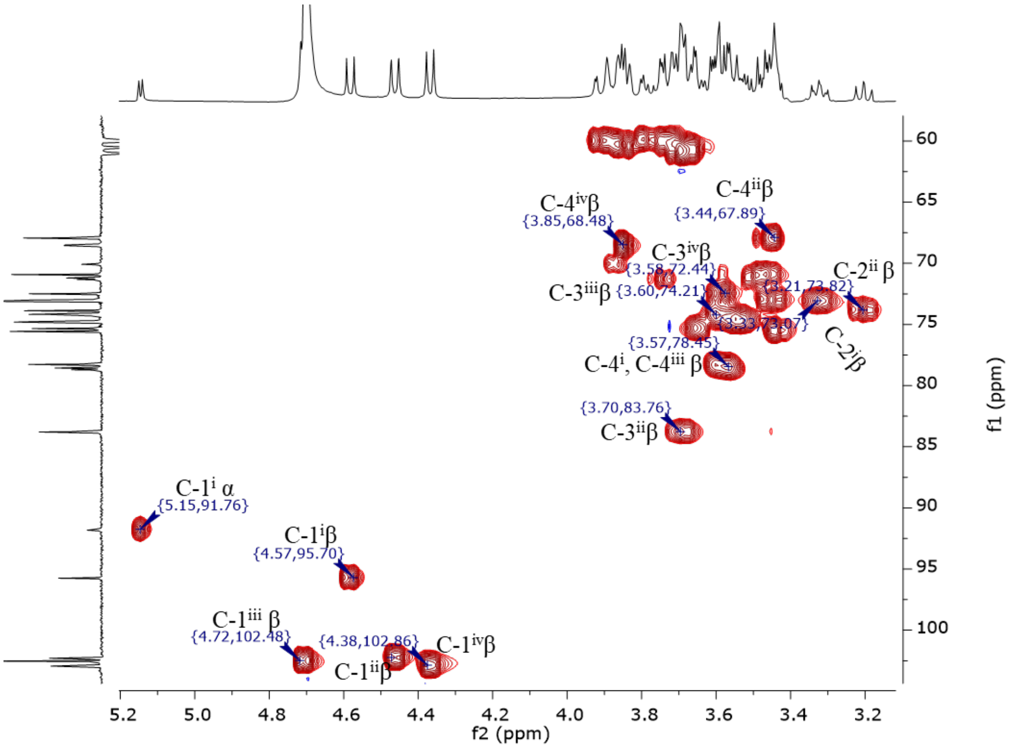

Figure S25. 2D-HSQC of β-D-Gal^{iv}-(1→4)-β-D-Glc^{iii}-(1→3)-β-D-Glc^{ii}-(1→4)-D-Glc^{i} (**11**).

β-D-Gal^{iii}-(1→4)-β-D-Glc^{ii}-(1→3)-D-Glc^{i} (12)

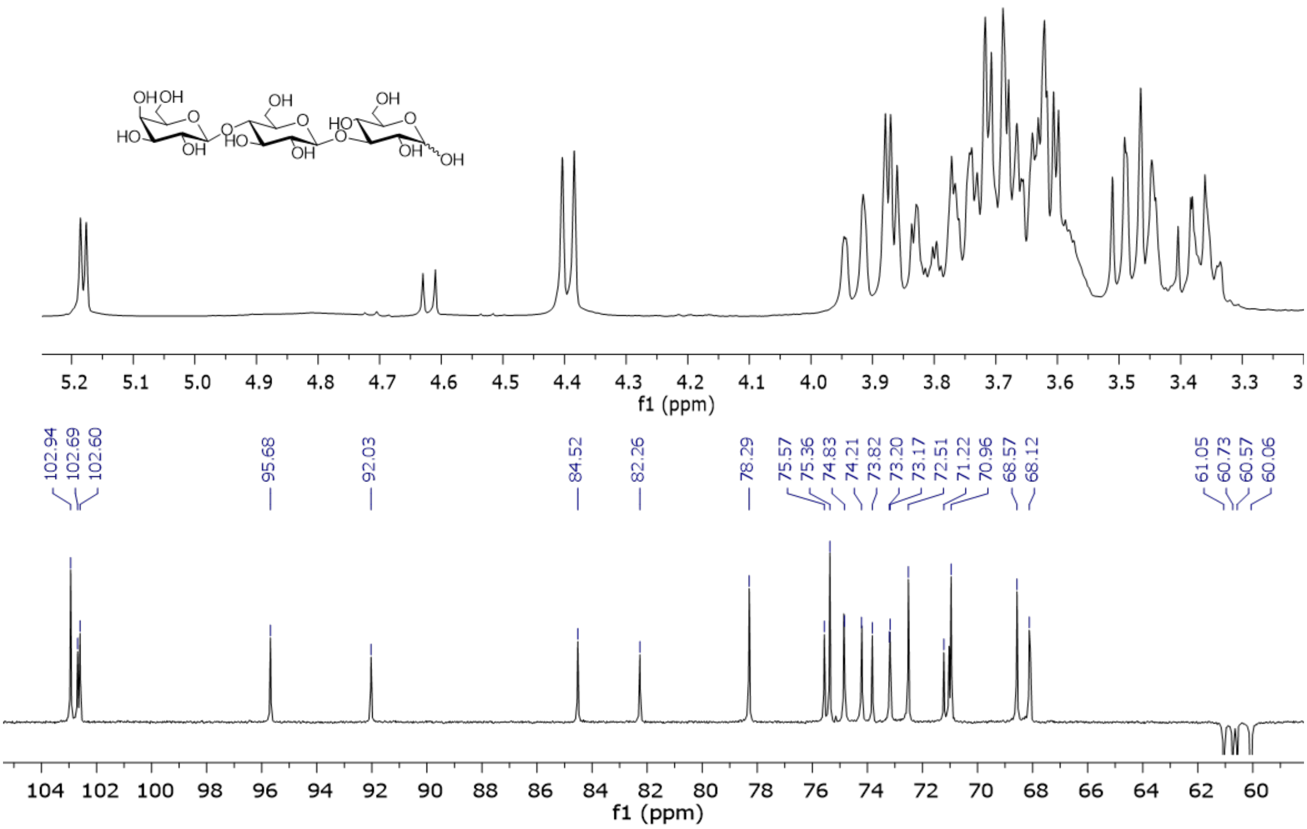

Figure S26. ^{1}H and ^{13}C (DEPT135) NMR (600 MHz, D_2O) of β-D-Gal^{iii}-(1→4)-β-D-Glc^{ii}-(1→3)-D-Glc^{i} (**2**).

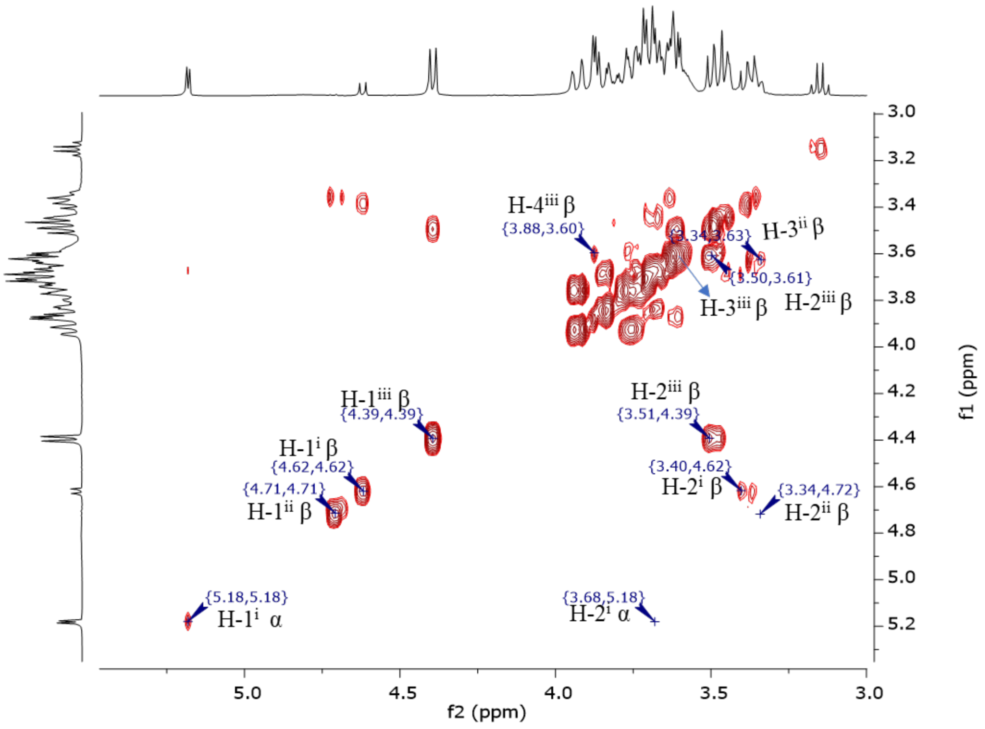

Figure S27. 2D-COSY of β-D-Gal^{iii}-(1→4)-β-D-Glc^{ii}-(1→3)-D-Glc^{i} (**12**).

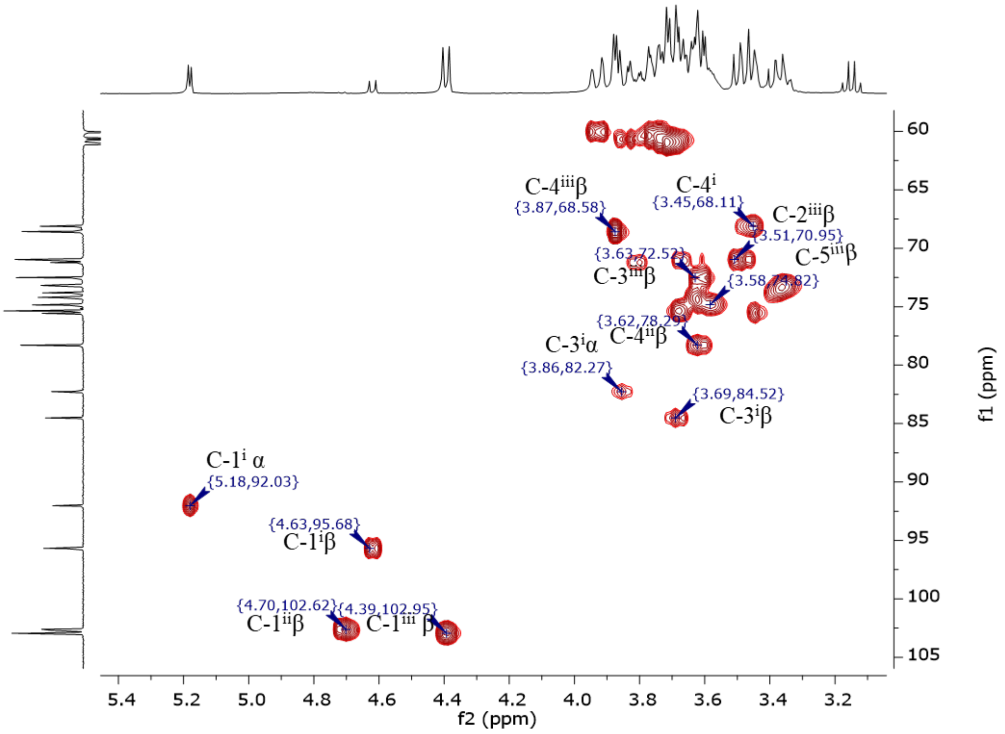

Figure S28. 2D-HSQC of β-D-Gal^{iii}-(1→4)-β-D-Glc^{ii}-(1→3)-D-Glc^{i} (**12**).

β-D-Gal^{iv}-(1→4)-β-D-Glc^{iii}-(1→3)-β-D-Glc^{ii}-(1→3)-D-Glc^{i} (13)

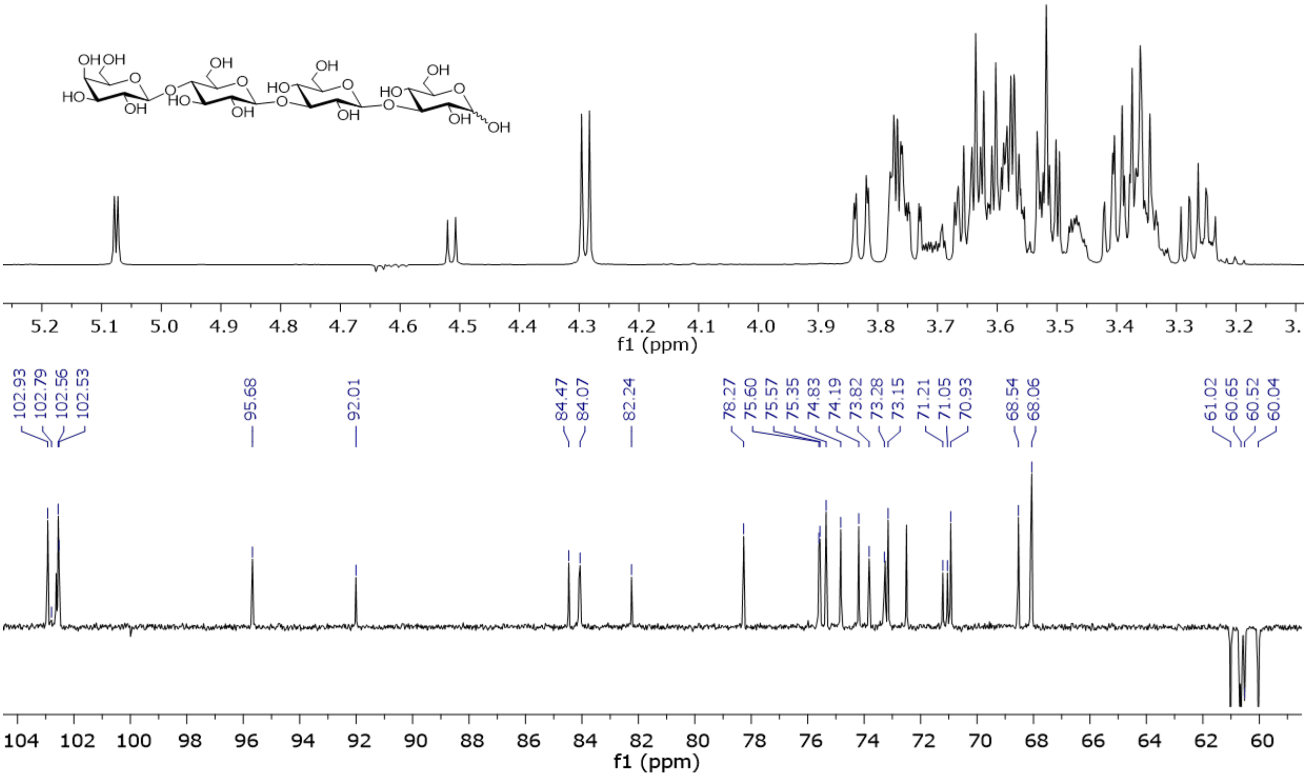

Figure S29. ^{1}H and ^{13}C (DEPT135) NMR (600 MHz, D_2O) of β-D-Gal^{iv}-(1→4)-β-D-Glc^{iii}-(1→3)-β-D-Glc^{ii}-(1→3)-D-Glc^{i} (**13**).

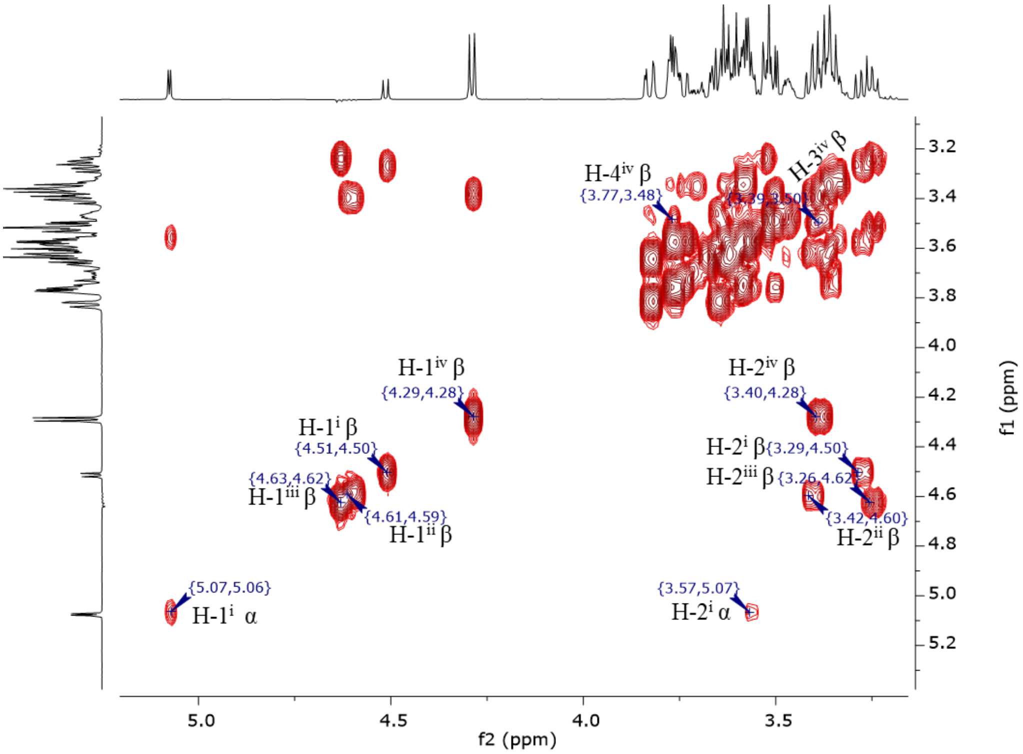

Figure S30. 2D-COSY of β-D-Gal^{iv}-(1→4)-β-D-Glc^{iii}-(1→3)-β-D-Glc^{ii}-(1→3)-D-Glc^{i} (**13**).

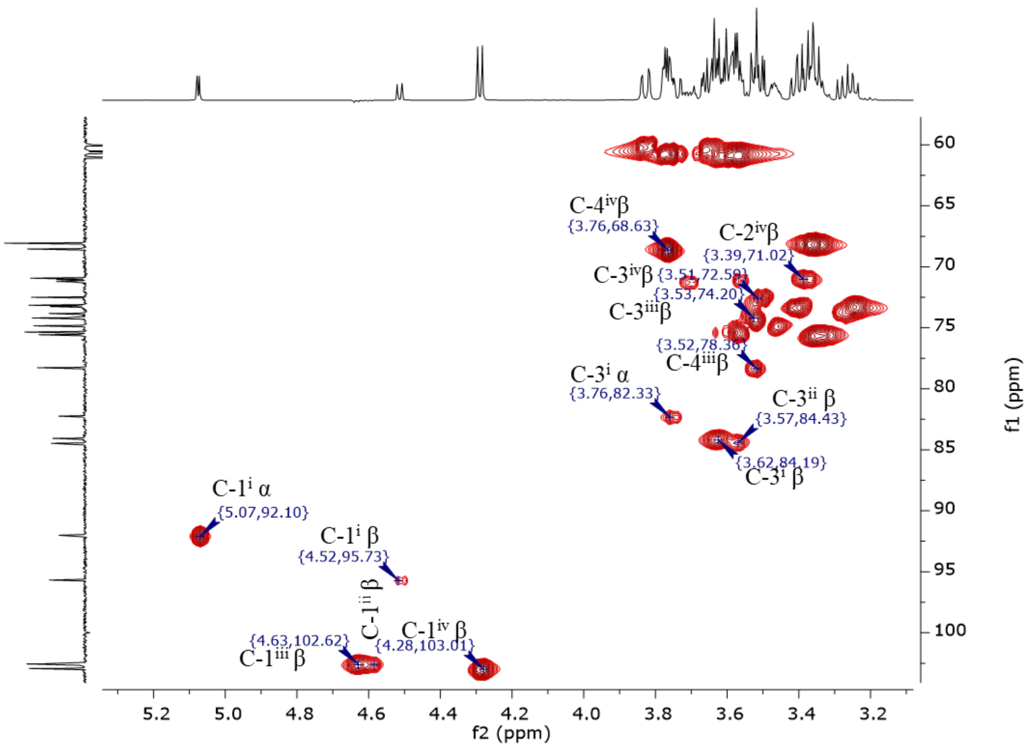

Figure S31. 2D-HSQC of β-D-Gal^{iv}-(1→4)-β-D-Glc^{iii}-(1→3)-β-D-Glc^{ii}-(1→3)-D-Glc^{i} (**13**).

β-D-Gal^{v}-(1→4)-β-D-Glc^{iv}-(1→3)-β-D-Glc^{iii}-(1→3)-β-D-Glc^{ii}-(1→3)-D-Glc^{i} (14)

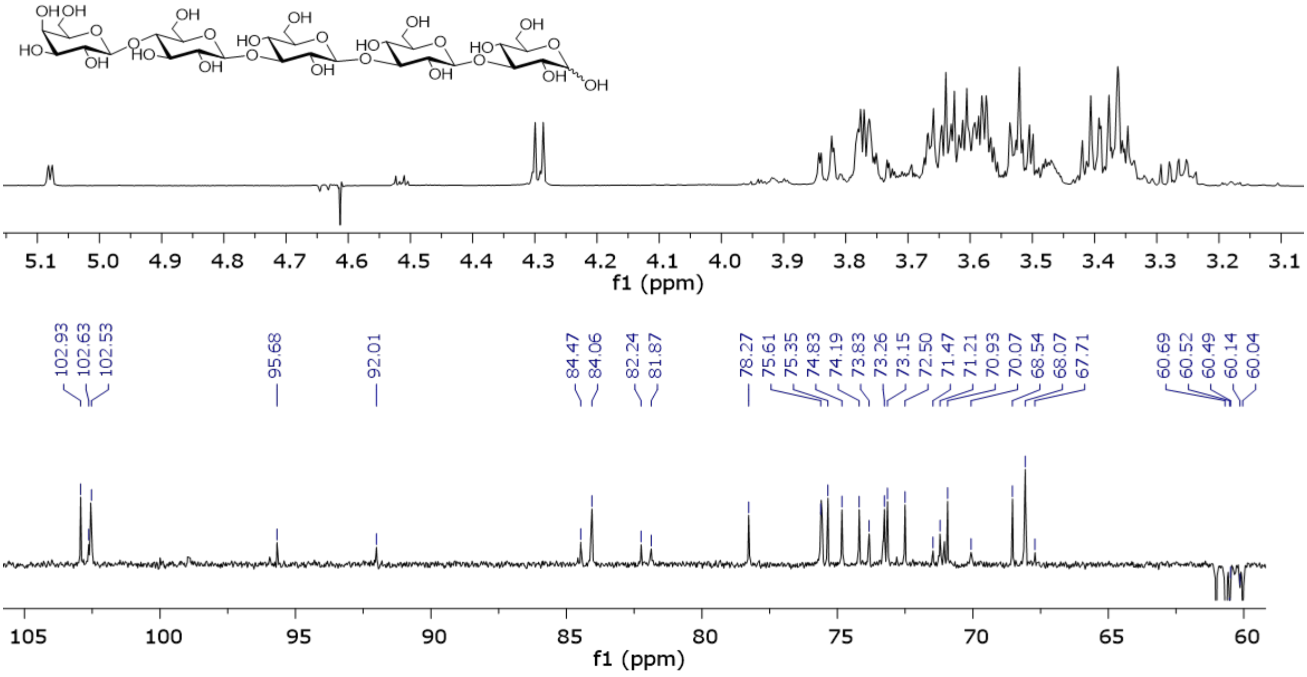

Figure S32. ^{1}H and ^{13}C (DEPT135) NMR (600 MHz, D_2O) of β-D-Gal^{v}-(1→4)-β-D-Glc^{iv}-(1→3)-β-D-Glc^{iii}-(1→3)-β-D-Glc^{ii}-(1→3)-D-Glc^{i} (**14**).

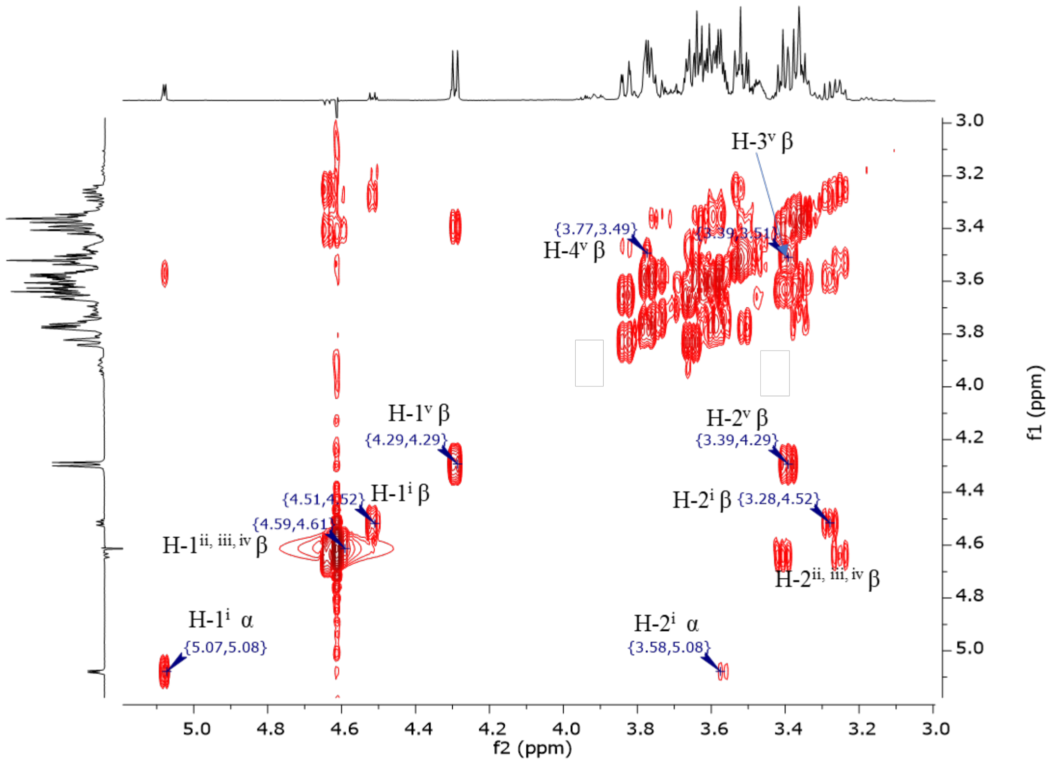

Figure S33. 2D-COSY of β-D-Gal^{v}-(1→4)-β-D-Glc^{iv}-(1→3)-β-D-Glc^{iii}-(1→3)-β-D-Glc^{ii}-(1→3)-D-Glc^{i} (**14**).

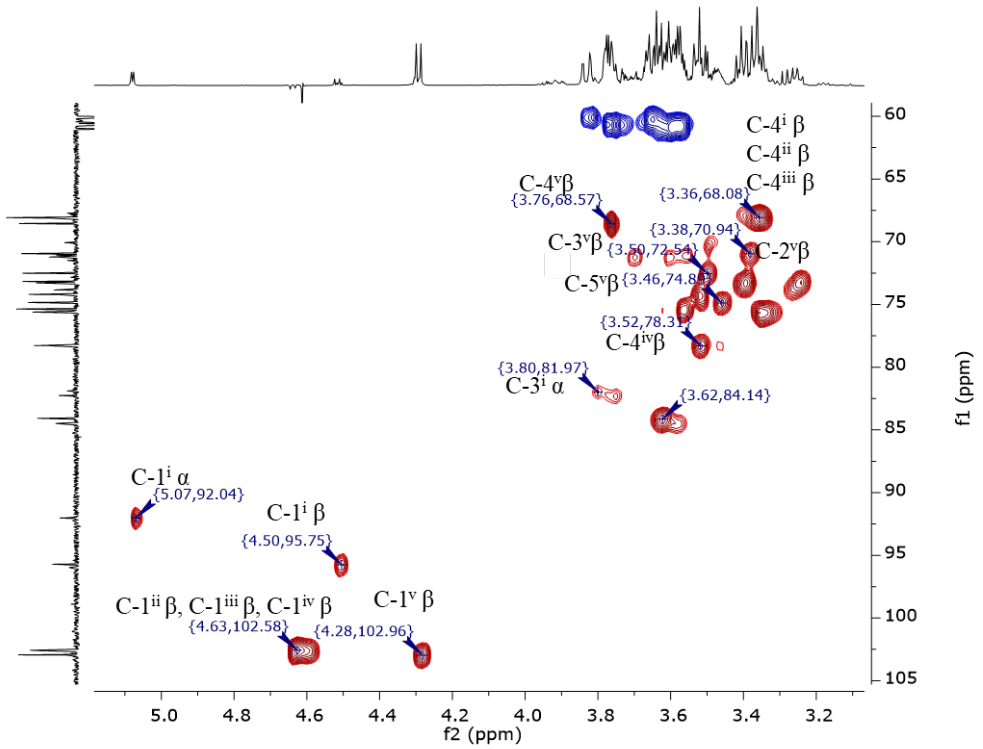

Figure S34. 2D-HSQC of β-D-Gal^{v}-(1→4)-β-D-Glc^{iv}-(1→3)-β-D-Glc^{iii}-(1→3)-β-D-Glc^{ii}-(1→3)-D-Glc^{i} (**14**).

β-D-Gal^{vi}-(1→4)-β-D-Glc^{v}-(1→3)-β-D-Glc^{iv}-(1→3)-β-D-Glc^{iii}-(1→3)-β-D-Glc^{ii}-(1→3)-D-Glci (15)

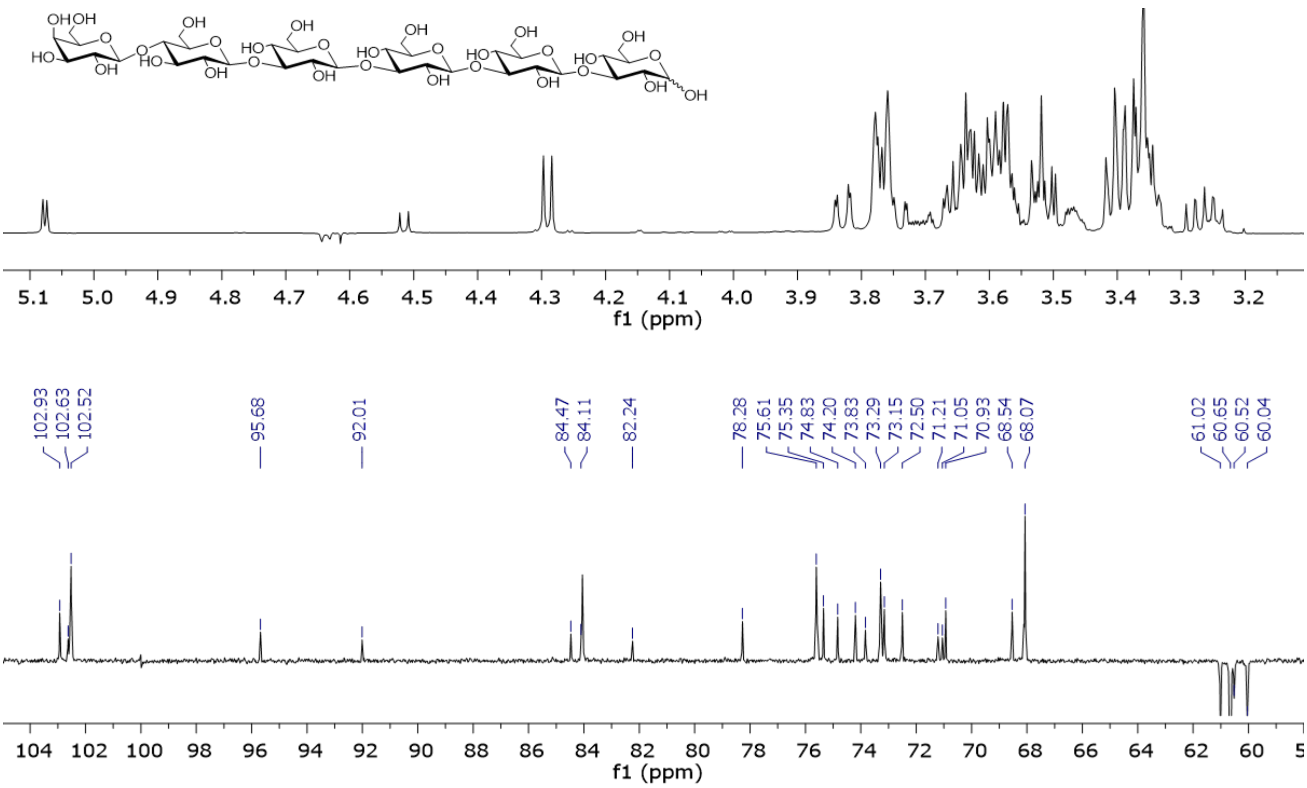

Figure S35. ^{1}H and ^{13}C (DEPT135) NMR (600 MHz, D_2O) of β-D-Gal^{vi}-(1→4)-β-D-Glc^{v}-(1→3)-β-D-Glc^{iv}-(1→3)-β-D-Glc^{iii}-(1→3)-β-D-Glc^{ii}-(1→3)-D-Glc^{i} (**15**).

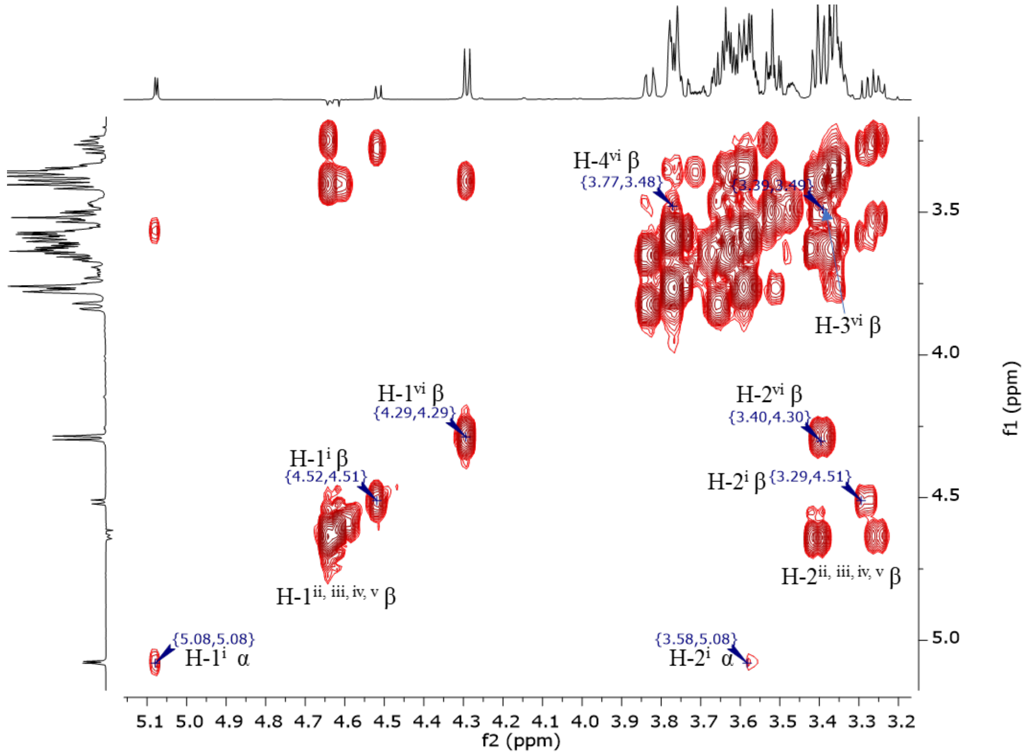

Figure S36. 2D-COSY of β-D-Gal^{vi}-(1→4)-β-D-Glc^{v}-(1→3)-β-D-Glc^{iv}-(1→3)-β-D-Glc^{iii}-(1→3)-β-D-Glc^{ii}-(1→3)-D-Glc^{i} (**15**).

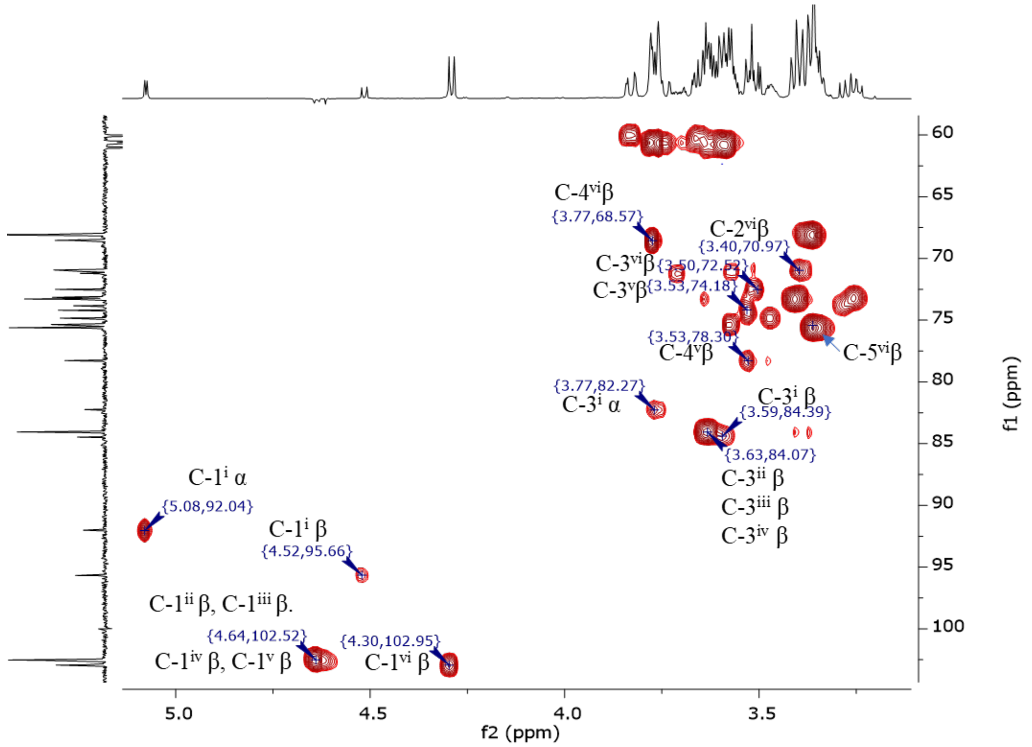

Figure S37. 2D-HSQC of β-D-Gal^{vi}-(1→4)-β-D-Glc^{v}-(1→3)-β-D-Glc^{iv}-(1→3)-β-D-Glc^{iii}-(1→3)-β-D-Glc^{ii}-(1→3)-D-Glc^{i} (**15**).

β-D-Gal^{vii}-(1→4)-β-D-Glc^{vi}-(1→3)-β-D-Glc^{v}-(1→3)-β-D-Glc^{iv}-(1→3)-β-D-Glc^{iii}-(1→3)-β-D-Glc^{ii}-(1→3)-D-Glc^{i} (16)

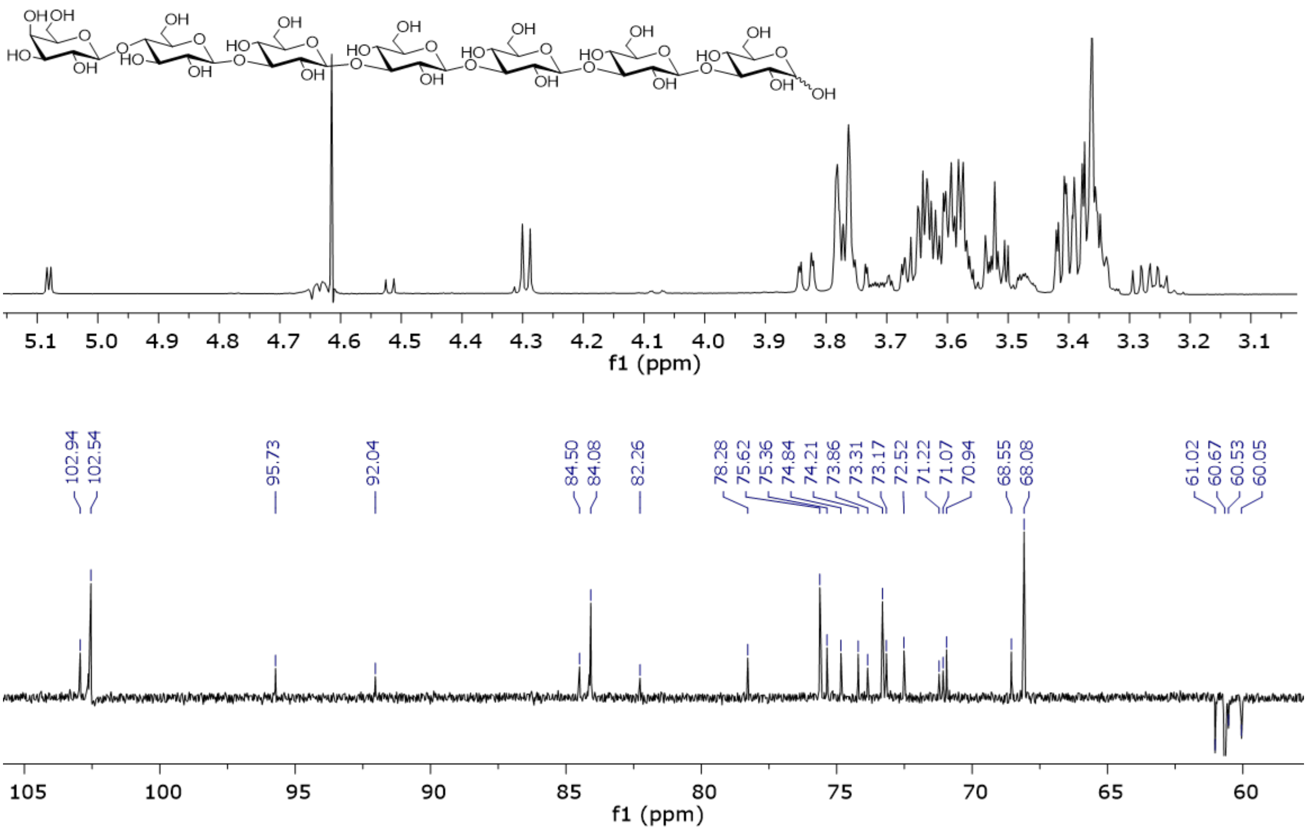

Figure S38. ^{1}H and ^{13}C (DEPT) NMR of β-D-Gal^{vii}-(1→4)-β-D-Glc^{vi}-(1→3)-β-D-Glc^{v}-(1→3)-β-D-Glc^{iv}-(1→3)-β-D-Glc^{iii}-(1→3)-β-D-Glc^{ii}-(1→3)-D-Glc^{i} (**16**).

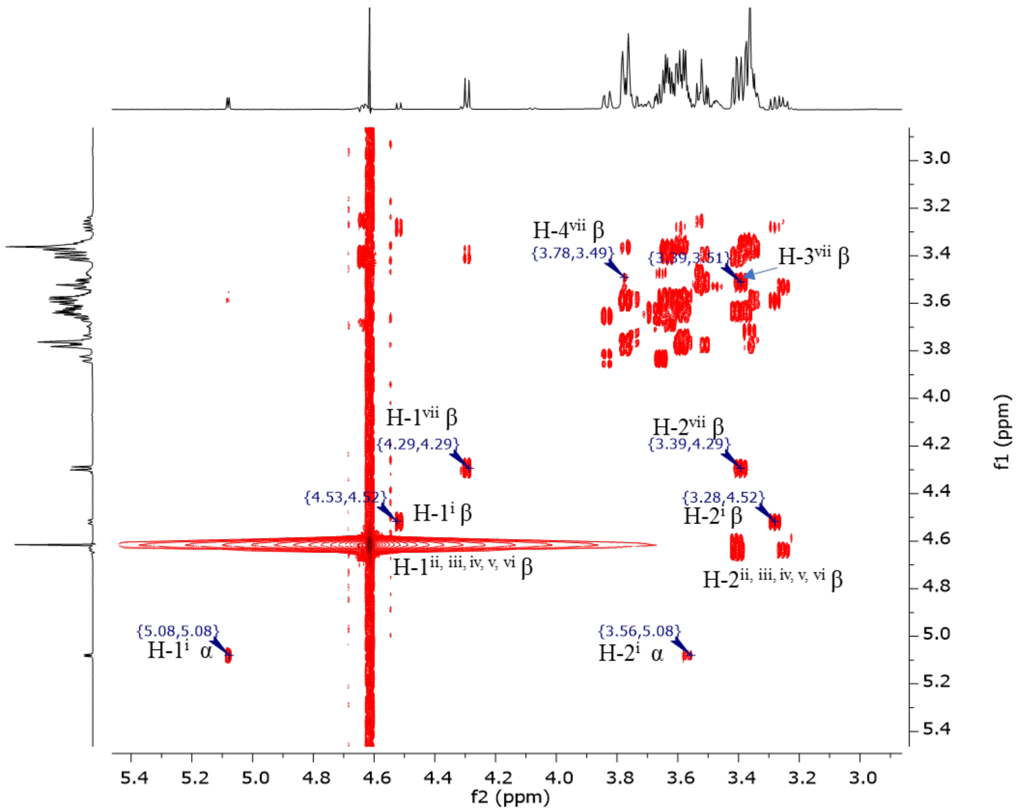

Figure S39. 2D-COSY of β-D-Gal^{vii}-(1→4)-β-D-Glc^{vi}-(1→3)-β-D-Glc^{v}-(1→3)-β-D-Glc^{iv}-(1→3)-β-D-Glc^{iii}-(1→3)-β-D-Glc^{ii}-(1→3)-D-Glc^{i} (**16**).

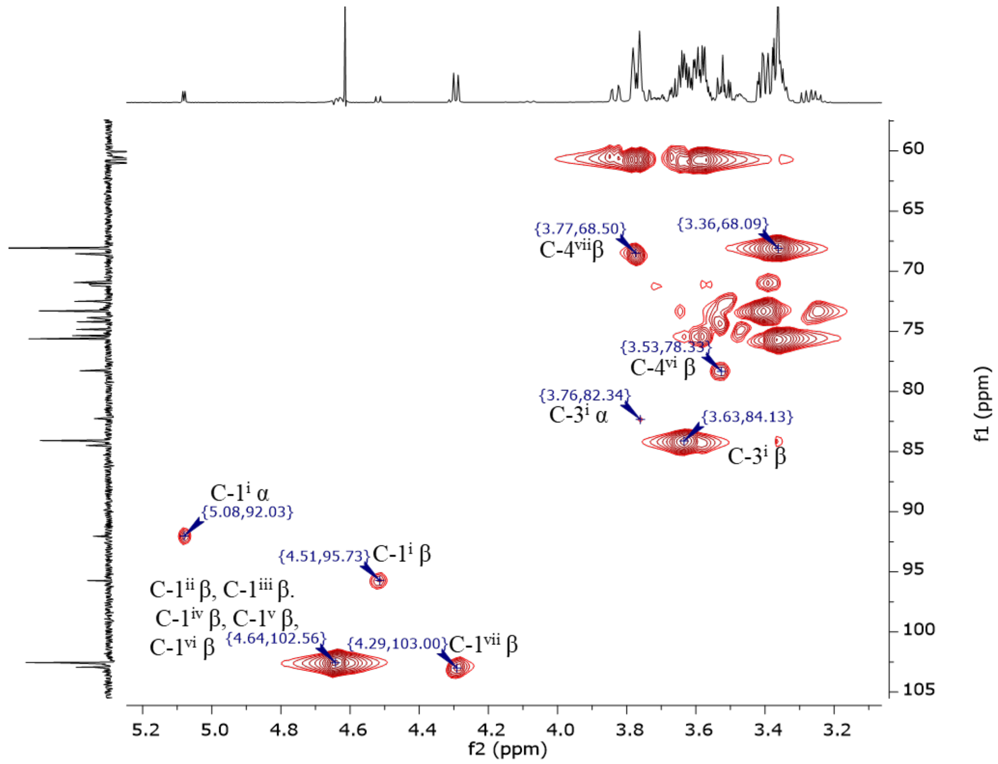

Figure S40. 2D-HSQC of β-D-Gal^{vii}-(1→4)-β-D-Glc^{vi}-(1→3)-β-D-Glc^{v}-(1→3)-β-D-Glc^{iv}-(1→3)-β-D-Glc^{iii}-(1→3)-β-D-Glc^{ii}-(1→3)-D-Glc^{i} (**16**).

β-D-GlcN^{iii}-(1→4)-β-D-Glc^{ii}-(1→3)-D-Glc^{i} (17)

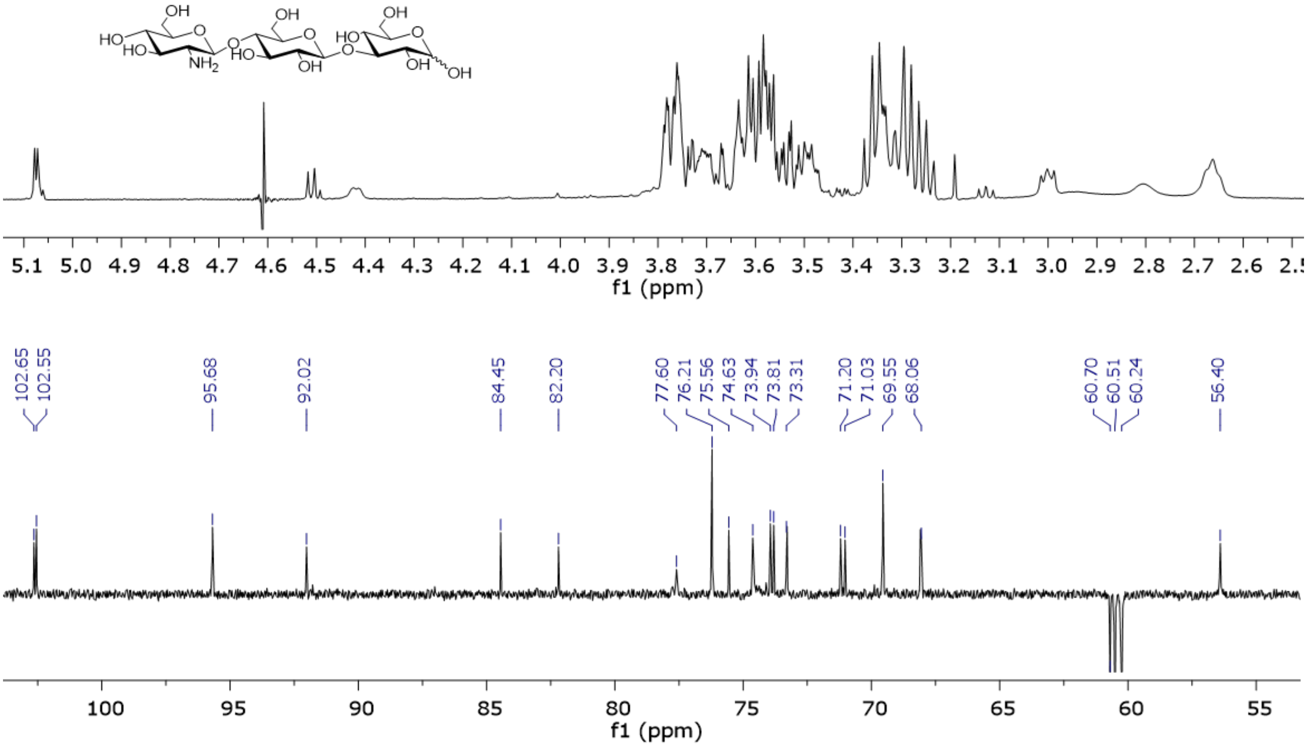

Figure S41. ^{1}H and ^{13}C (DEPT135) NMR (600 MHz, D_2O) of β-D-GlcN^{iii}-(1→4)-β-D-Glc^{ii}-(1→3)-D-Glc^{i} (**17**).

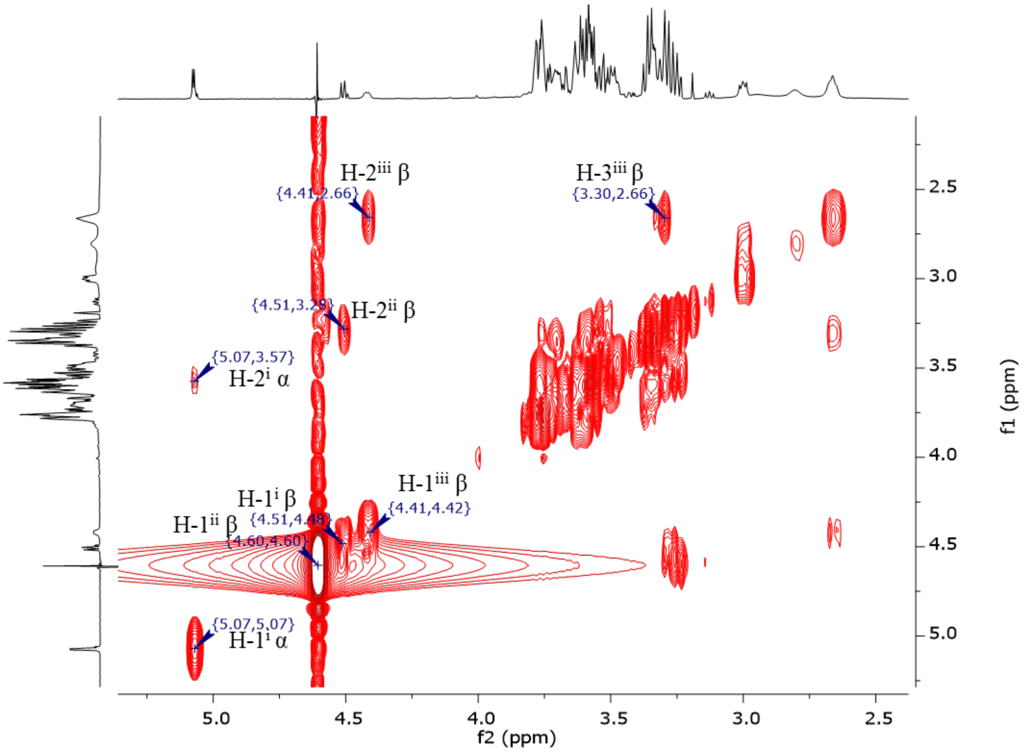

Figure S42. 2D-COSY of β-D-GlcN^{iii}-(1→4)-β-D-Glc^{ii}-(1→3)-D-Glc^{i} (**17**).

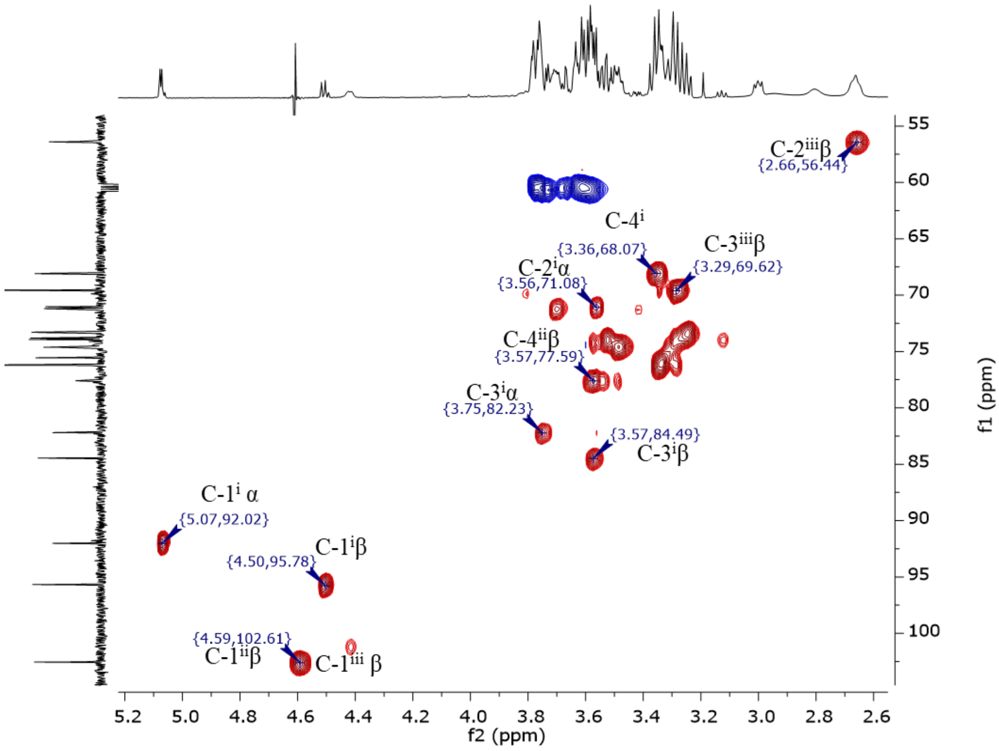

Figure S43. 2D-HSQC of β-D-GlcN^{iii}-(1→4)-β-D-Glc^{ii}-(1→3)-D-Glc^{i} (**17**).

β-D-Gal^{iii}-(1→3)-β-D-Glc^{ii}-(1→3)-D-Glc^{i} (19)

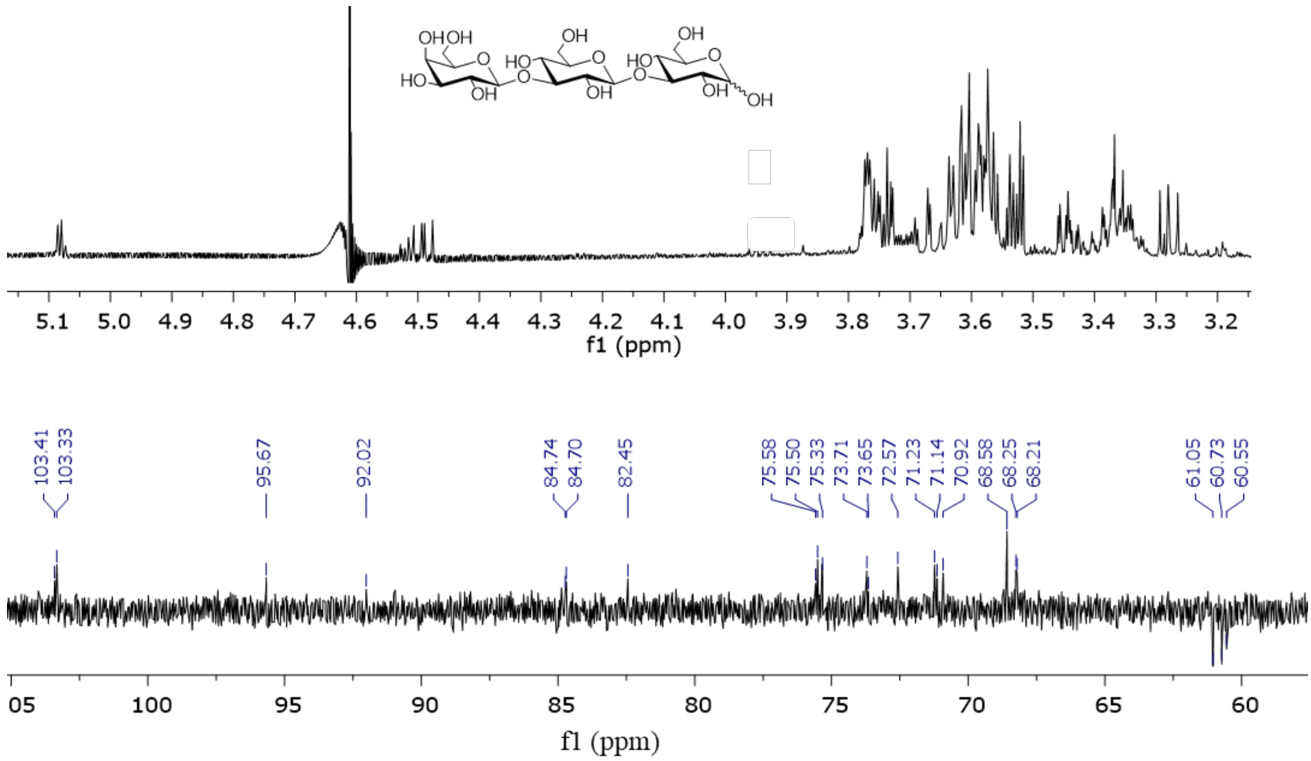

Figure S44. ^{1}H and ^{13}C (DEPT) NMR (600 MHz, D_2O)of β-D-Gal^{iii}-(1→3)-β-D-Glc^{ii}-(1→3)-D-Glc^{i} (**19**).

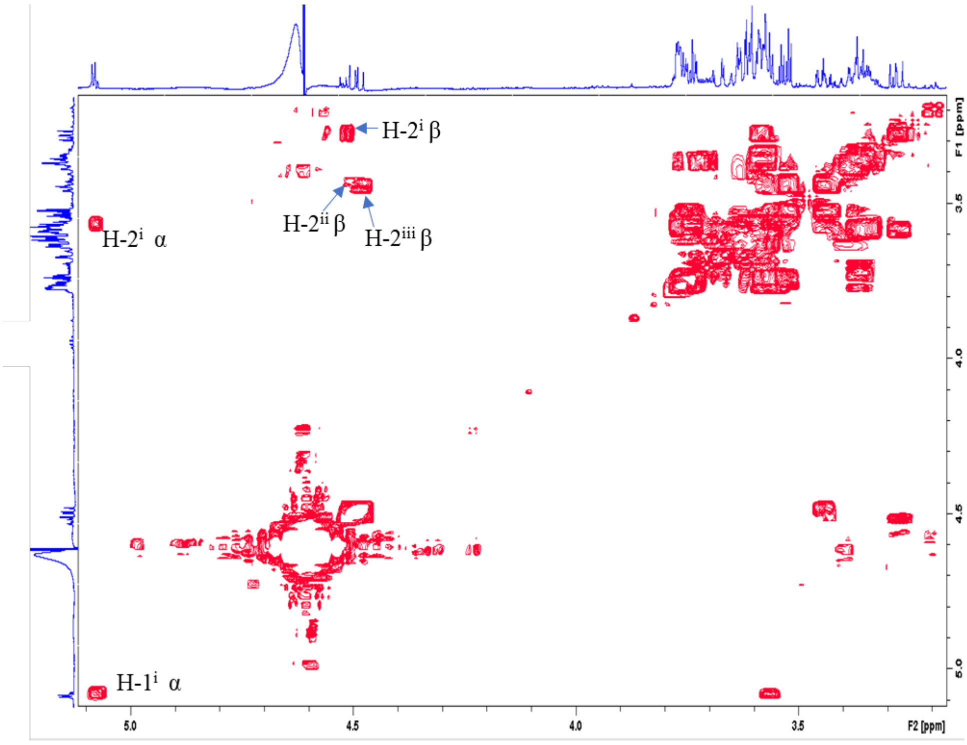

Figure S45. 2D-COSY of β-D-Gal^{iii}-(1→3)-β-D-Glc^{ii}-(1→3)-D-Glc^{i} (**19**). The spectrum was generated in Top Spin v. 3.2.

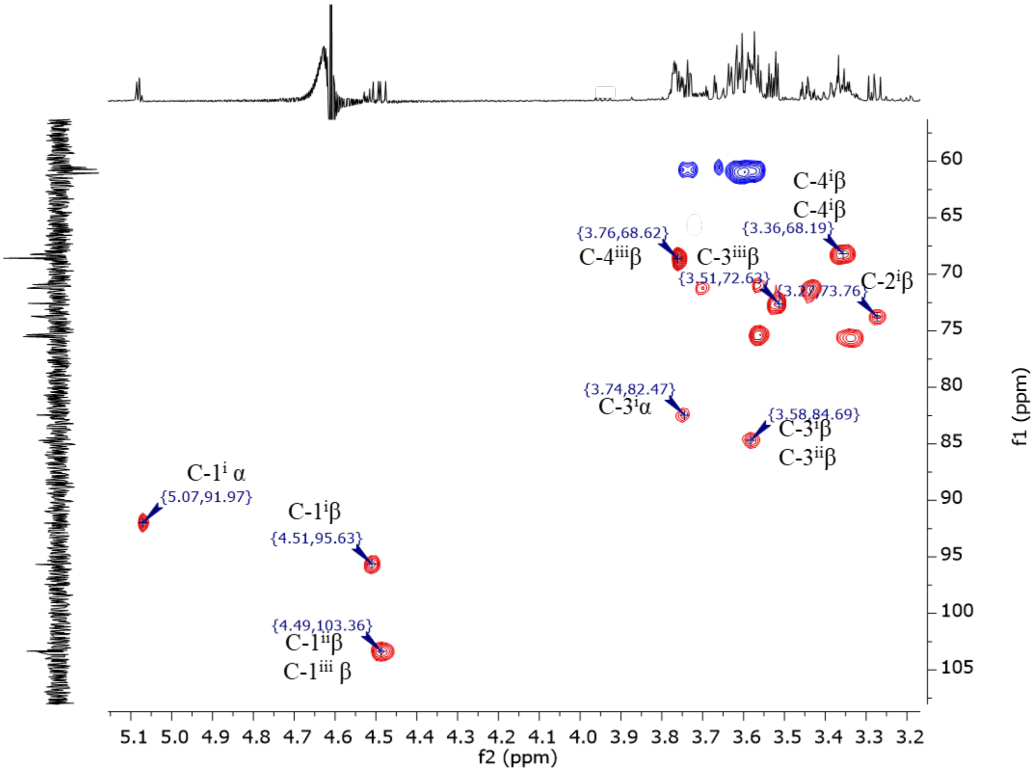

Figure S46. 2D-HSQC of β-D-Gal^{iii}-(1→3)-β-D-Glc^{ii}-(1→3)-D-Glc^{i} (**19**).

β-D-Gal^{iv}-(1→3)-β-D-Glc^{iii}-(1→3)-β-D-Glc^{ii}-(1→4)-D-Glc^{i} (20)

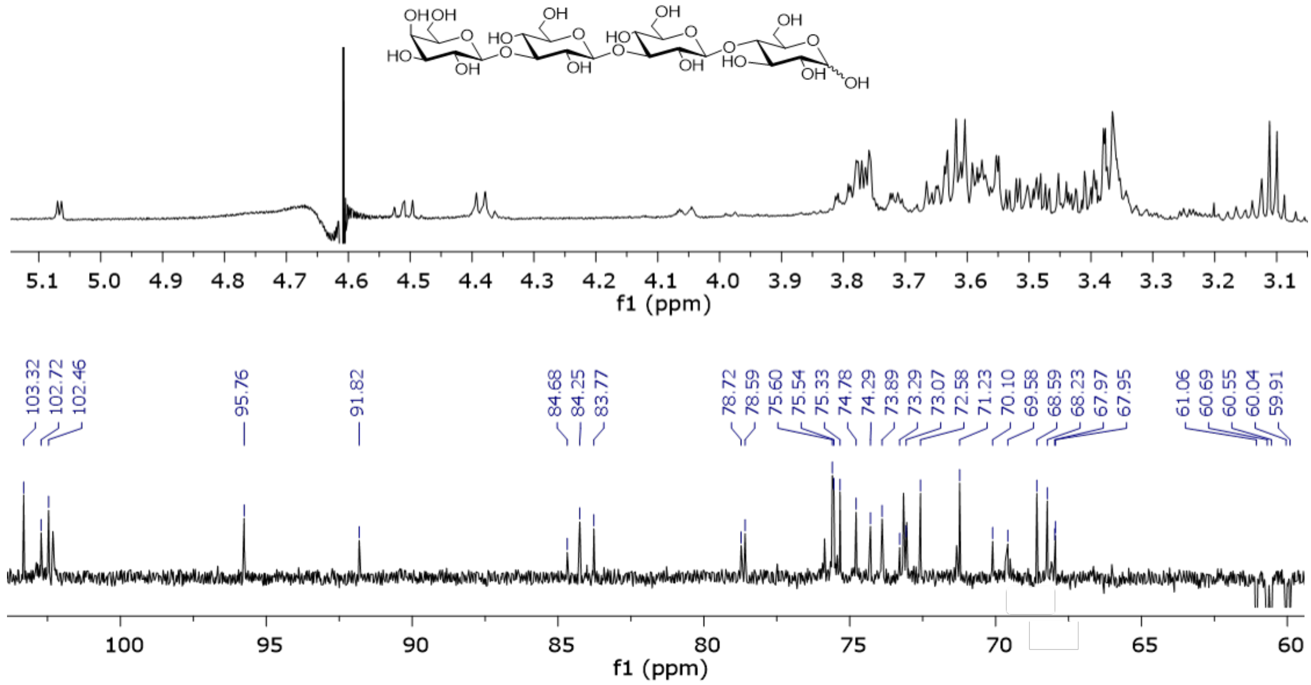

Figure S47. ^{1}H and ^{13}C (DEPT135) NMR (600 MHz, D_2O) of β-D-Gal^{iv}-(1→3)-β-D-Glc^{iii}-(1→3)-β-D-Glc^{ii}-(1→4)-D-Glc^{i} (**20**).

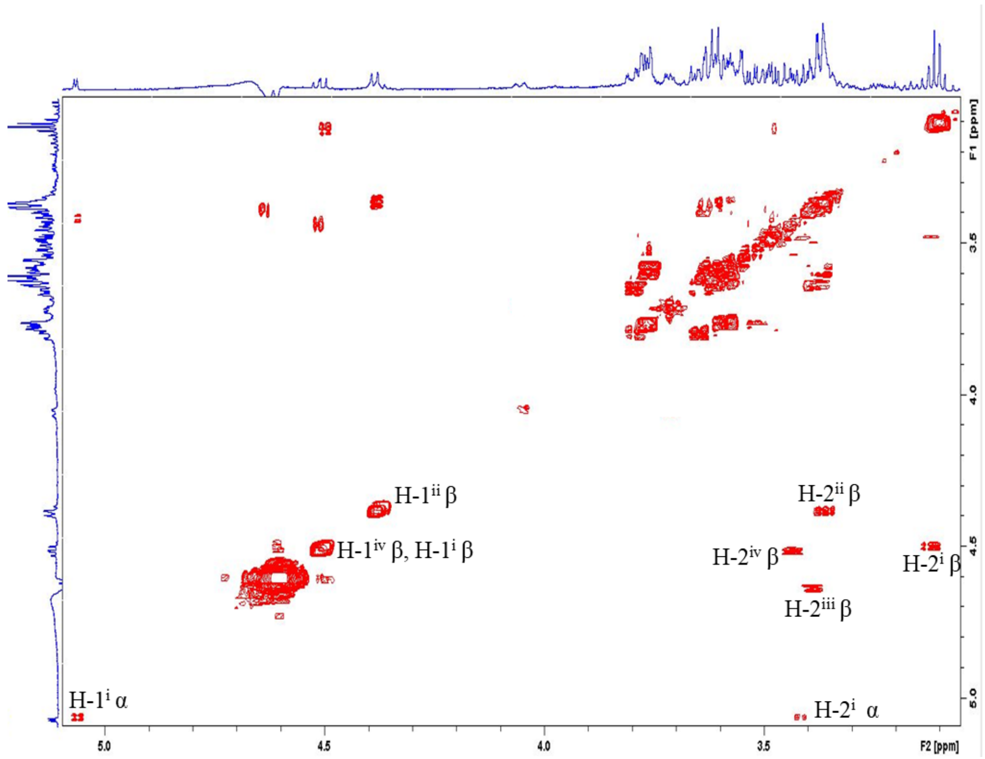

Figure S48. 2D-COSY of β-D-Gal^{iv}-(1→3)-β-D-Glc^{iii}-(1→3)-β-D-Glc^{ii}-(1→4)-D-Glc^{i} (**20**). The spectrum was generated in Top Spin v. 3.2.

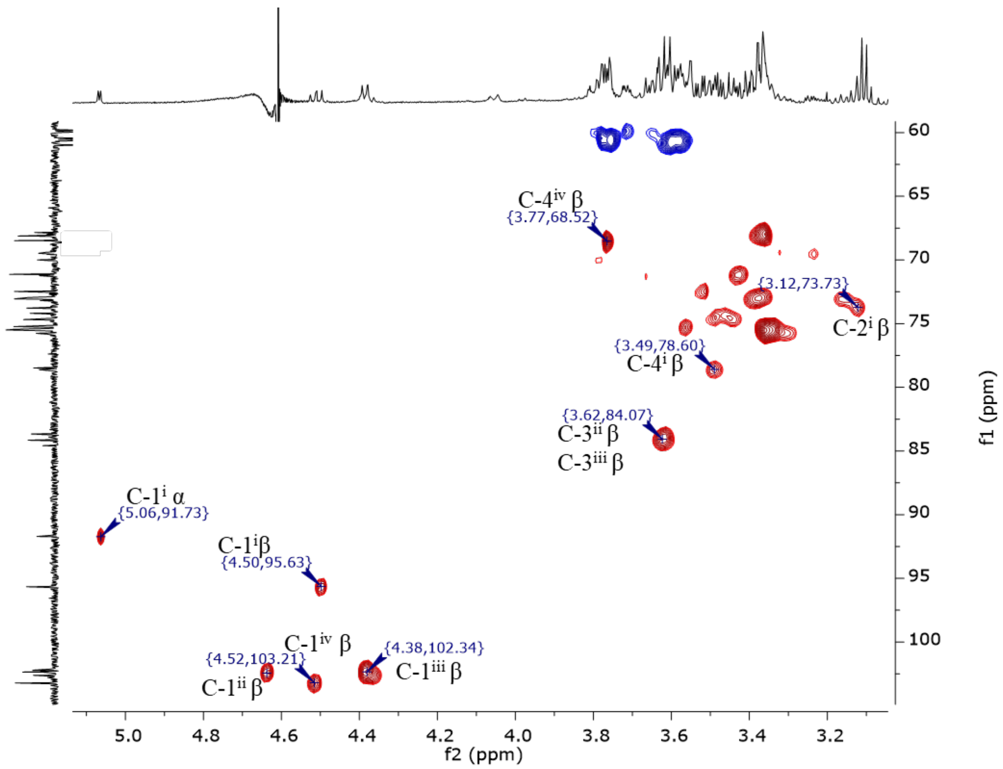

Figure S49. 2D-HSQC of β-D-Gal^{iv}-(1→3)-β-D-Glc^{iii}-(1→3)-β-D-Glc^{ii}-(1→4)-D-Glc^{i} (**20**).

β-D-Gal^{iii}-(1→3)-β-D-Glc^{ii}-(1→4)-D-Glc^{i} (21).

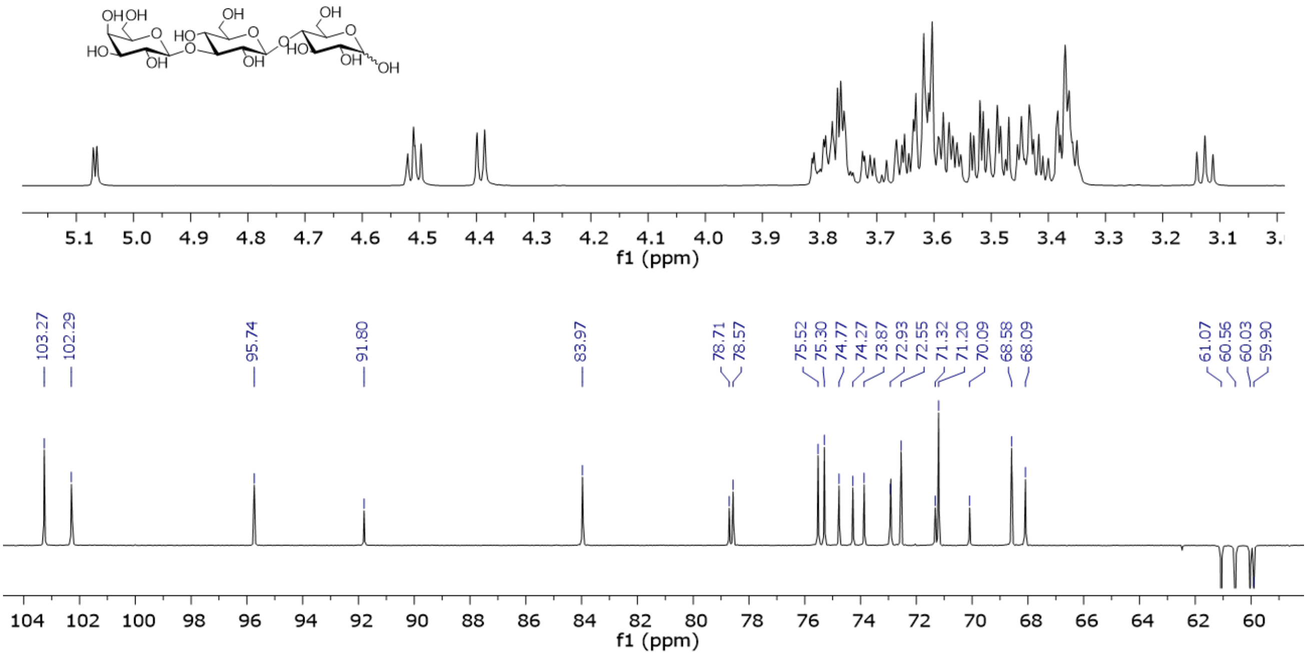

Figure S50. ^{1}H and ^{13}C (DEPT135) NMR (600 MHz, D_2O) of β-D-Gal^{iii}-(1→3)-β-D-Glc^{ii}-(1→4)-D-Glc^{i} (**21**).

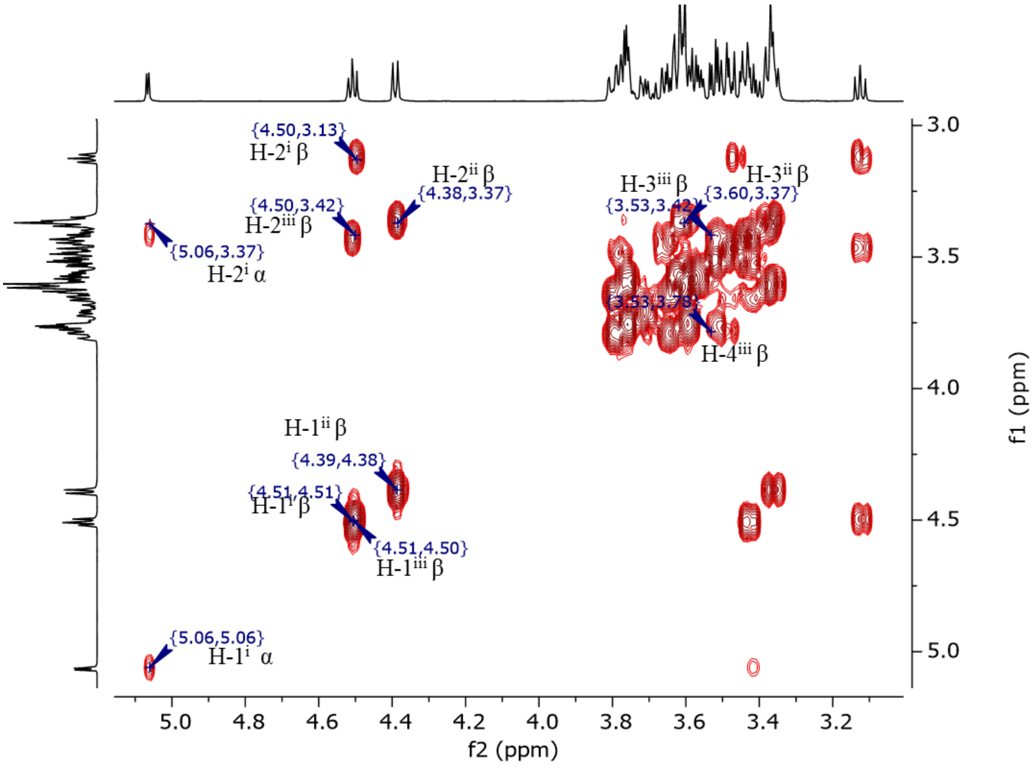

Figure S51. 2D-COSY of β-D-Gal^{iii}-(1→3)-β-D-Glc^{ii}-(1→4)-D-Glc^{i} (**21**).

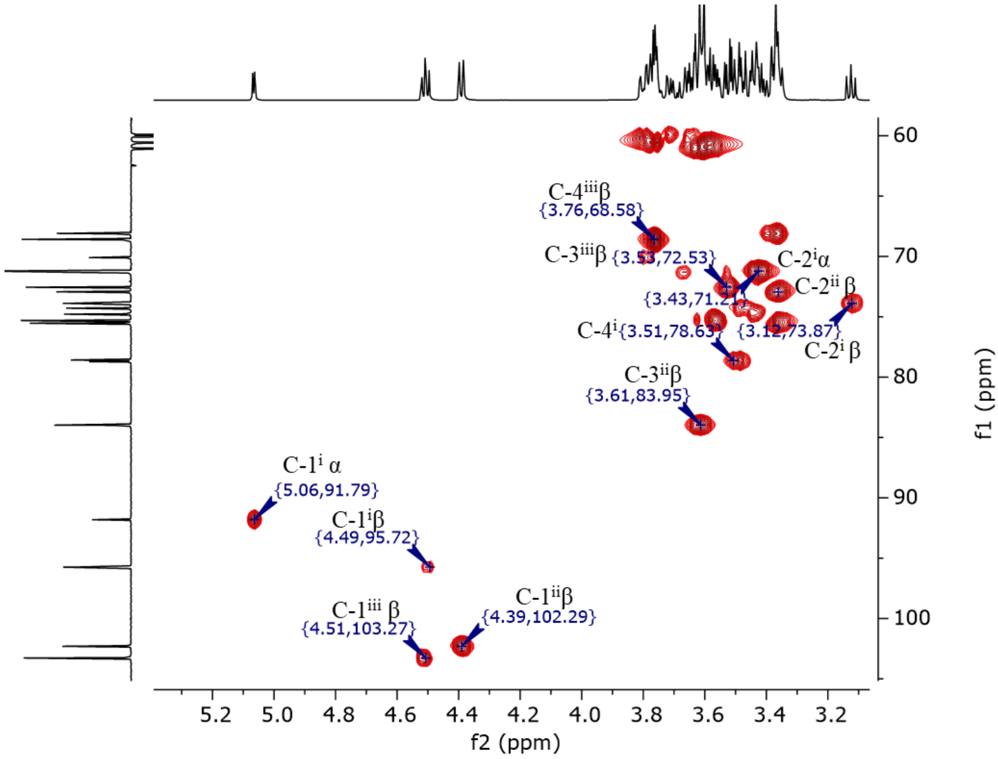

Figure S52. 2D-HSQC of β-D-Gal^{iii}-(1→3)-β-D-Glc^{ii}-(1→4)-D-Glc^{i} (**21**).

β-D-Gal^{iv}-(1→3)-β-D-Glc^{iii}-(1→4)-β-D-Glc^{ii}-(1→4)-D-Glc^{i} (22).

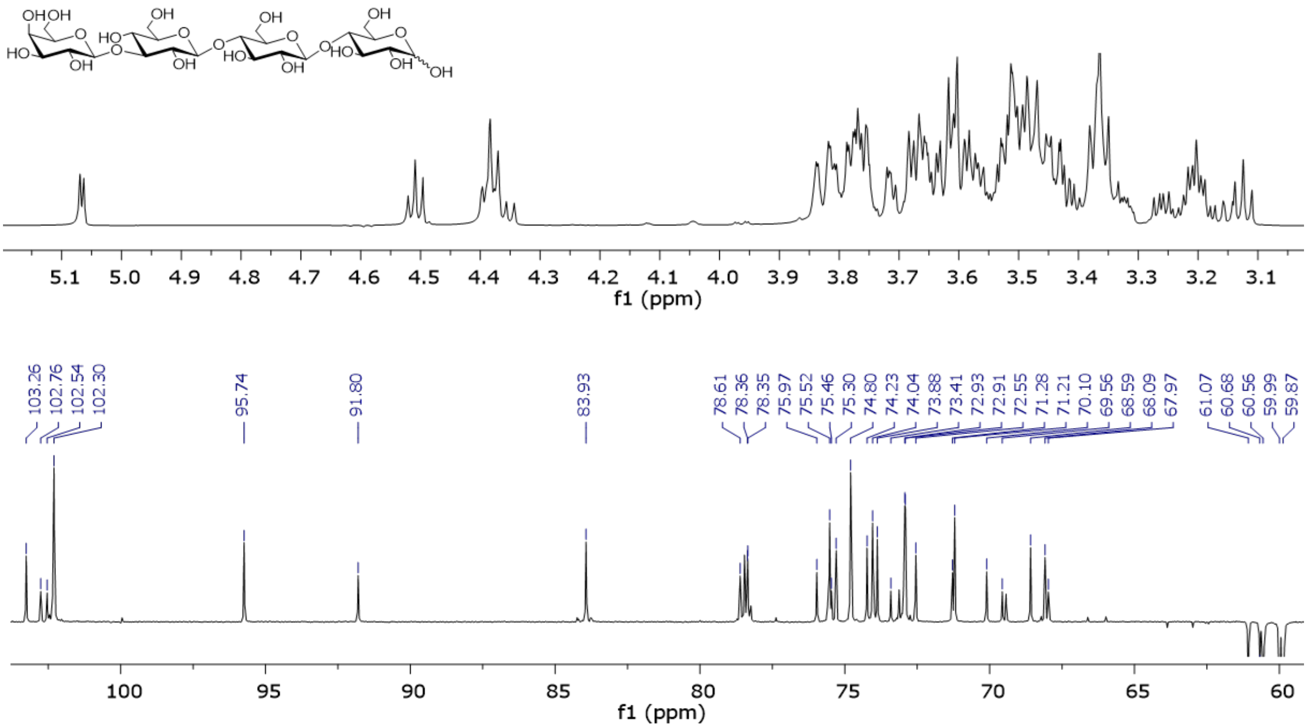

Figure 53. ^{1}H and ^{13}C (DEPT135) NMR(600 MHz, D_2O) of β-D-Gal^{iv}-(1→3)-β-D-Glc^{iii}-(1→4)-β-D-Glc^{ii}-(1→4)-D-Glc^{i} (**22**).

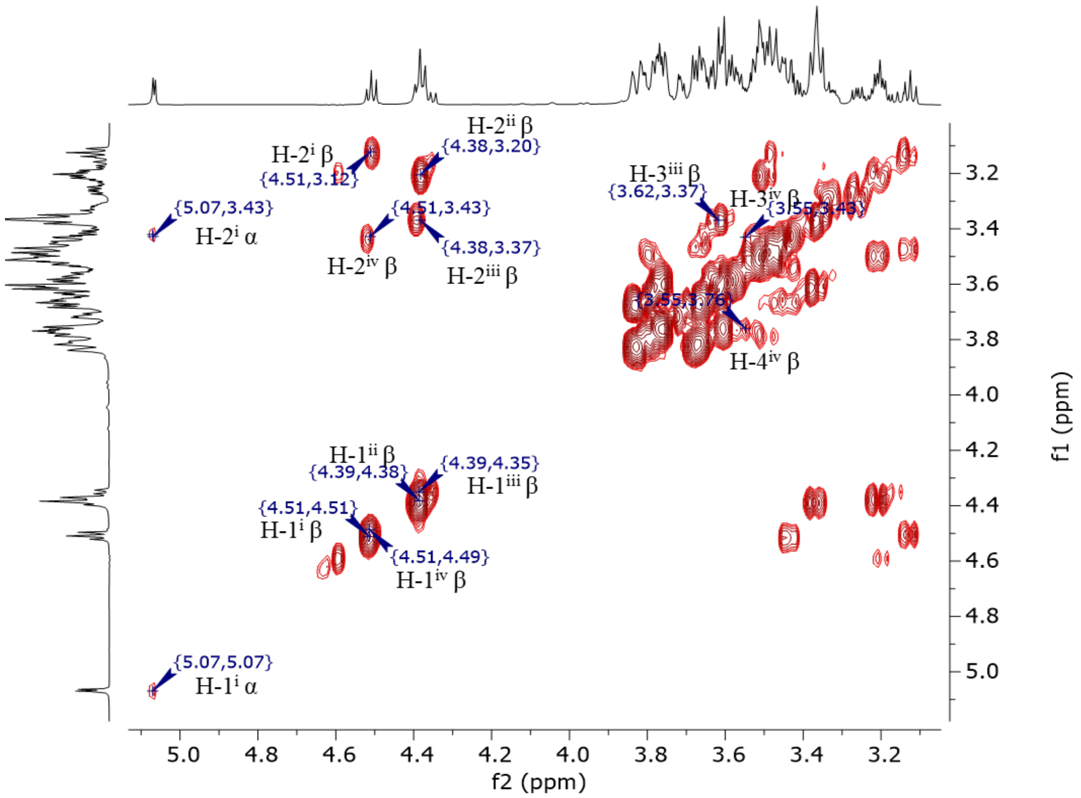

Figure S54. 2D-COSY of β-D-Gal^{iv}-(1→3)-β-D-Glc^{iii}-(1→4)-β-D-Glc^{ii}-(1→4)-D-Glc^{i} (**22**).

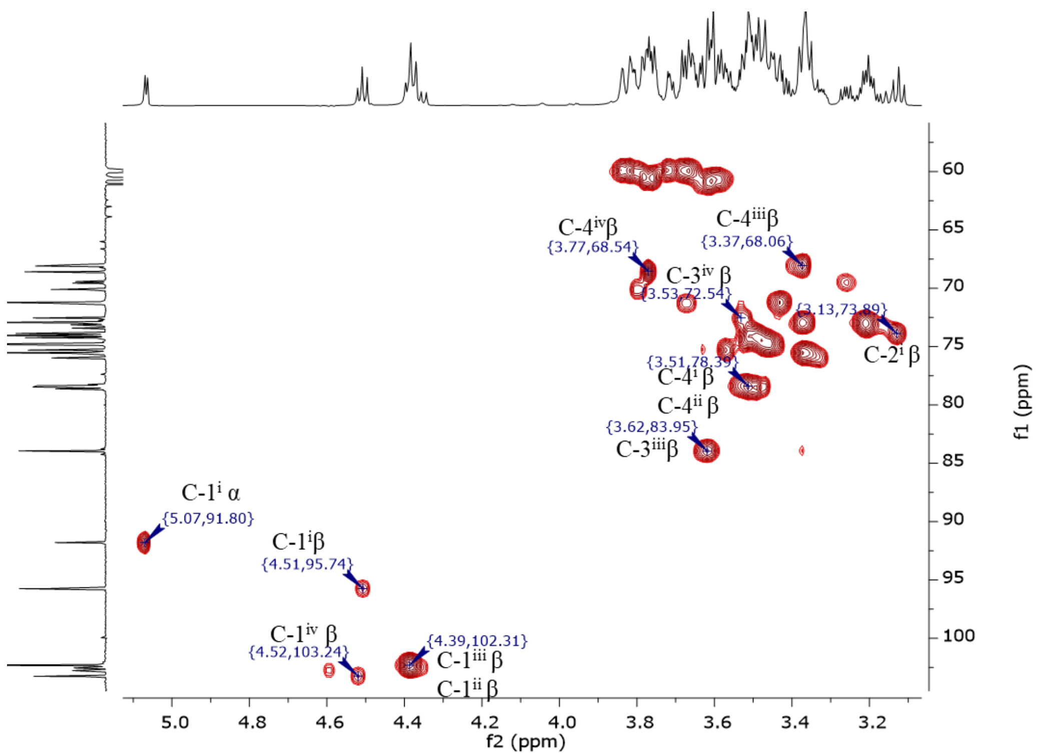

Figure S55. 2D-HSQC of β-D-Gal^{iv}-(1→3)-β-D-Glc^{iii}-(1→4)-β-D-Glc^{ii}-(1→4)-D-Glc^{i} (**22**).

β-D-Gal^{iv}-(1→3)-β-D-Glc^{iii}-(1→4)-β-D-Glc^{ii}-(1→3)-D-Glc^{i} (23)

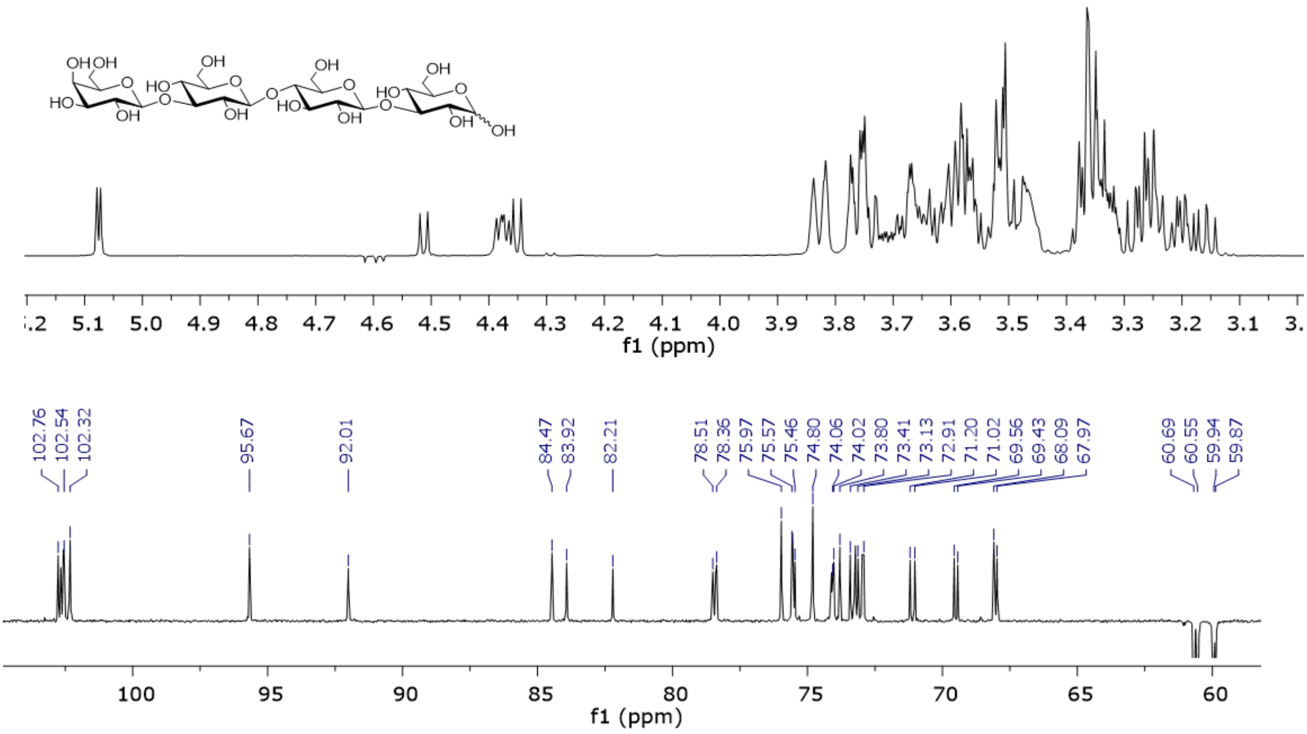

Figure S56. ^{1}H and ^{13}C (DEPT135) NMR (600 MHz, D_2O) of β-D-Gal^{iv}-(1→3)-β-D-Glc^{iii}-(1→4)-β-D-Glc^{ii}-(1→3)-D-Glc^{i} (**23**).

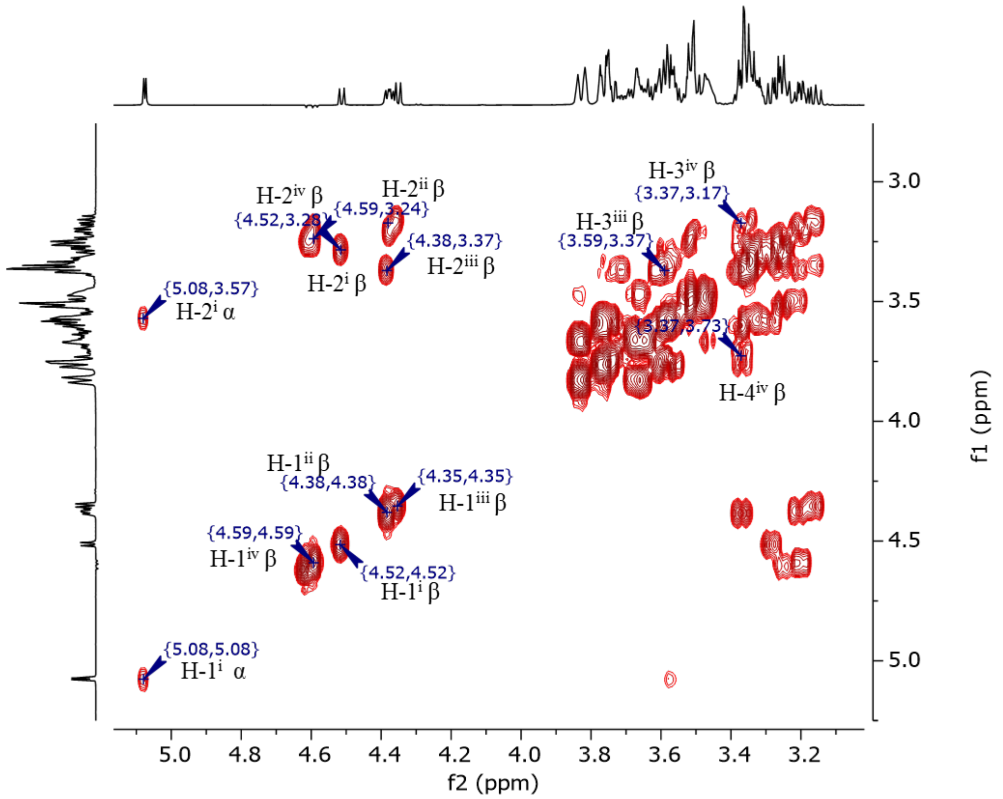

Figure S57. 2D-COSY of β-D-Gal^{iv}-(1→3)-β-D-Glc^{iii}-(1→4)-β-D-Glc^{ii}-(1→3)-D-Glc^{i} (**23**).

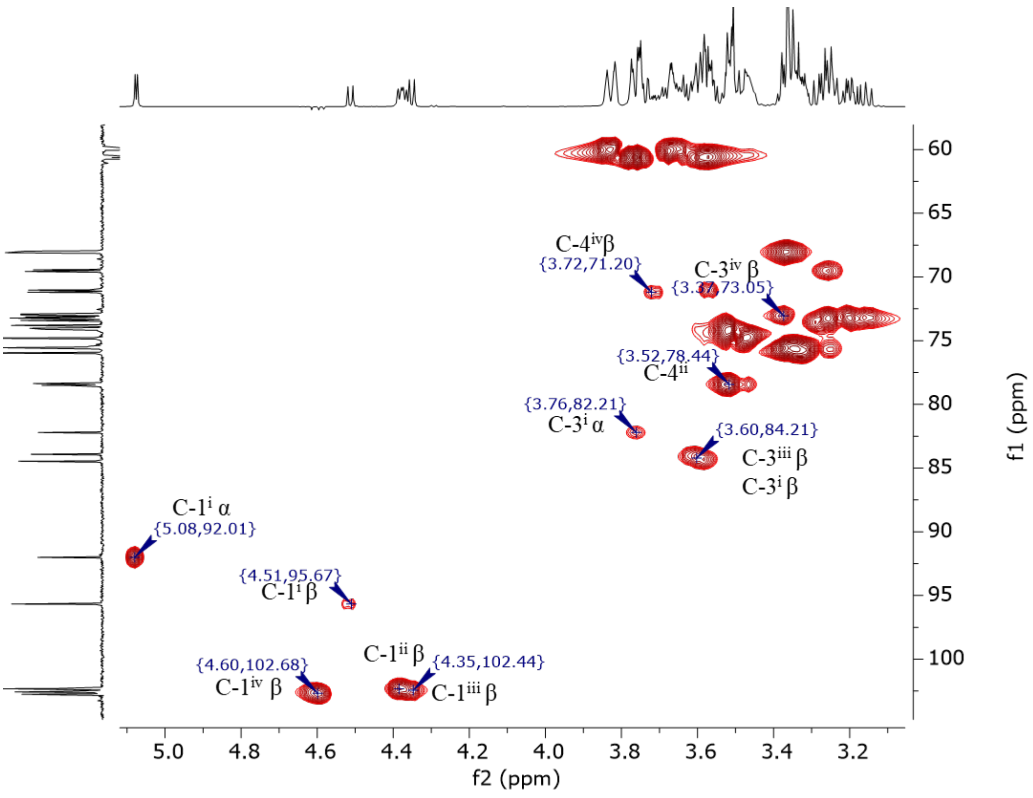

Figure S58. 2D-HSQC of β-D-Gal^{iv}-(1→3)-β-D-Glc^{iii}-(1→4)-β-D-Glc^{ii}-(1→3)-D-Glc^{i} (**23**).

β-D-GlcN^{iii}-(1→3)-β-D-Glc^{ii}-(1→4)-D-Glc^{i} (24)

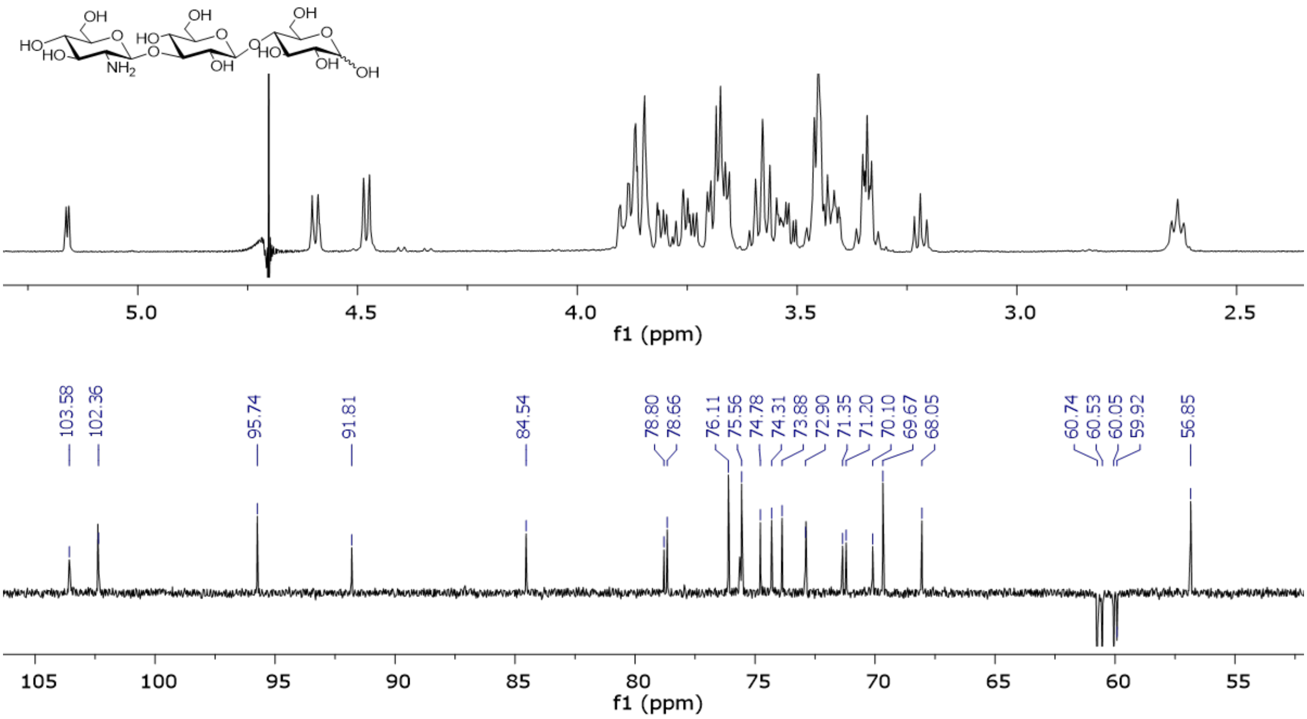

Figure S59. ^{1}H and ^{13}C (DEPT135) NMR (600 MHz, D_2O)of β-D-GlcN^{iii}-(1→3)-β-D-Glc^{ii}-(1→4)-D-Glc^{i} (**24**).

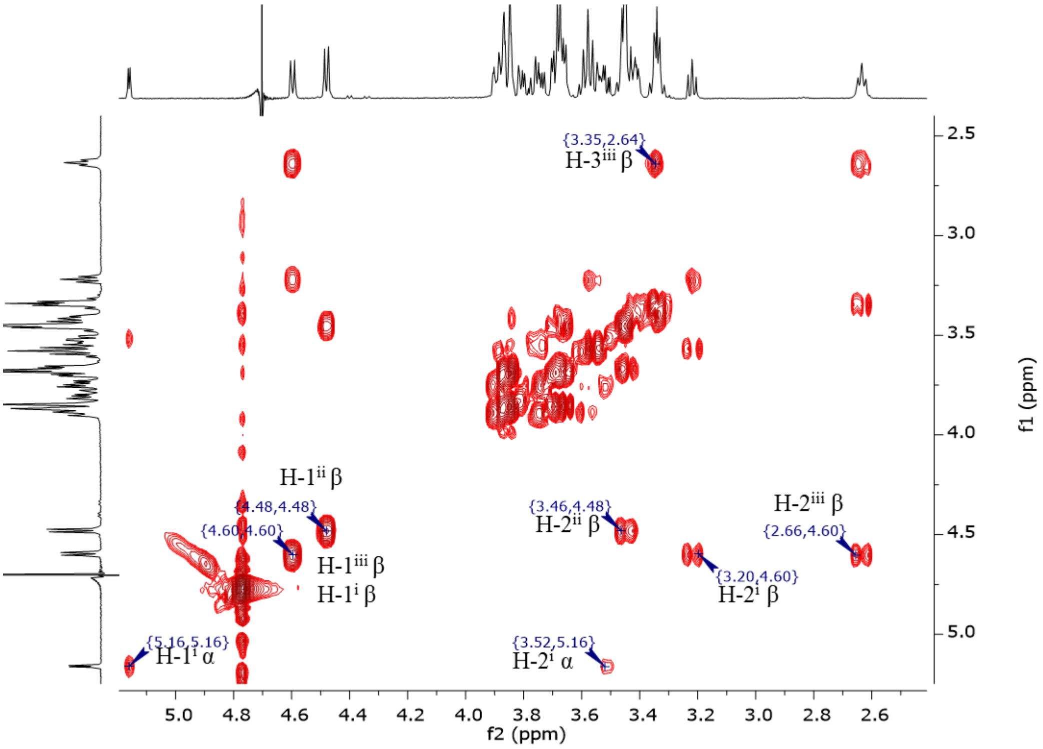

Figure S60. 2D-COSY of β-D-GlcN[iii]-(1→3)-β-D-Glc[ii]-(1→4)-D-Glc[i] (**24**).

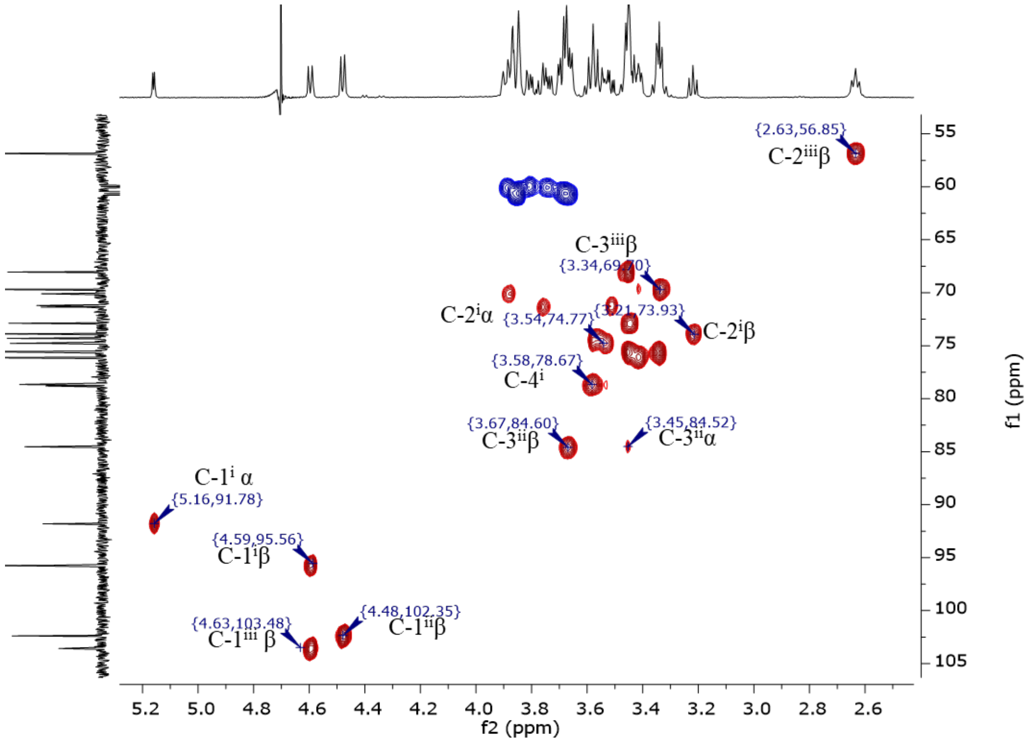

Figure S61. 2D-HSQC of β-D-GlcN[iii]-(1→3)-β-D-Glc[ii]-(1→4)-D-Glc[i] (**24**).

β-D-Man^{iii}-(1→3)-β-D-Glc^{ii}-(1→4)-β-D-Glc^{i} (25)

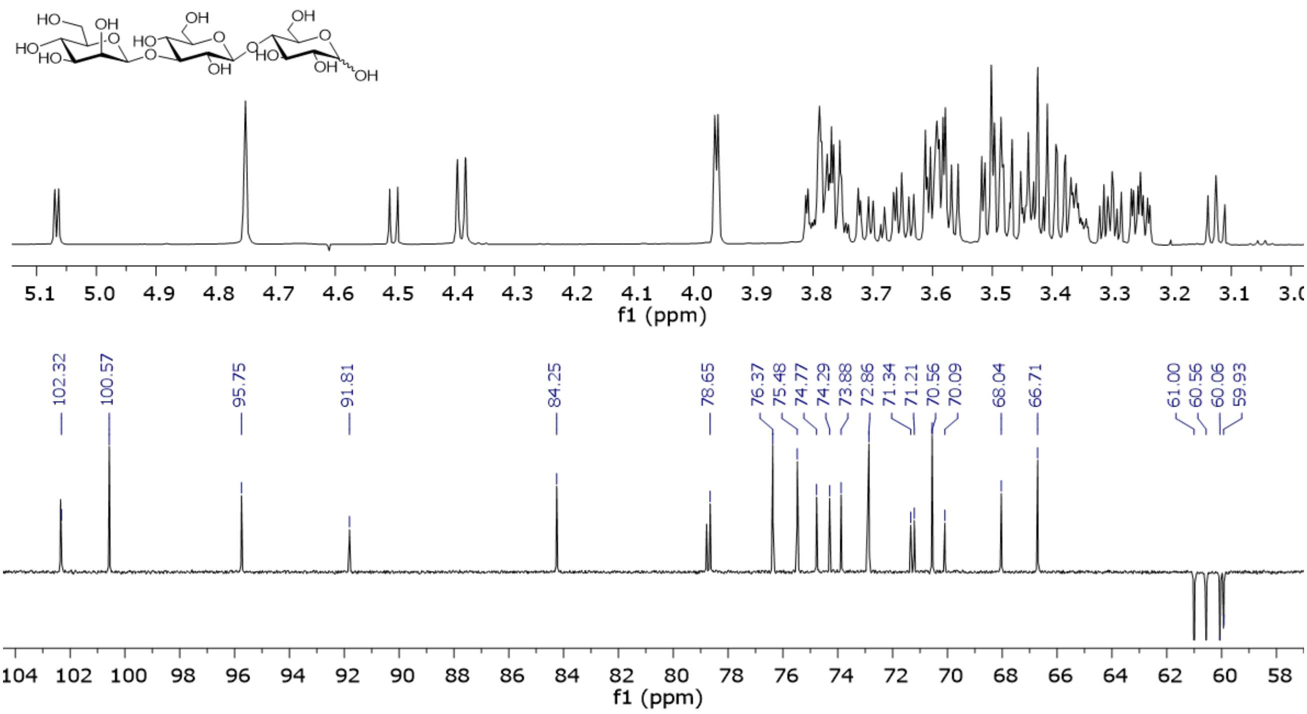

Figure S62. ^{1}H and ^{13}C (DEPT135) NMR (600 MHz, D_2O) of β-D-Man^{iii}-(1→3)-β-D-Glc^{ii}-(1→4)-β-D-Glc^{i} (**25**).

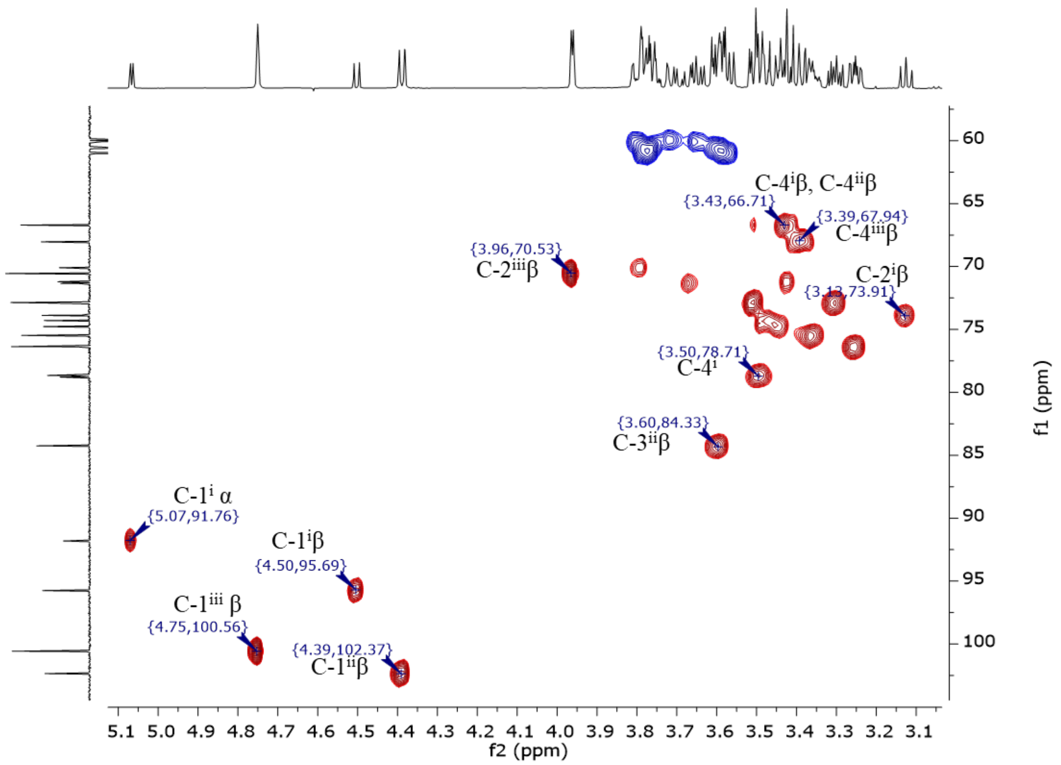

Figure S63. 2D-HSQC of β-D-Man^{iii}-(1→3)-β-D-Glc^{ii}-(1→4)-β-D-Glc^{i} (**25**).

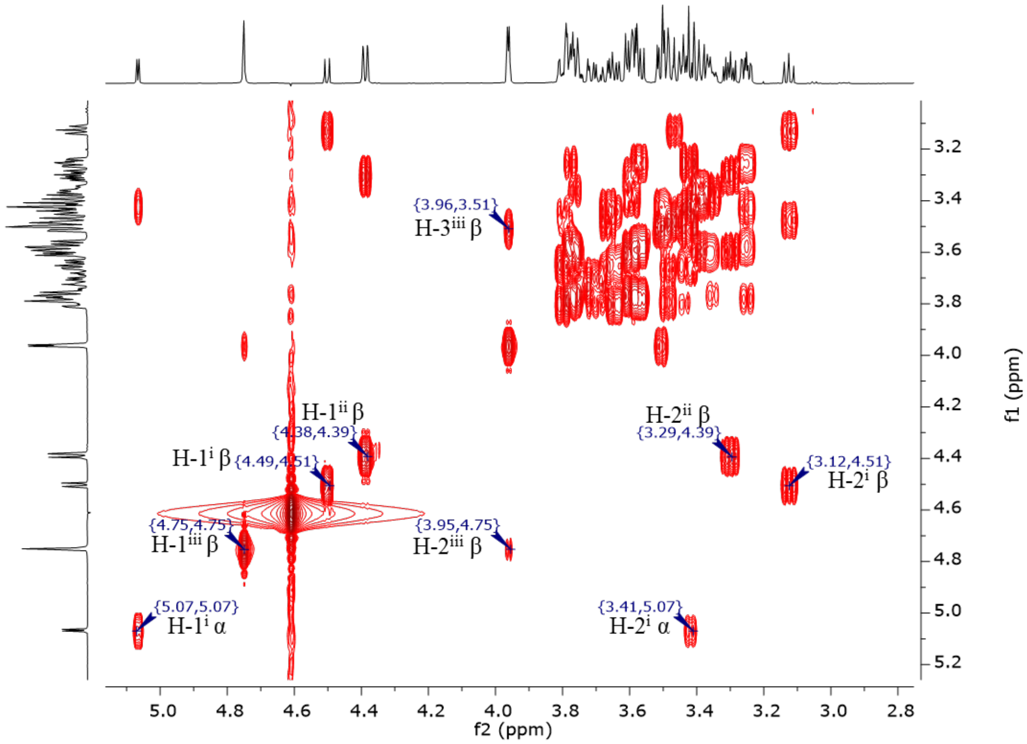

Figure S64. 2D-COSY of β-D-Man^{iii}-(1→3)-β-D-Glc^{ii}-(1→4)-β-D-Glc^{i} (**25**).
